# Click Chemistry and Multicomponent Reaction for Linker Diversification of Zinc Dipicolylamine-Based Drug Conjugates

**DOI:** 10.3389/fchem.2021.822587

**Published:** 2022-02-15

**Authors:** Ching-Hua Tsai, Tai-Yu Chiu, Chiung-Tong Chen, Chia-Yu Hsu, Ya-Ru Tsai, Teng-Kuang Yeh, Kuan-Hsun Huang, Lun Kelvin Tsou

**Affiliations:** Institute of Biotechnology and Pharmaceutical Research, National Health Research Institutes, Miaoli, Taiwan

**Keywords:** zinc dipicolylamine, small-molecule drug conjugates, phosphatidylserine, tumor microenvironment, click chemistry

## Abstract

An efficient Ugi multicomponent reaction with strain promoted azide-alkyne cycloaddition protocol has been utilized in concert or independently to prepare a small family of bioactive zinc(II) dipicolylamine (ZnDPA)-based SN-38 conjugates. With sequential click chemistry coupling between the cytotoxic payload and phosphatidylserine-targeting ZnDPA ligand derived from structurally diverse carboxylic acids, aldehyde or ketones, and isocyanides, we demonstrated that this convergent synthetic strategy could furnish conjugates harnessing diversified linkers that exhibited different pharmacokinetic profiles in systemic circulation *in vivo*. Among the eight new conjugates, comparative studies on *in vitro* cytotoxicities, plasma stabilities, *in vivo* pharmacokinetic properties, and maximum tolerated doses were then carried out to identify a potent ZnDPA-based SN-38 conjugate that resulted in pancreatic cancer growth regression with an 80% reduction of cytotoxic payload used when compared to that of the marketed irinotecan. Our work provided the roadmap to construct a variety of theranostic agents in a similar manner for cancer treatment.

## Introduction

Ligand-targeted therapeutic has become an effective precision medicine strategy for cancer treatment (Srinivasarao et al., 2015; Srinivasarao and Low, 2017). In particular, attaching chemotherapeutic drug through a linker to antibodies has become a clinical strategy to selectively deliver a potent cytotoxic compound to tumor cells while alleviating majority of adverse side effects systemically that might result from the conventional cancer chemotherapy (Hayat and Sahebkar, 2020; Ponziani et al., 2020). Indeed, there were several antibody drug conjugates (ADCs) reaching the market in the past 5 years, and spurred by the clinical success of these ADCs (Nessler et al., 2021), emerging alternative strategy in small molecular drug conjugate (SMDC) has provided a new perspective for targeted drug delivery (Cazzamalli et al., 2018a; Cazzamalli et al., 2018b; Zhuang et al., 2019). SMDC can be advantageous with manageable synthesis during development, less immunogenic in nature, easier optimization of pharmacokinetic properties, and lower molecular weights that might confer better solid tumor penetrations (Casi and Neri, 2015; Cazzamalli et al., 2018b; Zhuang et al., 2019). Ranging from prostate-specific membrane antigen (Sanna and Sechi, 2012), carbonic anhydrase IV (Krall et al., 2014), somatostatin derivatives for neuroendocrine tumors (Sun et al., 2008), and folate receptors (Leamon et al., 2007), respective SMDCs are under development in the pre-clinical stage or undergoing clinical trials for treating different cancer diseases.

With complex interplay between tumors and their microenvironment governing cancer progression, we have synthesized and evaluated zinc(II) dipicolylamine (ZnDPA)-based drug conjugates to target tumor-associated phosphatidylserine (PS) in the tumor microenvironment (Liu et al., 2017; Liu et al., 2019; Chang et al., 2020). PS is an essential component of cell membranes and normally segregated in the inner leaflet; yet, several studies have identified increased amount of externalized PS on the surface of tumor cells in the tumor microenvironment (Laforest et al., 2017; Sharma and Kanwar, 2018). Concurrently, a study also determined that enrichment of PS on the surface of tumor cell-derived microvesicles could result innate immunosuppressive properties that allow tumor growth and metastasis (Lima et al., 2009; Matsumura et al., 2019). Thus, PS exposure at the tumor microenvironment has become an imaging tool or a novel biomarker for cancer (Laforest et al., 2017; Wang et al., 2017). The antibody bavituximab that targets PS has been developed, and together with the use of chemotherapy or radiation, it is under clinical trial investigation for the treatment of various cancers (Gerber et al., 2015; Gerber et al., 2018; Mokdad et al., 2019). On the other hand, pioneered by Smith et al., accumulation of small-molecule ZnDPA-derived fluorescent ligands at the tumor site through its interaction with negatively charged PS was readily observed (Smith et al., 2010; Smith et al., 2013; Rice et al., 2016). Our previous work also demonstrated that synthetic PS-targeting ZnDPA in conjugation with a potent cytotoxic agent SN-38 possessed *in vivo* efficacies against various cancers (Chen et al., 2021).

As an SMDC consists of three major components, targeting ligand, linker, and cytotoxic payload, optimization of each part can influence the eventual efficacy of the whole conjugate (Srinivasarao and Low, 2017). From our previous reports, we have followed linear synthetic routes for the preparation of ZnDPA conjugates, which made the linker diversifications and conjugate purifications laborious. The properties of the linker moiety within the conjugate plays a key role in developing a safe and effective SMDC (Zhuang et al., 2019). A well-designed linker not only plays critical roles in conjugate’s solubility and stability during synthetic preparation and storage, but also conveys stability for systemic circulation *in vivo*. This linkage is designed to facilitate targeting ligand to selectively deliver the cytotoxic payload by preventing premature release of the cargo before reaching the tumor sites. In the current report, we report a facile convergent synthetic strategy to prepare bioactive ZnDPA-based SN-38 conjugate through (1) the incorporation of the Ugi four-component reaction (U-4CR) that harnesses good reactivity and has been widely applied in diversity-oriented synthetic strategies for linker diversification in these conjugates; (2) employing strain promoted azide-alkyne cycloaddition (SPAAC) for facile cytotoxic payload conjugation; (3) evaluation of conjugates’ structure–activity relationships (SAR) in plasma stability and cytotoxicities between different linker moieties; and (4) determination of conjugates’ *in vivo* pharmacokinetic properties with respect to its anti-tumor efficacy *in vivo*.

## Materials and Methods

### *In vitro* Plasma Stability Study

Six-week-old male ICR mice were sacrificed with blood samples collected through cardiac puncture into EDTA tubes placed on ice. Plasma samples were collected by centrifugation (3000 rpm for 15 min at 4°C) and stored frozen at −20°C until use. For the conjugate’s stability study, plasma aliquots of 70 μl were added to 0.6-ml microcentrifuge tubes (Basic Life, Taipei, Taiwan). Each conjugate was dissolved in a mixture of DMSO and D5W (1:9, v/v), and the mixture (70 μl each) was then added into the plasma tubes. After a 6-h incubation (*n* = 3 for each incubation time) at 37°C in a water bath, samples were subjected to HPLC analysis to determine the levels of intact conjugate and SN-38 released.

### Cells and Culture Medium

MiaPaCa-2 cells, obtained from the Bioresource Collection and Research Center, Hsinchu, Taiwan, and the Detroit 551 cells were cultured in Dulbecco’s modified Eagle’s medium (DMEM). All cells were grown in a humidified CO_2_ incubator at 37°C. Cell viability was determined by the MTS assay. In brief, cells were cultured (2,500–3,000 cells/well) in flat bottomed 96-well plates for 24 h. Serial-diluted conjugates were added to the cell media and incubated for 72 h. Culture medium was removed after 72 h followed by the addition of 100 μl of MTS and PMS mixture solution. In a humidified incubator at 37°C with 5% CO_2_, cells were then incubated for another 1.5 h for viable cells to convert the tetrazolium salt into formazan. The conversion to formazan was monitored at the 490-nm absorbance by a BioTek PowerWave-X Absorbance Microplate Reader. In this MTS assay, the recorded data were normalized to DMSO-treated and background controls. Using GraphPad Prism version 4 software, the IC_50_ value was the amount of each conjugate that caused a 50% reduction in cell viability in comparison to DMSO-treated controls.

### *In vivo* Pharmacokinetic Studies

Six-week-old ICR mice, purchased from Biolasco (Taiwan), were divided into groups of three and 100 µl of dosing solution was given intravenously. Through cardiac puncture, blood sample was collected from each sacrificed animal at time points at 0.003, 0.083, 0.25, 0.5, 1, 2, 4, 6, 8, and 24 h and stored on ice (0–4°C). Plasma was separated from the blood by centrifugation at 3,000 rpm for 15 min at 4°C in a Beckman Model Allegra 6R centrifuge. Analysis of the plasma samples were done by using liquid chromatography with tandem mass spectrometry (LC/MS/MS). A gradient system was used for the separation of analyte and IS. Ammonium acetate aqueous solution (10 mM) containing 0.1% formic acid was used as mobile phase A, while mobile phase B was acetonitrile. The gradient profile was as follows: 0.0–1.1 min, 50%B; 1.2–3.7 min, 55%B–90%B; 3.8–5.0 min, 90%B–50%B at the flow rate of 1.5 ml/min. The autosampler was programmed to inject 15 µl of sample every 5 min.

### *In vivo* Maximum Tolerated Dose Determination Studies

The acute toxicity studies were determined in ICR male mice. In general, the studies were conducted using the dose of 10-50 mpk for dosing 5 consecutive days and the survival of the animals and their body weight were monitored for 14 days after first dosing.

### Materials, Preparation, Inoculation of Tumor Cells, and *in vivo* Anti-Tumor Studies

Sources of the mice, cell lines, and materials used for the *in vivo* anti-tumor studies were as follows: 8-week-old athymic NU-Fox1^nu^ mice, male (BioLASCO, Ilan, Taiwan), CPT-11 (Herocan^®^, Lot. 1B3130, Nang Kuang Pharmaceutical Co., Ltd., Tainan, Taiwan), dimethyl sulfoxide (DMSO) (D1435, Sigma-Aldrich, St. Louis, MO, United States), cremophor EL (C5135, Sigma-Aldrich, St. Louis, MO, United States), dextrose injection 5% (Tai Yu Chemical and Pharmaceutical, Hsinchu, Taiwan), sodium chloride 0.9% inj., saline (Tai Yu Chemical and Pharmaceutical, Hsinchu, Taiwan), Matrigel™ (356237, BD Biosciences, San Jose CA, United States), RPMI 1640 Medium (31800-022, Thermo Fisher Scientific Inc., Waltham, MA, United States), RPMI 1640 Medium, no phenol red (11835-030, Thermo Fisher Scientific Inc., Waltham, MA, United States), fetal bovine serum (04-001-1A-US, Biological Industries, Beit Haemek, Israel), 1-ml syringe (Terumo Medical Corp., Laguna, Philippines), needle 24G × 1/2 inch (Terumo Medical Corp., Yamanashi Prefecture, Japan), needle 27G × 1/2 inch (Terumo Medical Corp., Yamanashi Prefecture, Japan), digital caliper (FOW54-200-777, PRO-MAX, Newton, Massachusetts, United States), animal holder (mouse, Yeong Jyi Chemical Apparatus Co., Ltd., Sanchong, Taiwan), Attane Isoflurane (Panion and BF Biotech Inc., Taipei, Taiwan), D-Luciferin potassium salt (PerkinElmer, Waltham, MA, United States), IVIS spectrum system (Caliper Life Sciences, Inc., Hopkinton, MA, United States), and 100% CO_2_ gas (Sinda Gases, Hsinchu, Taiwan).

The number of viable cells was counted with a hemocytometer with trypan blue staining under a light microscope on the day of tumor cell inoculation. Cells were suspended with phenol red free RPMI-1640 and Matrigel in a 1:1 ratio. MiaPaca-2 suspension cells (1 × 10^6^ cells) were implanted subcutaneously into the left flank of the nude mice using a 1-ml syringe (needle 24G × 1 in., 0.55 × 25 mm; TERUMO). A digital caliper was used to measure tumor sizes and dimensions as the tumor volume in mm^3^ was calculated with the formula: Volume = (length × width^2^)/2. Tumor-bearing mice were randomized (*n* = 8 per group) and mean tumor volume was approximately 170 mm^3^. With the indicated regimen, treatment was started with conjugate **4** (dissolved in 10%DMSO/20% Cremophor/70% of 5% dextrose solution) and CPT-11, which was intravenously (i.v.) administered at 10 mg/kg, qd (5 + 5) or 40 mg/kg, twice/week for 2 weeks, respectively. Body weight and tumor size were measured twice weekly.

## Results and Discussion

**SCHEME 1:**
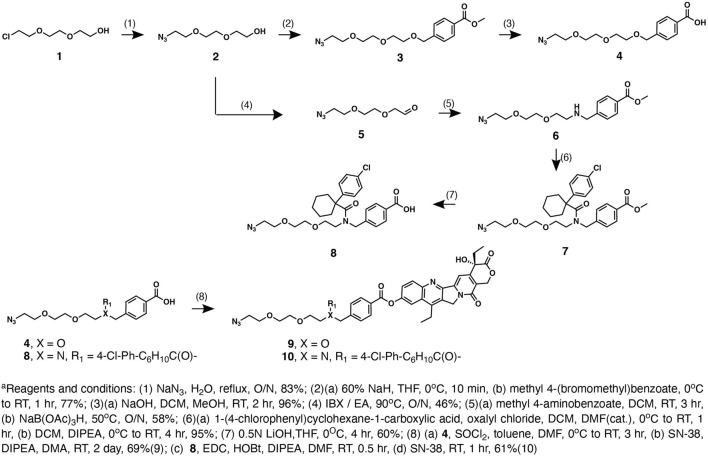
Synthetic routes for azide-functionalized linker-SN-38 **9** and **10.**

In the light of developing new ZnDPA-based drug conjugates, we have synthesized these conjugates to target pancreatic cancer as it is one of the leading causes of cancer-related deaths with very poor prognosis (Hu et al., 2021). Potent topoisomerase-1 inhibitor SN-38 belongs to the class of camptothecins that comprises an array of FDA-approved anticancer bioactive compounds for the treatment of several cancers (Liu et al., 2015). Moreover, SN-38 is an active metabolite of irinotecan with which the nanoliposomal formulation was approved for pancreatic cancer treatment (Chiang et al., 2016). As CPT-11 is behaving as a solubility-enhancing prodrug, SN-38’s bioactivity was first masked during systemic circulation *in vivo*, and readily activated through enzymatic cleavage while engaging in the tumor microenvironment. Herein, our synthesis started with the conjugation to the 10-hydroxyl position of SN-38 with a hydrophilic PEG linker that harnessed a terminal azide on the other distal end. As one of the advantages lies in the cost down of organic theranostic, herein, we designed a convergent synthesis of these conjugates between facile linker modifications devised from multicomponent reaction and simple conjugation of the payload to the ZnDPA targeting ligand. As shown in Scheme 1, synthesis of azide-containing linker-SN38 with different functional groups at R_1_ position was performed *via* eight steps. Sodium azide treatment of **1** followed by addition of methyl 4-(bromomethyl)benzoate gave compound **3**, which was hydrolyzed with NaOH to afford linker intermediate **4**. Oxidation of PEG linker **2** afforded linker **5**, which was then joined by methyl 4-aminobenzoate by reductive amination in 58% yield to furnish linker precursor **6**. If the linker in the conjugate was not stable enough, to allow the conjugate to accumulate at the tumor site, premature release of SN-38 should not confer the desired efficacy. On the other hand, our previous report demonstrated that as the conjugate was too stable in its pharmacokinetic profile, it could result in insufficient release of SN-38 and failed to exert the expected antitumor effect. Therefore, we have identified that cyclohexyl-para-chlorophenyl periphery adjacent to SN-38 could facilitate the control release of SN-38 *in vivo* (Liu et al., 2019). Hence, we carried out condensation between 1-(4-chlorophenyl) cyclohexane-1-carboxylic acid and precursor **6** followed by LiOH hydrolysis, which gave linker **8**. SN-38 was then coupled to either linker **4** or linker **8** to generate the azido-linker-SN-38 **9** or **10**, respectively.

**SCHEME 2:**
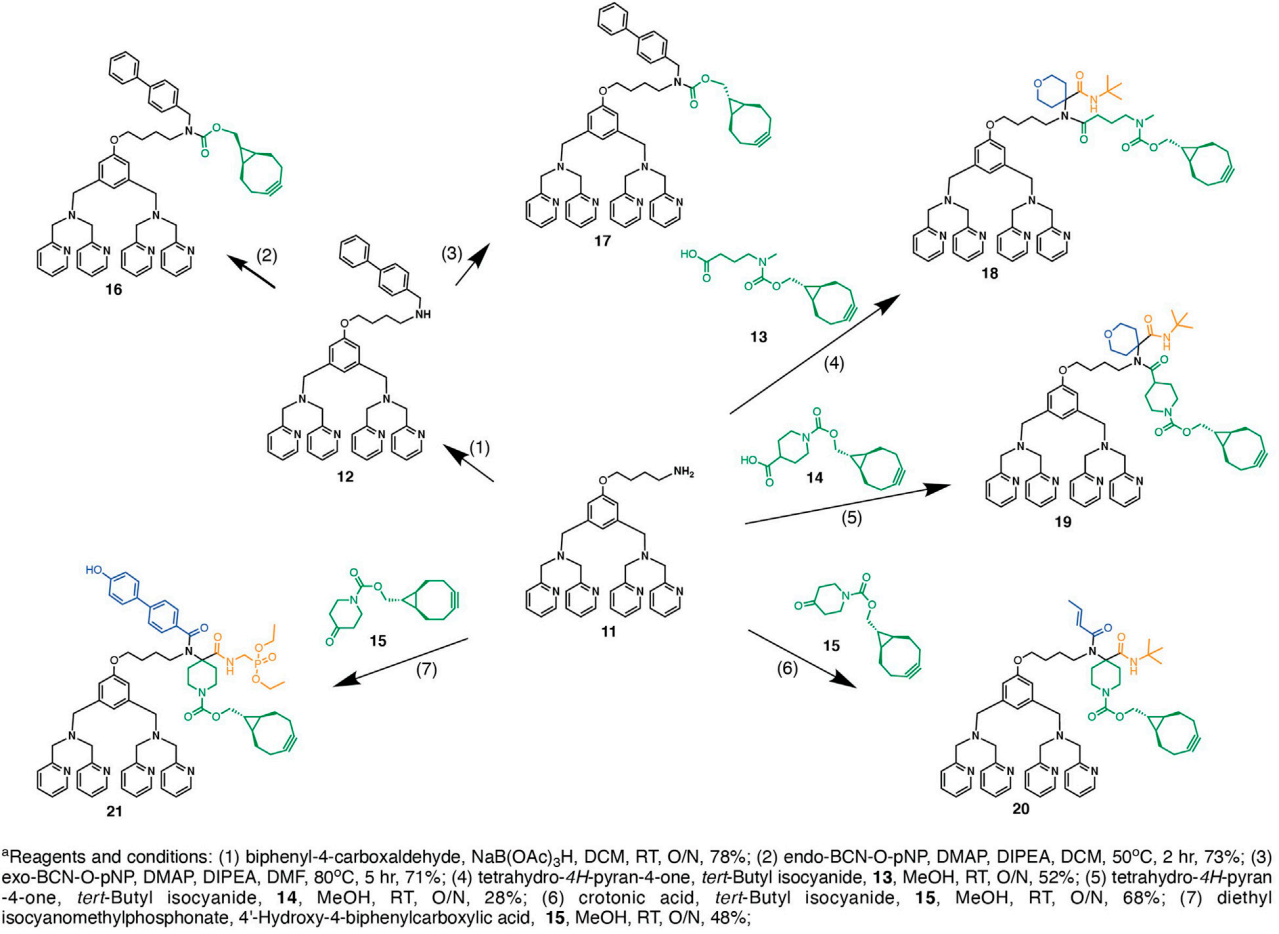
Preparation of intermediates **16–21** using reductive reaction and/or U-4CR

We hypothesize that the multicomponent Ugi reaction should allow facile access of different linker combinations that could lead to different pharmacokinetic profiles. In particular, we selected structure similar components to investigate the influence of these functionalities within the linker of the conjugates on the clearance and steady-state volume of distribution of these conjugates *in vivo*. In Scheme 2, reductive amination between ZnDPA **11** and biphenyl-4-carboxaldehyde was first carried out to prepare intermediate **12**, which was later condensed with *endo*-bicyclo [6.1.0]nonyne-*para*-nitrophenol (*endo*-BCN-OpNP) to afford intermediate **16** or with *exo*-BCN-OpNP to afford intermediate **17** in moderate yields. Concurrently, Ugi four-component reaction (U-4CR) was performed to drive the linker diversification that includes an amino containing ZnDPA targeting ligand **11**, different isocyanides (*tert*-butyl isocyanide and diethyl isocyanomethylphosphonate), different acids (crotonic acid, 4′-Hydroxy-4-biphenylcarboxylic acid, *exo*-BCN butanoic acid **13**, and *exo*-BCN piperidine-4-carboxylic acid **14)**, and ketones (tetrahydro-4H-pyran-4-one and *exo*-BCN piperidi-4-one **15**). In particular, symmetrical cycloketone reactive component was selected to harness *exo*-BCN moiety, which should present a reactive site for azido-linker SN-38 intermediate **10** for SPAAC. In methanol at room temperature, U-4CR reactions were carried out among the components described in Scheme 2 and proceeded smoothly with yields ranging from 30% to 65% to furnish intermediates **18**–**21**. With the obtained structure–property relationship information, it should render support that multicomponent Ugi reaction could be employed as a facile linker diversification strategy for SMDC design.

In Scheme 3, the corresponding *endo-* or *exo-*BCN intermediates **16**–**21** were first allowed to react with aforementioned azido linker-SN-38 precursors **9** or **10** followed by incubation with two equivalents of zinc nitrate for the eventual preparations of ZnDPA-based conjugates **1**–**8** described in Synthetic procedures for these conjugates along with analytical, characterization data and spectra for key intermediates and conjugates were included (Supplementary Figures S1–S50).

**SCHEME 3:**
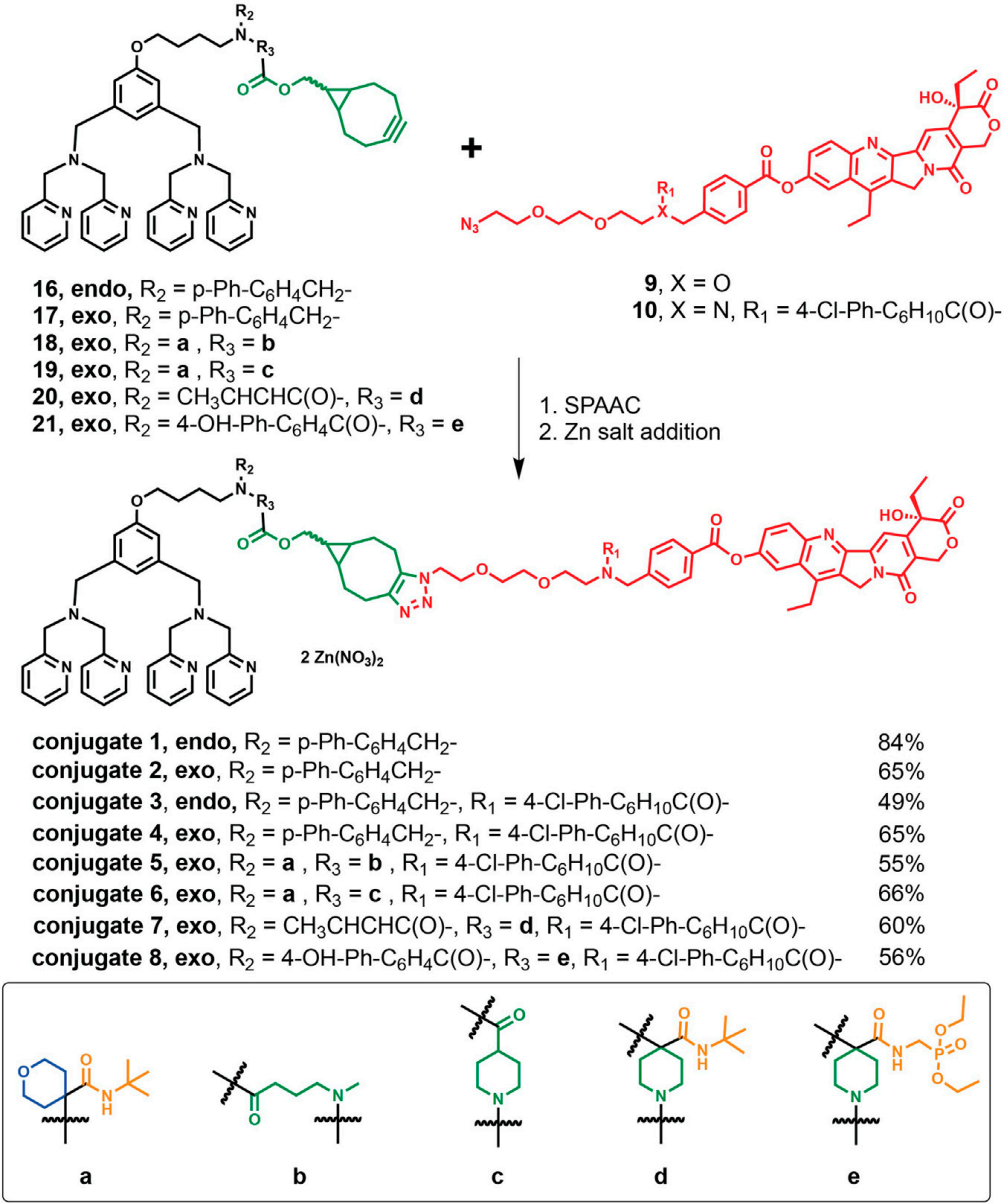
Synthetic routes of the target conjugates **1–8** though SPAAC and their yields followed by Zn addition.

As the premature release of the cytotoxic payload limits sufficient drug delivery and increases potential toxicities with off-target biodistribution, we first examined the plasma stabilities among the synthesized conjugates **1–8** by monitoring the ratio between the intact conjugates and the released payload SN-38 with an HPLC assay (Table 1). After incubation in mouse plasma for 6 h, conjugates **1** (82%) and **2** (85%) showed that less intact conjugate remained in comparison to those of **3** (90%) and **4** (96%), indicating that the cyclohexyl-para-chlorophenyl periphery adjacent to SN-38 played an important role to maintain stabilities of conjugates **3** and **4**. This conformational increase of steric hindrance in the linker region provided good plasma stabilities and could modulate the release of SN-38 by the esterase/protease-mediated cleavage in the tumor microenvironment (Liu et al., 2019). Interestingly, we also observed that conjugates **2** and **4** of *exo*-adduct exhibited improved plasma stabilities by 3%–6% over the corresponding *endo*-adducts in conjugates **1** and **3**. Our results suggested that the linker-drug portion in conjugate **4** provided favorable plasma stabilities over conjugates **1**–**3** and was then adopted for the synthesis of conjugates **5**–**8**. As such, conjugates **5–8** with appendages derived from multicomponent Ugi reactions were synthesized to further diversify the linker options, and we have observed similar plasma stability profiles to that of conjugate **4** with the installment of the same linker-drug portion. Our plasma stability data suggested that these ZnDPA-based conjugates should allow the masking of SN-38’s bioactivity during systemic circulation in a similar manner to CPT-11.

**TABLE 1:** ***In vitro* plasma stabilities**. Ratio of intact conjugates **1–8** and CPT-11 in comparison to SN-38 after 6 h incubation with mouse plasma. Data of CPT11 were adopted from a previous report (Liu et al., 2017).

Conjugates	Plasma stability (intact conjugate: SN-38) at 6 h
1	82:18
2	85:15
3	90:10
4	96:4
5	96:4
6	96:4
7	98:2
8	98:2
CPT-11	99:1

Similar to the clinical drug CPT-11, we reasoned that conjugates **4–8** would possess properties of prodrugs *via* modification of the C10-phenolic hydroxyl group in SN-38. Cytotoxicities of conjugates **4–8** were then assayed against MiaPaca-2 pancreatic cancer cells and Detroit 551 normal skin fibroblast cells. With a similar trend to that of CPT-11 (∼350 fold), conjugates **4–8** exhibited 10- to 35-fold decreases in potencies towards MiaPaca-2. There were modest 3-fold to >6-fold decreases in cytotoxicities towards normal fibroblast Detroit 551 as compared to that of cytotoxic SN-38. In particular, conjugate **4**, with IC_50_ ratio at 34-fold in MiaPaca-2 and >20 μM against Detroit 551, exhibited a similar prodrug trend that follows CPT-11. Taken together, we showed “clickable” linker types that conferred plasma stabilities with prodrug-like properties.

Next, to address the *in vivo* longevities and stabilities of U-4CR and/or “clicked” conjugates, *in vivo* systemic pharmacokinetic studies of conjugates **4–8** were carried out in the ICR mice. One single intravenous dose of 5 mg/kg of each conjugate was given to the mice. Plasma concentrations between the intact conjugate and the active payload (released SN-38 from the conjugate) in the circulation were measured and the pharmacokinetic parameters including clearance (CL), steady-state volume of distribution (V_ss_), and area under the plasma concentration curve (AUC_0–24h_) were determined (Table 2). Since SN-38 was the cytotoxic payload, the AUC ratio between the intact conjugate and SN-38 reflected the plasma stability, as these conjugates should be capable of uncaging the payload with higher protease activities in the tumor microenvironment. We envisioned that U-4CR could be a valuable synthetic strategy that unifies several advantageous features for bioactive SMDC *in vivo*. In addition to installing hydrophilic moiety to increase solubility and functional groups that modulate steady-state volume of distribution of the conjugate *in vivo*, a facile reactive handle for payload conjugation was also included. Indeed, we observed structure–property trends among the U-4CR-derived conjugates **5–8**. Since these conjugates harnessed the same *exo*-BCN clicked SN-38 portion, the U-4CR products between the Zn-DPA targeting ligand and other components might contribute factors that could affect their *in vivo* stabilities (Table 2). Different *exo*-BCN components, **13** for conjugate **5** and **14** for conjugate **6**, were employed and our results showed that piperidine-containing carbamate in conjugate **6** (AUC ratio of 7:1) might offer better *in vivo* stabilities over the methylated-carbamate from conjugate **5** (AUC ratio of 2.3:1). For conjugate **7**, we employed another *exo*-BCN component **15** and observed increases in the *in vivo* stability (AUC ratio of 16:1), suggesting that steric effect by introducing *gem*-dimethyl-like piperidine-containing carbamate could increase the systemic circulation exposure with the reduction of clearance rate (CL = 8.3 ml/min/kg). Among the U-4CR-derived conjugates, we observed that conjugate **8** harnessed the lowest steady-state volume of distribution (V_ss_ = 0.1 L/kg), suggesting that the addition of hydrophilic components with isocyanomethylphosphonate and hydroxy-biphenyl in the U-4CR led to favorable retention of circulation distribution.

**TABLE 2:** Pharmacokinetic profiles of conjugates **4–8** in male ICR mice (*n* = 3) at 5 mg/kg with intravenous administration.

Pharmacokinetic studies of conjugates in male ICR mice					
Conjugates	CL^a^	V_ss_ ^b^	AUC_conjugate_ ^c^	AUC_SN-38_ ^c^	AUC ratio
4	1.2	0.3	85,487	1,425	60:1
5	50.2	18.5	1,593	696	2.3:1
6	13.5	4.1	6,459	927	7.0:1
7	8.3	3.6	20,651	1,309	15.8:1
8	1.3	0.1	61,963	1,549	40:1

I.V., at 5 mg/kg (*n* = 3). Units.

a (ml/min/kg).

b (L/kg).

c (0–24 h: ng/ml*h).

With a 60-fold and 40-fold difference in the AUC for the intact conjugate **4** and **8** versus the released SN-38 within 24 h, respectively, these two conjugates exhibited better *in vivo* stabilities among the conjugates (Table 2). However, acute toxicity is another important parameter to be monitored in the development of SMDC. We observed that the lack of appreciable conjugate **4**’s acute toxicity was supported by the maximum tolerated dose (MTD) of a 5-day repeat dose of 50 mpk in ICR mice with a 1-week recovery monitoring period; while conjugate **8**’s MTD was determined to be 30 mpk under similar regimen. Hence, herein, we have selected ZnDPA-based conjugate **4** to demonstrate its efficacy and assessed that the described convergent synthetic strategy could afford bioactive conjugates. Although conjugate **8** did not show comparable MTD profile to that of conjugate **4**, our experimental results indeed showed that mix-and-matching different components of the U-4CR tandem with “click reaction” could lead to different pharmacokinetic profiles among structural similar conjugates. Not limited by the components described within, the optimization of *in vivo* pharmacokinetic profiles of alternative ZnDPA-based SMDC, we anticipate that U-4CR with combinations of different components can lead to the construction of improved properties, such as decrease in the clearance rate and steady-state volume of distribution (V_ss_). Moreover, we envision employment of an imaging agent as one of the components can also furnish functional theranostic if desired. In addition, to examine conjugate **4**’s association property towards PS after the linker-drug modification, we utilized a reported assay and conducted *in vitro* surface plasmon resonance (SPR) binding studies between conjugate **4** and a DOPS-coated lipid bilayer (Supplementary Figure S51) (Liu et al., 2017). Our previous results demonstrated that linker-drug or SN-38 itself did not result in direct association with PS, but rather the ZnDPA moiety contributed to the binding of PS (Liu et al., 2017). We showed that linker-drug modifications in conjugate **4** did not modulate the recognition between its ZnDPA targeting moiety towards PS (Supplementary Figure S51). Taken together, we identified conjugate **4** with improved pharmacokinetic profiles, increased MTD, and retained pro-drug properties among the designed clickable ZnDPA-based SN-38 conjugates.

To demonstrate the *in vivo* bioactivity of the “clicked” conjugate **4**, we then evaluated the therapeutic efficacy of conjugate **4** against mice bearing MiaPaca-2 human pancreatic tumor. We observed potent tumor growth inhibition effects with intravenous administration of conjugate **4** at 10 mpk with the qd (5 + 5) dosing schedule, which is 1/5 of the MTD amount weekly. During the course of treatment, conjugate **4** exhibited significant and prolonged tumor growth inhibition compared to that of CPT-11 without apparent body weight loss. Furthermore, tumor shrinkage, not observed in the CPT-11 treatment group, was also observed in the treatment group with conjugate **4**. These data provided evidence for the clicked conjugate **4** harnessed *in vivo* bioactivity and we found that conjugate **4** only deployed a total of 18 mg of SN-38, yet it achieved better anti-tumor efficacy than the CPT-11 treatment group that deployed 93 mg. Since conjugate **4** and CPT-11 were targeting conjugates or prodrugs of the anti-cancer payload SN-38, they consisted of 18% and 58% SN-38 by molecular weights, respectively. Therefore, conjugate **4** (10 mg/kg, 5 doses per week for 2 weeks) was given in a total dose of SN-38 at 18 mg/kg (i.e., 10 mg/kg/dose × 10 doses × 18% = 18 mg/kg). Similarly, CPT-11 at 40 mg/kg, 4 doses over 2 weeks (40 mg/kg × 4 doses × 58%), was equal to total doses of SN-38 at 93 mg/kg. Comparing the cargo usage between conjugate **4** and CPT-11, we observed a markedly 80% reduction of cytotoxic payload usage. Thus, this new clickable conjugate provided a useful platform for SN-38 delivery and also augment the therapeutic index of SN-38 by significantly reducing its payload usage as compared to that of the marketed CPT-11.

## Conclusion

By using click chemistry and/or Ugi multicomponent reaction, we have successfully synthesized several chemically distinct ZnDPA conjugates. Using the “clickable” convergent synthetic strategy, optimization of different parts of the whole SMDC can be carried out independently prior to the assembly. We have shown that the linker-SN-38 stability was first profiled in plasma *in vitro*, suggesting a different drug with an alternative linker could be caged first and assayed for the release mechanism. Concurrently, we leveraged Ugi multicomponent reaction to unify the ZnDPA targeting ligand, hydrophobic/hydrophilic moieties, and SPAAC reactive site to furnish the eventual conjugates. Our data also demonstrated the importance of linker’s modifications that affected the biodistribution and stabilities of the conjugates *in vivo*. Moreover, we envision that this tandem reaction sequence should allow one-pot synthesis of ensemble conjugates in helping the identification of new bioactive conjugates. For the realization of theranostic, not only the fluorescence probes could be installed as one of the components in the Ugi reaction, an alternative drug could also be introduced for the investigation of the synergistic effect between the two drugs in this ZnDPA-based drug conjugate. Inspired by the growing lines of evidence of PS in tumor progression and upon harnessing the ZnDPA-targeting ability of PS, this work demonstrated a facile way to expand the repertoire for ZnDPA-based drug conjugates’ design and constructions of diversified linkers. In addition, our studies underscored the important parameters, including plasma stability profiles, *in vitro* cytotoxicities, *in vivo* pharmacokinetics, and MTD properties, for the design of such conjugates. Our work highlighted the “clicked” conjugate **4** as a new bioactive SMDC to exhibit potent anti-pancreatic tumor efficacy. Taken together, chemical strategies reported herein could be utilized in concert or independently to incorporate a wide array of therapeutic agents to ZnDPA for the treatment of cell death-associated diseases.

## Data Availability

The original contributions presented in the study are included in the article/Supplementary Material, further inquiries can be directed to the corresponding author.
