# Supplementary material for: Click Chemistry and Multicomponent Reaction for Linker Diversification of Zinc Dipicolylamine-Based Drug Conjugates
Source: Front Chem. 2022 Feb 15;9:822587. doi: 10.3389/fchem.2021.822587 (PMC8886374; doi:10.3389/fchem.2021.822587)
Supplement: Supplementary file 1 [file DataSheet1.PDF]

Supplementary Material

table of contents

Synthesis of compounds 2
Spectra of precursor of conjugate 4. 20
Spectra of compound 9. 23
Spectra of compound 10. 26
Spectra of compound 12. 29
Spectra of compound 16. 32
Spectra of compound 17. 35
Spectra of compound 18. 38
Spectra of compound 19. 41
Spectra of compound 20. 44
Spectra of compound 21. 47
Spectra of precursor of conjugate 1. 49
Spectra of precursor of conjugate 2. 52
Spectra of precursor of conjugate 3. 55
Spectra of precursor of conjugate 5. 58
Spectra of precursor of conjugate 6. 61
Spectra of precursor of conjugate 7. 64
Spectra of precursor of conjugate 8. 67

Figure S51 70

Synthesis of compounds

2-[2-(2-azidoethoxy)ethoxy]ethanol (2)

To a solution of 2-[2-(2-Chloroethoxy)ethoxy]ethanol (5.0 g, 29.65 mmol) and sodium azide (5.79 g, 88.95 mmol) in H_2O, and heat to reflux for overnight, and them extracted with DCM (50 mL x 3). The organic layers were dried over Na_2SO_4, filtered off and concentrated to yield the compound **2** (4.32 g, 83%). ^1H NMR (400 MHz, $CDCl_3$) δ 3.79 – 3.74 (t, *J* = 4.0, 2H), 3.74 – 3.68 (m, 6H), 3.68 – 3.61 (m, 2H), 3.45 – 3.37 (t, *J* = 4.0, 2H). ^{13}C NMR (101 MHz, $CDCl_3$) δ 72.57, 70.53, 70.35, 70.01, 61.67, 50.63. ESI-MS, calcd for $(C_6H_{13}N_3NaO_3)^+$:198.09, found 198.10 $(M + Na)^+$.

methyl 4-({2-[2-(2-azidoethoxy)ethoxy]ethoxy}methyl)benzoate (3) and 44-({2-[2-(2-azidoethoxy)ethoxy]ethoxy}methyl)benzoic acid (4)

To a solution of NaH (60%, 1.09 g, 27.26 mmol) in THF (40 mL), the compound **2** (2.39 g, 13.63 mmol) was added at 0°C and stirred for 10 mins. The methyl 4-(bromomethyl)benzoate (3.75 g, 16.36 mmol)was dissolved in THF (10 ml), and added into mixture solution at 0°C. The reaction solution was slowly warmed to room temperature and stirred for 1 hr, then saturated NH_4Cl(aq.) was poured into the reaction mixture, and extracted with EA (50 mL x 2). The combined organic layers were dried over Na_2SO_4, filtered off and concentrated in *vacuo*. The residue was purified by flash column chromatography with 50%EA in Hex to yield the compound **3** (3.41 g, 77%). ^1H NMR (400 MHz, $cdcl_3$) δ 8.09 (d, *J* = 8.1 Hz, 2H), 7.46 (d, *J* = 8.1 Hz, 2H), 4.66 (s, 2H), 3.79 – 3.66 (m, 10H), 3.39 (t, *J* = 5.0 Hz, 2H).

To a solution of compound **3** (3.41 g, 10.56 mmol) in DCM (95 mL) at room temperature. The NaOH (3.38 g, 84.47 mmol) dissolved in MeOH (10 mL) followed by adding into ester solution. The reaction solution was stirred for 2 hr, then H_2O (100 ml) was poured into the reaction mixture. The mixture

solution adding 2*N* HCl(aq.) switch pH to 2-5, and extracted with DCM (100 mL x 2). The organic layers were dried over Na_2SO_4, filtered off and concentrated to obtain the compound **4** (3.15 g, 96%).

^{1}H NMR (400 MHz, $CDCl_3$) δ 8.08 (d, *J* = 8.2 Hz, 2H), 7.45 (d, *J* = 8.2 Hz, 2H), 3.85 – 3.61 (m, 10H), 3.54 – 3.31 (d, *J* = 2 Hz, 2H). ^{13}C NMR (101 MHz, $CDCl_3$) δ 171.55, 144.64, 130.48, 128.82, 127.43, 72.76, 70.89, 70.24, 70.06, 50.85. ESI-MS, calcd for $(C_{14}H_{19}N_3NaO_5)^+$: 332.12, found 332.10 $(M + Na)^+$.

[2-(2-azidoethoxy)ethoxy]acetaldehyde (5)

To a solution of compound **2** (0.5 g, 2.85 mmol), IBX (2.4 g, 8.56 mmol) in EA, and stirred at 90°C for overnight. The mixture was filtered off and concentrated. The residue was purified by flash column chromatography with 50% EA in Hex to yield the compound **5** (0.23 g, 46%). ^{1}H NMR (400 MHz, $CDCl_3$) δ 9.73 (s, 2H), 4.19 (s, 1H), 3.76 – 3.65 (m, 8H), 3.46 – 3.38 (m, 3H). ^{13}C NMR (101 MHz, $CDCl_3$) δ 200.79, 72.61, 71.36, 70.28, 61.89, 50.81. ESI-MS, calcd for $C_6H_{11}N_3O_3$: 173.08, found 173.10 (M).

methyl 4-[({2-[2-(2-azidoethoxy)ethoxy]ethyl}amino)methyl]benzoate (6)

To a solution of compound **5** (0.19 g, 1.08 mmol), methyl 4-aminobenzoate (0.33 mg, 2.16 mmol) in DCM, and stirred at room temperature for 3 hr. The $NaB(OAc)_3H$ (0.11 g, 5.4 mmol) was added after 3 hr, and stirred at 50°C for another 3 hr. The mixture was extract with $NH_4Cl_{(aq)}$ (50 mL), the organic layer was dried over $MgSO_4$, concentrated under reduced pressure. The residue was purified by flash column chromatography with 30% EA in Hex to yield the compound **6** (0.19 g, 58%). ^{1}H NMR (400 MHz, $CDCl_3$) δ 8.01 (d, *J* = 8 Hz, 2H), 7.43 (d, *J* = 8 Hz, 2H), 3.93 (s, 3H), 3.89 (s, 2H), 3.77 – 3.61 (m, 8H), 3.47 – 3.37 (d, *J* = 8 Hz, 2H), 2.90 – 2.78 (d, *J* = 8 Hz, 2H). ^{13}C NMR (101 MHz, $CDCl_3$) δ 167.10, 145.62, 129.77, 128.87, 128.09, 70.65, 70.55, 70.40, 70.11, 53.48, 52.10, 50.69, 48.72. ESI-MS, calcd for $(C_{15}H_{23}N_4O_4)^+$:323.17, found 323.20 $(M + H)^+$.

methyl 4-[({2-[2-(2-azidoethoxy)ethoxy]ethyl}{[1-(4-chlorophenyl)cyclohexyl]carbonyl}amino) methyl]benzoate (7) and 4-[({2-[2-(2-azidoethoxy)ethoxy]ethyl}{[1-(4-chlorophenyl)cyclohexyl] carbonyl}amino)methyl]benzoic acid (8)

To a solution of 1-(4-chlorophenyl)cyclohexane-1-carboxylic acid (1.07 g, 4.48 mmol) in DCM (18 ml) and $DMF_{(cat.)}$ was added oxalyl chloride (0.58 g, 4.48 mmol) at 0°C. The mixture solution was stirred at room temperature for 1 hr, and then a solution of **6** (0.69 g, 2.23 mmol) in DCM, and *N,N*-diisopropyl-ethylamine (DIPEA) (2.32 g, 7.93 mmol) were added at 0°C, and stirred at room temperature for another 4 hr. The mixture was extract with cold $NH_4Cl_{(aq)}$ (50 mL), the organic layer was dried over $MgSO_4$, concentrated under reduced pressure. The residue was purified by flash column chromatography with 40% EA in Hex to yield the compound **7** (1.12 g, 95%). ^{1}H NMR (400 MHz, $cdcl_3$) δ 7.85 (d, J = 8.0 Hz, 2H), 7.45 – 7.27 (m, 4H), 6.57 (d, J = 8.1 Hz, 2H), 5.31 (m, 2H), 3.84 (d, J = 0.9 Hz, 3H), 3.74 – 3.64 (m, 8H), 3.37 (m, 4H), 2.32 – 2.19 (m, 2H), 1.99 – 1.88 (m, 2H), 1.69 – 1.55 (m, 5H), 1.42 – 1.33 (m, 1H). To a stirred solution of **7** (0.41 g, 0.77 mmol) was dissolved in THF (7 ml), the 0.5*N* LiOH (14 ml) was added at 0°C, and stirred for 4 hr. THF was removed after reaction complete, the residue was extracted with DCM (50 mL x 2) and $NH_4Cl_{(aq)}$ (20 mL). The organic layers were dried over $MgSO_4$, concentrated under reduced pressure to yield compound **8** (0.24 g, 60%). ^{1}H NMR (400 MHz, $CDCl_3$) δ 8.01 (d, J = 7.6 Hz, 2H), 7.32 – 7.21 (m, 5H), 7.05 (s, 1H), 4.67 (s, 1H), 4.30 (s, 1H), 3.70 (s, 1H), 3.65 – 3.46 (m, 6H), 3.44 (s, 1H), 3.40 – 3.3 (m, 3H), 3.13 (s, 1H), 2.25 (m, 2H), 1.72 (m, 7H), 1.26 (m, 1H). ^{13}C NMR (101 MHz, $CDCl_3$) δ 175.26, 171.18, 144.65, 143.68, 132.49, 130.61, 129.22, 127.79, 126.92, 126.56, 70.73, 70.54, 70.17, 69.20, 52.76, 51.24, 50.82, 46.71, 37.05, 26.02, 23.82. ESI-MS, calcd for $(C_{27}H_{34}ClN_4O_5)^+$: 529.22, found 529.20 $(M + H)^+$.

(4*S*)-4,11-diethyl-4-hydroxy-3,14-dioxo-3,4,12,14-tetrahydro-1*H*-pyrano[3',4':6,7]indolizino[1,2-*b*]quinolin-9-yl 4-({2-[2-(2-azidoethoxy)ethoxy]ethoxy}methyl)benzoate (9)

To a stirred solution of acid **4** (30 mg, 0.097 mmol) was dissolved in toluene (2 ml), the $SOCl_2$ (34 ml, 0.466 mmol) and DMF (10 ml) was added at 0ºC. The solution was stirred at room temperature. After 3 hr, the solution of (4S)-4,11-diethyl-4,9-dihydroxy-1*H*-pyrano[3',4':6,7]indolizino[1,2-*b*] quinoline-3,14 (4*H*,12*H*)-dione (SN-38, 32 mg, 0.081 mmol) in dimethylacetamide (DMA, 2 ml) and DIPEA (40 ul, 0.243 mmol) were added into mixture solution. The reaction was completion after one day, the mixture was extract with $NaHCO_3$ (50 mL) and DCM (90 ml), organic layer was washed with saturated aqueous $NH_4Cl_{(aq)}$ (40 ml). The combined organic layers were dried over $MgSO_4$, concentrated under reduced pressure. The resulting residues were purified by flash column chromatography with 5 % MeOH in DCM to yield compound **9** (38 mg, 69%). ^{1}H NMR (400 MHz, $cdcl_3$) δ 8.32-8.21 (m, 3H), 7.97 (s, 1H), 7.75-7.63 (m, 2H), 7.55 (d, *J* = 8.1 Hz, 2H), 5.76 (d, *J* = 16.3 Hz, 1H), 5.35-5.26 (m, 3H), 4.72-4.57 (m, 2H), 3.78-3.66 (m, 10H), 3.44-3.35 (m, 2H), 3.18 (q, *J* = 7.6 Hz, 2H), 1.98-1.85 (m, 2H), 1.42 (t, *J* = 7.7 Hz, 3H), 1.05 (t, *J* = 7.4 Hz, 3H). ^{13}C NMR (101 MHz, $cdcl_3$) δ 173.71, 164.88, 157.57, 151.76, 150.24, 149.86, 147.31, 146.70, 145.34, 145.02, 144.87, 132.04, 130.37, 128.10, 127.40, 127.29, 125.49, 118.63, 114.73, 98.12, 73.90, 72.82, 72.49, 70.72, 70.06, 70.02, 66.21, 58.54, 50.67, 49.39, 31.62, 23.15, 13.99, 7.84. ESI-HRMS, calcd for $(C_{36}H_{36}N_5O_9)^-$: 682.2513, found 682.2509 $(M - H)^-$.

4*S*)-4,11-diethyl-4-hydroxy-3,14-dioxo-3,4,12,14-tetrahydro-1*H*-pyrano[3',4':6,7]indolizino[1,2-*b*]quinolin-9-yl 4-[({2-[2-(2-azidoethoxy)ethoxy]ethyl}{[1-(4-chlorophenyl)cyclohexyl]carbonyl} amino)methyl]benzoate (10)

To a stirred solution of acid **8** (0.56 g, 1.064 mmol) was dissolved in DMF (5 ml), and hydroxybenzotriazole (HOBt, 0.22 g, 1.596 mmol) and DIPEA (0.28 g, 2.128 mmol) were added. The mixture was stirred at room temperature for 0.5 hr, and then SN-38 (0.46 g, 1.171 mmol) was added and stirred for another 0.5 hr. After completion of reaction, the mixture was extract with DCM (300 ml) and

saturated aqueous $NH_4Cl_{(aq)}$ (300 ml). The combined organic layers were dried over $MgSO_4$, concentrated under reduced pressure. The resulting residues were purified by flash column chromatography with 20 % EA in DCM to yield compound **10** (0.58 g, 61%).

^{1}H NMR (400 MHz, cdcl$_3$) δ 8.30 (d, *J* = 9.1 Hz, 1H), 8.18 (d, *J* = 7.6 Hz, 2H), 7.96 (d, *J* = 2.2 Hz, 1H), 7.69 (d, *J* = 11.7 Hz, 2H), 7.34 – 7.22 (m, 6H), 7.13 (s, 1H), 5.77 (d, *J* = 16.3 Hz, 1H), 5.44 – 5.22 (m, 3H), 4.38 (s, 1H), 3.78 (s, 1H), 3.65 – 3.54 (m, 6H), 3.48 (m, 1H), 3.41 – 3.33 (m, 3H), 3.18 (m, 3H), 2.28 (m, 2H), 1.91 (m, 2H), 1.69 (m, 6H), 1.42 (t, *J* = 7.7 Hz, 3H), 1.05 (t, *J* = 7.4 Hz, 3H), 0.93 (m, 2H). ^{13}C NMR (101 MHz, cdcl$_3$) δ 175.02, 173.78, 164.81, 157.66, 150.33, 149.93, 147.42, 146.77, 145.42, 144.48, 132.38, 132.19, 130.59, 129.12, 127.52, 127.43, 126.89, 125.54, 118.77, 114.80, 98.29, 72.95, 71.79, 71.13, 70.64, 70.46, 70.07, 69.18, 66.28, 61.89, 52.67, 51.14, 50.73, 49.47, 46.70, 36.97, 31.71, 29.74, 25.92, 23.71, 23.23, 14.07, 7.93. ESI-HRMS, calcd for $(C_{49}H_{50}ClN_6O_9)^-$: 901.3328, found 901.3326 $(M - H)^-$.

N-(biphenyl-4-ylmethyl)-4-(3,5-bis{[bis(pyridin-2-ylmethyl)amino]methyl}phenoxy)butan-1-amine (12)

To a solution of **11** (5.41 g, 9.21 mmol) and biphenyl-4-carboxaldehyde (1.40 g, 7.67 mmol) in DCM (50 mL) was added sodium triacetoxyborohydride (2.12g, 9.97 mmol) at room temperature and stirred for overnight. Then extracted with saturated $NaHCO_{3(aq)}$. The organic layers were dried over Na_2SO_4, filtered off and concentrated in *vacuo*. The residue was purified by chromatography with 5% MeOH in DCM to yield the **12** (5.39 g, 78%). ^{1}H NMR (600 MHz, $CDCl_3$) δ 8.52 (m, 4H), 7.65 – 7.54 (m, 12H), 7.35 (t, *J* = 7.2 Hz, 1H), 7.13 (t, *J* = 6.0 Hz, 4H), 7.08 (s, 1H), 6.87 (s, 2H), 3.99 (t, *J* = 6.4 Hz, 2H), 3.86 (s, 2H), 3.82 (s, 8H), 3.67 (s, 4H), 2.79 – 2.73 (m, 2H), 1.91 – 1.83 (m, 2H), 1.78 – 1.71 (m, 2H). ^{13}C NMR (151 MHz, $CDCl_3$) δ 159.8, 159.2, 149.0, 141.0, 140.6, 139.9, 139.5, 136.4, 128.8, 128.6, 127.2, 127.0, 122.7, 121.9, 121.4, 113.5, 67.7, 60.1, 58.6, 53.7, 53.4, 49.2, 27.2, 26.8. ESI-HRMS, calcd for $(C_{49}H_{52}N_7O)^+$:754.4233, found 754.4231 $(M + H)^+$.

4-[{[(1*R*,8*S*,9*r*)-bicyclo[6.1.0]non-4-yn-9-ylmethoxy]carbonyl}(methyl)amino]butanoic acid (13)

To a solution of 4-(Methylamino)butyric acid hydrochloride (22 mg, 0.145 mmol) in H_2O (1 ml) was added Na_2CO_3 (51 mg, 0.477 mmol). (1R,8S,9r)-Bicyclo[6.1.0]non-4-yn-9-ylmethyl 4-nitrophenyl carbonate (*exo*-BCN-O*p*NP) (50 mg, 0.159 mmol) was dissolved in THF (3 ml), and poured into the solution mixture at room temperature and stirred for 10 hr, then saturated $NH_4Cl_{(aq.)}$ was poured into the reaction mixture. The mixture solution was extracted with DCM (100 mL). The organic layers were dried over Na_2SO_4, filtered off and concentrated in *vacuo*. The residue was purified by flash column chromatography with 5% MeOH in DCM to yield the compound **13** (33 mg, 72 %). ^{1}H NMR (400 MHz, $CDCl_3$) δ 4.00 (d, *J* = 6.6 Hz, 2H), 3.33 (m, 2H), 2.91 (s, 3H), 2.39 (m, 4H), 2.28 (d, *J* = 13.6 Hz, 2H), 2.19 – 2.11 (m, 2H), 1.88 (m, 2H), 1.42 – 1.36 (m, 2H), 0.72 (m, 3H). ^{13}C NMR (101 MHz, $CDCl_3$) δ 178.52, 157.19, 98.97, 69.86, 48.06, 33.48, 29.87, 23.96, 23.02, 22.86, 21.55, 14.30. ESI-HRMS, calcd for $(C_{16}H_{23}NNaO_4)^+$: 316.1525, found 316.1527 $(M + Na)^+$.

1-{[(1*R*,8*S*,9*r*)-bicyclo[6.1.0]non-4-yn-9-ylmethoxy]carbonyl}piperidine-4-carboxylic acid (14)

A mixture of isonipecotic acid (26 mg, 0.20 mmol), *exo*-BCN-O*p*NP (70 mg, 0.22 mmol), 4-Dimethylaminopyridine (DMAP) (2.5 mg, 0.02 mmol) and DIPEA (150 μl g, 0.9 mmol) in DMF (0.5 mL) and DCM (1.5 mL) was stirred at room temperature for 8 hr, then saturated $NH_4Cl_{(aq.)}$ was poured into the reaction mixture. The mixture solution was extracted with DCM (100 mL). The organic layers were dried over Na_2SO_4, filtered off and concentrated in *vacuo*. The residue was purified by flash column chromatography with 5% MeOH in DCM to yield the compound **14** (48 mg, 70 %). ^{1}H NMR (400 MHz, $CDCl_3$) δ 4.07 (s, 2H), 4.01 (d, *J* = 6.6 Hz, 2H), 2.93 (s, 2H), 2.56 – 2.47 (m, 1H), 2.40 (d, *J* = 13.3 Hz, 2H), 2.36 – 2.22 (m, 2H), 2.21 – 2.07 (m, 2H), 1.94 (m, 2H), 1.74 – 1.58 (m, 2H), 1.38 (m, 2H), 0.91 – 0.83 (m, 1H), 0.75 – 0.70 (m, 2H). ^{13}C NMR (101 MHz, $CDCl_3$) δ 179.45, 155.77, 98.99, 69.80, 43.29,

40.85, 33.53, 29.89, 27.91, 23.98, 23.02, 21.59. ESI-MS, calcd for $(C_{17}H_{24}NO_4)^+$: 306.17, found 306.25 $(M + H)^+$.

(1*R*,8*S*,9*r*)-bicyclo[6.1.0]non-4-yn-9-ylmethyl 4-oxopiperidine-1-carboxylate(15)

A mixture of 4-piperidone monohydrate hydrochloride (85 mg, 0.55 mmol), *exo*-BCN-O*p*NP (158 mg, 0.50 mmol), DMAP (61 mg, 0.50 mmol) and DIPEA (259 mg, 2.0 mmol) in MeCN (5 mL) was stirred at 80 °C for 2 hr. After MeCN was removed, the residue was diluted in DCM (50 mL) and extracted with 1M HCl (20 mL x 3), and then extracted with 1M NaOH (20 mL x 3). The organic layers were dried over Na_2SO_4, filtered off and concentrated in *vacuo*. The residue was purified by flash column chromatography with 5% MeOH in DCM to yield the compound **15** (104 mg, 76 %). ^1H NMR (400 MHz, $CDCl_3$) δ 4.07 (d, J = 8.0 Hz, z2H), 3.79 (t, J = 6.2 Hz, 4H), 2.47 (t, J = 6.2 Hz, 4H), 2.44 – 2.37 (m, 2H), 2.35 – 2.24 (m, 2H), 2.18 (s, 2H), 1.45 – 1.32 (m, 2H), 0.82 – 0.70 (m, 3H). ^{13}C NMR (101 MHz, $CDCl_3$) δ 207.52, 155.57, 98.87, 70.23, 43.22, 41.25, 33.44, 23.84, 23.04, 21.50. ESI-HRMS, calcd for $(C_{16}H_{21}NNaO_3)^+$: 298.1419, found 298.1415 $(M + Na)^+$.

(1*R*,8*S*,9*s*)-bicyclo[6.1.0]non-4-yn-9-ylmethyl (biphenyl-4-ylmethyl)[4-(3,5-bis{[bis(pyridin-2-ylmethyl)amino]methyl}phenoxy)butyl]carbamate (16)

A mixture of **12** (197 mg, 0.26 mmol), *endo*-BCN-O*p*NP (94 mg, 0.31 mmol), DMAP (4 mg, 0.229 mmol) and DIPEA (101 mg, 0.78 mmol) in DCM (2.6 mL) was stirred at 50°C for 2 hr. After completion of reaction, the mixture solution was concentrated in *vacuo*. The residue was purified by flash column chromatography with 100% MeOH to yield the compound **16** (0.18 g, 73%). ^1H NMR (400 MHz, $cdcl_3$) δ 8.50 (d, J = 4.7 Hz, 4H), 7.63 – 7.52 (m, 12H), 7.42 (t, J = 7.5 Hz, 2H), 7.37 – 7.28 (m, 3H), 7.12 (m, 5H), 6.83 (s, 2H), 4.55 (s, 2H), 4.23 (d, J = 7.4 Hz, 2H), 3.94 (s, 2H), 3.80 (s, 8H), 3.64 (s, 4H), 3.39 (m, 2H), 2.22 (m, 6H), 1.79 (m, 4H), 1.56 (m, 2H), 1.39 (m, 1H), 0.98 – 0.82 (m, 2H). ^{13}C NMR (101 MHz,

cdcl3) δ 159.77, 159.15, 156.71, 148.98, 140.61, 140.27, 136.43, , 113.50, 98.83, 67.44, 63.47, 60.06, 58.59, 49.98, 46.91, 46.05, 29.73, 29.10, 26.69, 21.45, 20.16, 17.83.

ESI-HRMS, calcd for $(C_{60}H_{64}N_7O_3)^+$: 930.5071, found 930.5099 $(M + H)^+$.

(1*R*,8*S*,9*r*)-bicyclo[6.1.0]non-4-yn-9-ylmethyl (biphenyl-4-ylmethyl)[4-(3,5-bis{[bis(pyridin-2-ylmethyl)amino]methyl}phenoxy)butyl]carbamate (17)

A mixture of **12** (143 mg, 0.19 mmol), *exo*-BCN-O*p*NP (66 mg, 0.209 mmol), DMAP (23.2 mg, 0.19 mmol) and DIPEA (49 mg, 0.38 mmol) in DMF (2 mL) was stirred at 80°C for 5 hr. After completion of reaction, the mixture solution was extracted with H_2O (50 mL x 2). and DCM (50 mL x 2). The organic layers were dried over Na_2SO_4, filtered off and concentrated in *vacuo*. The residue was purified by flash column chromatography with 6% MeOH in DCM with 2% Et_3N to yield the compound **17** (0.125 g, 71%). ^{1}H NMR (400 MHz, cdcl3) δ 8.50 (d, J = 4.9 Hz, 4H), 7.65 – 7.52 (m, 12H), 7.42 (t, J = 7.5 Hz, 2H), 7.36 – 7.28 (m, 3H), 7.15 – 7.04 (m, 5H), 6.82 (s, 2H), 4.53 (s, 2H), 4.11 – 3.89 (m, 4H), 3.80 (s, 8H), 3.64 (s, 4H), 3.38 (m, 2H), 2.41 – 2.04 (m, 6H), 1.87 (m, 2H), 1.74 (m, 4H), 1.34 (m, 1H), 0.71 (m, 2H). ^{13}C NMR (101 MHz, cdcl3) δ 159.76, 159.14, 148.96, 140.59, 140.26, 136.41, 128.81, 128.26, 127.63, 127.34, 127.24, 126.99, 122.75, 121.95, 121.46, 113.53, 98.77, 69.63, 67.46, 60.05, 58.58, 49.98, 46.90, 46.12, 33.30, 29.70, 26.70, 24.64, 23.91, 22.96, 21.40. ESI-HRMS, calcd for $(C_{60}H_{64}N_7O_3)^+$: 930.5071, found 930.5073 $(M + H)^+$.

General procedure A: synthesis of compounds 18-21 by Ugi reaction

A mixture of aniline **11** (0.3 mmol), carboxyl acid (0.4 mmol), isocyanide (0.4 mmol) and ketone (0.4 mmol) in MeOH (0.8 mL) was stirred at room temperature until no aniline **11** remained. After completion of reaction, MeOH was removed under reduced pressure. The mixture was purified by flash column chromatography with 6% MeOH in DCM with 2% Et_3N to afford product.

(1*R*,8*S*,9*r*)-bicyclo[6.1.0]non-4-yn-9-ylmethyl (4-{[4-(3,5-bis{[bis(pyridin-2-ylmethyl)amino] methyl}phenoxy)butyl][4-(*tert*-butylcarbamoyl)tetrahydro-2*H*-pyran-4-yl]amino}-4-oxobutyl)methylcarbamate (18)

Following procedure of *General procedure A*, compound **18** (186 mg, 52%) was prepared from **11** (200 mg), **13** (120 mg), tetrahydro-*4H*-pyran-4-one (41 mg), *tert*-butyl isocyanide (34 mg) as sticky oil. ^{1}H NMR (400 MHz, cdcl$_3$) δ 8.51 (d, *J* = 4.8 Hz, 4H), 7.66 – 7.56 (m, 8H), 7.13 (m, 4H), 6.82 (s, 3H), 3.96 (m, 4H), 3.80 (m, 12H), 3.66 (s, 4H), 3.39 (m, 2H), 3.27 (m, 2H), 2.88 (s, 3H), 2.50 (m, 2H), 2.37 (m, 4H), 2.25 (t, *J* = 13.8 Hz, 2H), 2.12 (d, *J* = 15.0 Hz, 2H), 1.93 (m, 4H), 1.88 – 1.82 (m, 2H), 1.31 (m, 13H), 0.81 – 0.62 (m, 3H). ^{13}C NMR (101 MHz, cdcl$_3$) δ 174.22, 172.08, 171.64, 159.62, 158.79, 156.60, 148.89, 140.54, 136.34, 122.67, 121.89, 121.58, 113.42, 98.66, 69.26, 66.98, 64.68, 63.32, 59.94, 58.45, 50.73, 48.10, 44.43, 34.07, 33.25, 32.07, 28.54, 27.69, 26.71, 23.77, 23.11, 22.73, 21.30. ESI-HRMS, calcd for $(C_{62}H_{78}N_9O_6)^-$: 1044.6075, found 1044.6073 $(M\text{-}H)^-$.

1*R*,8*S*,9*r*)-bicyclo[6.1.0]non-4-yn-9-ylmethyl 4-{[4-(3,5-bis{[bis(pyridin-2-ylmethyl)amino] methyl}phenoxy)butyl][4-(*tert*-butylcarbamoyl)tetrahydro-2*H*-pyran-4-yl]carbamoyl} piperidine-1-carboxylate (19)

Following procedure of *General procedure A*, compound **19** (123 mg, 28%) was prepared from **11** (247 mg), **14** (193 mg, 0.5 mmol), tetrahydro-*4H*-pyran-4-one (51 mg), *tert*-butyl isocyanide (42 mg) as sticky oil. ^{1}H NMR (400 MHz, cdcl$_3$) δ 8.51 (d, *J* = 4.8 Hz, 4H), 7.70 – 7.54 (m, 8H), 7.16 – 7.11 (m, 4H), 6.83 (s, 3H), 4.17 (s, H), 3.98 (s, 4H), 3.80 (s, 12H), 3.66 (s, 4H), 3.42 (s, 2H), 2.66 (m, 2H), 2.52 (m, 2H), 2.38 (d, *J* = 13.2 Hz, 2H), 2.26 (d, *J* = 13.6 Hz, 2H), 2.14 (d, *J* = 14.9 Hz, 2H), 1.94 (m, 2H), 1.71 (s, 6H), 1.29 (s, 11H), 0.76 – 0.61 (m, 3H). ^{13}C NMR (101 MHz, d$_2$o) δ 177.15, 171.68, 159.60, 158.74, 155.34, 148.93, 140.61, 136.39, 122.71, 121.95, 121.78, 113.41, 98.74, 69.46, 66.88, 64.69, 63.65, 59.91, 58.44, 50.76, 44.31, 43.19, 40.97, 34.12, 33.29, 28.79, 28.55, 26.60, 23.76, 22.78, 21.36. ESI-HRMS, calcd for $C_{63}H_{78}N_9O_6$:1056.6075, found 1056.6084 (M)

(1*R*,8*S*,9*r*)-bicyclo[6.1.0]non-4-yn-9-ylmethyl 4-{[4-(3,5-bis{[bis(pyridin-2-ylmethyl)amino]methyl} phenoxy)butyl][(2*E*)-but-2-enoyl]amino}-4-(*tert*-butylcarbamoyl)piperidine-1-carboxylate(20)

Following procedure of *General procedure A*, compound **20** (234 mg, 68%) was prepared from **11** (200 mg), **15** (113 mg), crotonic acid (35 mg), *tert*-butyl isocyanide (34 mg) as sticky oil. ^{1}H NMR (600 MHz, CDCl3) δ 8.57 – 8.47 (m, 4H), 7.67 – 7.54 (m, 8H), 7.18 – 7.08 (m, 5H), 6.93 – 6.75 (m, 3H), 6.21 (d, *J* = 14.9 1H), 3.94 (m, 4H), 3.80 (s, 8H), 3.65 (m, 6H), 3.59 – 3.29 (m, 4H), 2.48 – 2.32 (m, 4H), 2.31 – 2.23 (m, 2H), 2.14 (d, J = 14.9 Hz, 2H), 2.07 – 1.98 (m, 2H), 1.91 – 1.67 (m, 7H), 1.34 – 1.28 (m, 11H), 0.88 (t, *J* = 7.0 Hz, 1H), 0.75 – 0.65 (m, 2H). ^{13}C NMR (151 MHz, $CDCl_3$) δ 172.45, 170.21, 159.85, 159.08, 155.80, 149.12, 142.89, 140.80, 136.64, 124.48, 122.94, 122.17, 113.67, 98.97, 69.70, 67.23, 64.32, 60.15, 58.75, 51.04, 33.52, 33.04, 29.88, 28.75, 28.03, 26.89, 23.99, 23.02, 21.57, 18.33, 14.29. ESI-HRMS, calcd for $C_{61}H_{74}N_9O_5)^-$: 1012.5813, found 1012.5816 $(M - H)^-$.

(1*R*,8*S*,9*r*)-bicyclo[6.1.0]non-4-yn-9-ylmethyl 4-{[4-(3,5-bis{[bis(pyridin-2-ylmethyl)amino]methyl} phenoxy)butyl][(4'-hydroxybiphenyl-4-yl)carbonyl]amino}-4-{[(diethoxyphosphoryl)methyl] carbamoyl}piperidine-1-carboxylate (21)

Following procedure of *General procedure A*, compound **21** (151 mg, 48%) was prepared from **11** (150 mg), **15** (84 mg), 4'-Hydroxy-4-biphenylcarboxylic acid (65 mg), diethyl isocyanomethylphosphonate (54 mg) as sticky oil. ^{1}H NMR (400 MHz, $cdcl_3$) δ 8.51 (d, *J* = 4.8 Hz, 4H), 7.64 – 7.57 (m, 8H), 7.52 (d, *J* = 8.2 Hz, 2H), 7.46 (d, *J* = 8.3 Hz, 2H), 7.36 (d, *J* = 8.6 Hz, 2H), 7.20 – 7.12 (m, 5H), 6.86 (d, *J* = 8.5 Hz, 2H), 6.33 (s, 2H), 4.17 (m, *J* = 7.1 Hz, 4H), 4.01 (m, 2H), 3.77 (dd, *J* = 12.1, 5.7 Hz, 2H), 3.66 (m 14H), 3.46 (m, 2H), 3.33 – 3.26 (m, 4H), 2.40 (m, 2H), 2.28 (m, 4H), 2.16 (d, 2H), 1.66 (m, 6H), 1.34 (t, *J* = 7.1 Hz, 6H), 0.87 (d, 2H), 0.77 – 0.69 (m, 3H). ESI-HRMS, calcd for $(C_{71}H_{81}N_9O_9P)^-$: 1234.5895, found 1234.5902 $(M - H)^-$.

General procedure B: synthesize conjugates 1-8 by SPAAC reaction

A mixture of alkyne (1 eq), azido (1.1 eq) in DMF (0.5 mL) was stirred at room temperature for 2 hr. After 2 hr, DMF was removed under reduced pressure. The mixture was purified by flash column chromatography with 6% MeOH in DCM with 2% Et_3N to afford precursors of ***conjugates 1-8***.

(4*S*)-4,11-diethyl-4-hydroxy-3,14-dioxo-3,4,12,14-tetrahydro-1*H*-pyrano[3',4':6,7]indolizino[1,2-*b*]quinolin-9-yl 4-{[2-(2-{2-[(5a*R*,6*S*,6a*S*)-6-[({(biphenyl-4-ylmethyl)[4-(3,5-bis{[bis(pyridin-2-ylmethyl)amino]methyl}phenoxy)butyl]carbamoyl}oxy)methyl]-5,5a,6,6a,7,8-hexahydrocyclo-propa[5,6]cycloocta[1,2-*d*][1,2,3]triazol-1(4*H*)-yl]ethoxy}ethoxy)ethoxy]methyl}benzoate (precursor of conjugate 1)

Following *General procedure B,* precursor of conjugate **1** (sticky oil, 260 mg, 84%) was obtained via SPAAC reaction between intermediate **16** (175 mg) and **9** (143 mg). ^{1}H NMR (400 MHz, cdcl$_3$) δ 8.49 (d, *J* = 4.9 Hz, 4H), 8.29 (d, *J* = 9.1 Hz, 1H), 8.23 (d, *J* = 8.2 Hz, 2H), 7.97 (d, *J* = 2.4 Hz, 1H), 7.69 (m, 2H), 7.60 – 7.50 (m, 14H), 7.42 (m, 2H), 7.31 (m, 3H), 7.16 – 7.05 (m, 5H), 6.81 (s, 2H), 5.76 (d, *J* = 16.3 Hz, 1H), 5.35 – 5.26 (m, 3H), 4.67 (s, 2H), 4.54 (s, 2H), 4.36 (m, 2H), 4.19 (m, 1H), 3.99 (m, 1H), 3.93 (m, 2H), 3.85 (m, 2H), 3.79 (s, 8H), 3.67 – 3.56 (m, 12H), 3.17 (m, 2H), 3.16 (m, 2H), 3.07 (m, 1H), 2.88 (s, 2H), 2.67 (m, 1H), 2.11 (m, 2H), 1.95 – 1.73 (m, 6H) , 1.55 (m, 2H), 1.41 (t, *J* = 7.6 Hz, 3H), 1.25 (m, 1H), 1.03 (m, 5H). ^{13}C NMR (101 MHz, cdcl$_3$) δ 173.71, 164.90, 159.72, 159.11, 157.62, 151.90, 150.41, 149.89, 148.94, 147.40, 146.72, 145.35, 144.99, 144.37, 140.58, 140.23, 136.47, 134.06, 132.10, 130.42, 128.84, 128.29, 128.18, 127.63, 127.49, 127.43, 127.35, 127.23, 126.97, 125.53, 122.76, 121.99, 121.42, 118.73, 114.83, 113.54, 98.23, 72.87, 72.52, 70.67, 70.61, 70.18, 70.04, 67.44, 66.21, 63.39, 60.01, 58.60, 53.52, 50.41, 50.04, 49.42, 47.81, 31.69, 26.69, 25.93, 24.93, 23.18, 23.14, 22.78, 22.25, 20.03, 19.50, 17.80, 14.03, 7.92. ESI-HRMS, calcd for $C_{96}H_{100}N_{12}O_{12}$ 1612.7584, found 1612.7579 (M).

(4*S*)-4,11-diethyl-4-hydroxy-3,14-dioxo-3,4,12,14-tetrahydro-1*H*-pyrano[3',4':6,7]indolizino[1,2-*b*]quinolin-9-yl 4-{[2-(2-{2-[(5a*R*,6*R*,6a*S*)-6-[({(biphenyl-4-ylmethyl)[4-(3,5-bis{[bis(pyridin-2-ylmethyl)amino]methyl}phenoxy)butyl]carbamoyl}oxy)methyl]-5,5a,6,6a,7,8-hexahydro-cyclopropa[5,6]cycloocta[1,2-*d*][1,2,3]triazol-1(4*H*)-yl]ethoxy}ethoxy)ethoxy]methyl}benzoate (precursor of conjugate 2)

Following *General procedure B,* precursor of conjugate **2** (sticky oil, 407 mg, 65%) was obtained via SPAAC reaction between intermediate **17** (263 mg) and **9** (213 mg). ^{1}H NMR (400 MHz, cdcl$_3$) δ 8.54 – 8.47 (m, 4H), 8.29 (d, *J* = 9.2 Hz, 1H), 8.23 (d, *J* = 8.3 Hz, 2H), 7.97 s, 1H), 7.71 – 7.66 (m, 2H), 7.61 – 7.50 (m, 14H), 7.41 (m, 2H), 7.33 (m, 3H), 7.11 (m, 5H), 6.82 (s, 2H), 5.75 (d, *J* = 16.3 Hz, 1H), 5.35 – 5.25 (m, 3H), 4.67 (s, 2H), 4.52 (s, 2H), 4.36 (s, 2H), 4.06 (m, 1H) 3.93 (s, 2H), 3.84 (t, *J* = 5.5 Hz, 2H), 3.79 (s, 8H), 3.68 – 3.62 (m, 8H), 3.58 (m, 4H), 3.39 (m, 2H), 3.17 (q, *J* = 7.5 Hz, 2H), 3.06 (m, 1H), 2.89 (m, 1H), 2.77 (m, 1H), 2.61 (m, 1H), 2.33 (m, 1H), 1.91 (m, 4H), 1.78 (m, 4H), 1.41 (t, *J* = 7.7 Hz, 3H), 1.30 (m, 2H), 1.05 (t, *J* = 7.4 Hz, 3H), 0.91 – 0.75 (m, 3H). ^{13}C NMR (101 MHz, cdcl$_3$) δ 173.53, 164.78, 159.67, 159.04, 157.50, 151.80, 150.34, 149.79, 148.86, 147.27, 146.57, 145.22, 144.92, 144.62, 140.52, 140.10, 136.36, 134.23, 131.99, 130.32, 128.75, 128.10, 127.58, 127.37, 127.33, 127.29, 127.11, 126.87, 125.40, 122.67, 121.89, 121.34, 118.70, 114.71, 113.48, 98.15, 77.52, 77.20, 76.88, 72.81, 72.42, 70.59, 70.53, 70.10, 69.97, 68.94, 67.37, 66.09, 59.94, 58.51, 49.92, 49.32, 47.73, 46.92, 46.01, 31.63, 29.61, 27.43, 26.76, 26.62, 25.65, 24.57, 23.08, 22.98, 22.82, 22.73, 13.94, 7.86. MALDI-TOF-HRMS, calcd for $(C_{96}H_{101}N_{12}O_{12})^{+}$: 1613.7662, found 1613.7502 $(M + H)^{+}$.

(4*S*)-4,11-diethyl-4-hydroxy-3,14-dioxo-3,4,12,14-tetrahydro-1*H*-pyrano[3',4':6,7]indolizino[1,2-*b*]quinolin-9-yl 4-[([2-(2-{2-[(5a*R*,6*S*,6a*S*)-6-[({(biphenyl-4-ylmethyl)[4-(3,5-bis{[bis(pyridin-2-ylmethyl)amino]methyl}phenoxy)butyl]carbamoyl}oxy)methyl]-5,5a,6,6a,7,8-hexahydrocyclo-

propa[5,6]cycloocta[1,2-*d*][1,2,3]triazol-1(4*H*)-yl]ethoxy}ethoxy)ethyl]{[1-(4-chlorophenyl)cyclohexyl]carbonyl}amino)methyl]benzoate (precursor of conjugate 3)

Following *General procedure B,* precursor of conjugate **3** (sticky oil, 100 mg, 49%) was obtained via SPAAC reaction between intermediate **16** (103mg) and **10** (110 mg). ^{1}H NMR (400 MHz, cdcl$_3$) δ 8.50 (d, *J* = 4.8 Hz, 4H), 8.30 (d, *J* = 9.1 Hz, 1H), 8.18 (d, *J* = 7.9 Hz, 2H), 7.96 (s, 1H), 7.73 – 7.65 (m, 2H), 7.57 (m, 12H), 7.41 (m, 2H), 7.34 – 7.21 (m, 9H), 7.14 – 7.08 (m, 6H), 6.82 (s, 2H), 5.74 (d, *J* = 16.3 Hz, 1H), 5.34 – 5.25 (m, 3H), 4.54 (s, 2H), 4.34 (m, 4H), 4.21 (m, 2H), 3.94 (m 2H), 3.80 (m, 11H), 3.64 (m, 6H), 3.39 (m, 5H), 3.20 – 3.11 (m, 3H), 2.87 (m, 2H), 2.64 (m, 2H), 2.28 (m, 6H), 1.99 – 1.85 (m, 2H), 1.68 (m, 12H), 1.40 (t, *J* = 7.6 Hz, 3H), 1.04 (m, 7H). ^{13}C NMR (101 MHz, cdcl$_3$) δ 175.04, 173.90, 164.86, 159.84, 159.19, 157.73, 152.08, 150.42, 149.96, 149.04, 147.60, 146.91, 145.41, 144.48, 140.68, 140.32, 136.50, 133.92, 132.44, 132.28, 130.66, 129.16, 128.91, 128.37, 127.71, 127.61, 127.44, 127.31, 126.90, 125.59, 122.82, 122.04, 121.51, 118.77, 114.91, 113.61, 98.26, 72.92, 70.70, 70.45, 70.13, 69.02, 67.51, 66.37, 63.41, 60.11, 58.68, 53.55, 51.17, 50.08, 49.50, 47.77, 37.00, 31.76, 29.79, 26.78, 26.02, 25.94, 25.12, 24.73, 23.73, 23.3, 23.25, 22.78, 22.22, 20.08, 19.54, 17.82, 14.11, 7.97. MALDI-TOF-HRMS, calcd for $(C_{109}H_{115}ClN_{13}O_{12})^+$: 1832.8477, found 1832.8579 $(M + H)^+$.

(4*S*)-4,11-diethyl-4-hydroxy-3,14-dioxo-3,4,12,14-tetrahydro-1*H*-pyrano[3',4':6,7]indolizino[1,2-*b*]quinolin-9-yl 4-[([2-(2-{2-[(5a*R*,6*R*,6a*S*)-6-[({(biphenyl-4-ylmethyl)[4-(3,5-bis{[bis(pyridin-2-ylmethyl)amino]methyl}phenoxy)butyl]carbamoyl}oxy)methyl]-5,5a,6,6a,7,8-hexahydrocyclo-propa[5,6]cycloocta[1,2-*d*][1,2,3]triazol-1(4*H*)-yl]ethoxy}ethoxy)ethyl]{[1-(4-chlorophenyl) cyclohexyl]carbonyl}amino)methyl]benzoate (precursor of conjugate 4)

Following *General procedure B,* precursor of conjugate **4** (sticky oil, 392 mg, 65%) was obtained via SPAAC reaction between intermediate **17** (304 mg) and **10** (315 mg). ^{1}H NMR (600 MHz, CDCl$_3$) δ 8.52 – 8.47 (m, 4H), 8.29 (d, *J* = 9.1 Hz, 1H), 8.17 (m, 2H), 7.99 – 7.95 (m, 1H), 7.68 (m, 2H), 7.57 (m, 12H), 7.42 (m, 2H), 7.37 – 7.27 (m, 6H), 7.23 (m, 3H), 7.11 (m, 6H), 6.81 (s, 2H), 5.76 (d, *J* = 16.2 Hz,

1H), 5.34 – 5.27 (m, 3H), 4.53 (m, 2H), 4.32 (m, 3H), 4.12 – 3.96 (m, 2H), 3.93 (m, 2H), 3.79 (m, 11H), 3.63 (m, 6H), 3.51 – 3.37 (m, 5H), 3.34 – 3.25 (m, 1H), 3.17 (m, 2H), 3.05 (m, 1H), 2.93 – 2.73 (m, 2H), 2.58 (m, 1H), 2.37 – 2.20 (m, 4H), 1.94 – 1.85 (m, 4H), 1.66 (m, 12H), 1.41 (t, J = 7.7 Hz, 3H), 1.05 (t, J = 7.4 Hz, 3H), 0.97 – 0.69 (m, 4H). ^{13}C NMR (151 MHz, $CDCl_3$) δ 175.16, 174.08, 164.89, 159.92, 159.27, 157.84, 156.73, 152.18, 150.43, 150.04, 149.12, 147.73, 147.08, 145.49, 144.92, 140.76, 140.40, 137.40, 136.57, 134.22, 132.52, 132.37, 130.75, 129.24, 129.00, 128.38, 127.78, 127.71, 127.50, 127.39, 127.14, 126.98, 126.84, 125.67, 122.90, 122.11, 121.56, 118.78, 115.01, 113.69, 98.25, 70.73, 70.52, 70.17, 69.12, 67.60, 66.52, 60.19, 58.75, 53.60, 52.71, 51.24, 50.10, 49.59, 47.83, 47.13, 46.12, 45.97, 37.03, 31.81, 29.86, 27.62, 26.95, 26.86, 26.00, 25.88, 25.17, 24.77, 23.83, 23.37, 23.08, 22.93, 14.18, 8.02. ESI-HRMS, calcd for $C_{109}H_{114}ClN_{13}O_{12}$:1831.8398, found 1831.8465 (M).

(4*S*)-4,11-diethyl-4-hydroxy-3,14-dioxo-3,4,12,14-tetrahydro-1*H*-pyrano[3',4':6,7]indolizino[1,2-*b*]quinolin-9-yl 4-[([2-(2-{2-[(5a*R*,6*R*,6a*S*)-6-({[(4-{[4-(3,5-bis{[bis(pyridin-2-ylmethyl)amino] methyl}phenoxy)butyl][4-(*tert*-butylcarbamoyl)tetrahydro-2*H*-pyran-4-yl]amino}-4-oxobutyl) (methyl)carbamoyl]oxy}methyl)-5,5a,6,6a,7,8-hexahydrocyclopropa[5,6]cycloocta[1,2-*d*][1,2,3]triazol-1(4*H*)-yl]ethoxy}ethoxy)ethyl]{[1-(4-chlorophenyl)cyclohexyl]carbonyl}amino) methyl]benzoate (precursor of conjugate 5)

Following *General procedure B,* precursor of conjugate **5** (sticky oil, 189 mg, 55%) was obtained via SPAAC reaction between intermediate **18** (184 mg) and **10** (175 mg). ^{1}H NMR (400 MHz, $cdcl_3$) δ 8.51 (m, 4H), 8.31 (d, J = 9.1 Hz, 1H), 8.20 (d, J = 7.3 Hz, 2H), 7.97 (s, 1H), 7.73 – 7.57 (m, 10H), 7.43 – 7.24 (m, 5H), 7.14 (s, 5H), 6.84 (s, 3H), 5.73 (d, J = 16.5 Hz, 1H), 5.35 – 5.24 (m, 3H),**35** 4.92 (m, 1H), 4.72 (m, 1H), 4.38 (s, 4H), 3.97 (s, 4H), 3.80 (m, 12H), 3.67 (s, 6H), 3.42 (m, 6H), 3.28 (m, 2H), 3.18 (m, 2H), 3.07 (m, 2H), 2.89 (s, 3H), 2.86 – 2.74 (m, 2H), 2.72 – 2.57 (m, 2H), 2.50 (m, 2H), 2.39 (m, 6H), 2.01 – 1.84 (m, 8H), 1.78 (m, 6H), 1.42 (t, J = 6.8 Hz, 3H), 1.32 (s, 13H), 1.04 (t, J = 6.6 Hz, 3H), 0.88 (m, 2H), 0.74 (m, 1H). ^{13}C NMR (101 MHz, $cdcl_3$) δ 174.91, 174.30, 173.63, 172.03, 164.71,

159.66, 158.83, 157.58, 156.58, 151.93, 150.37, 149.81, 148.91, 147.40, 146.66, 145.29, 144.72, 144.35, 140.58, 136.41, 134.07, 132.29, 132.10, 130.53, 129.03, 127.45, 127.35, 126.83, 125.44, 122.72, 121.95, 121.61, 118.73, 114.77, 113.47, 98.22, 72.84, 70.55, 70.32, 69.99, 68.93, 68.77, 67.04, 66.15, 64.72, 63.39, 59.96, 58.51, 52.55, 51.03, 50.79, 49.37, 48.22, 47.66, 46.60, 44.50, 37.75, 36.87, 34.11, 33.94, 32.24, 31.64, 28.59, 27.74, 27.47, 26.81, 26.74, 25.81, 25.69, 24.34, 23.61, 23.15, 22.87, 22 .65, 13.99, 7.87. ESI-HRMS, calcd for $(C_{111}H_{129}ClN_{15}O_{15})^-$: 1946.9481, found1946.9544 $(M-H)^-$.

[(5a*R*,6*R*,6a*S*)-1-(2-{2-[2-({[1-(4-chlorophenyl)cyclohexyl]carbonyl}[4-({[(4*S*)-4,11-diethyl-4-hydroxy-3,14-dioxo-3,4,12,14-tetrahydro-1*H*-pyrano[3',4':6,7]indolizino[1,2-*b*]quinolin-9-yl]oxy}carbonyl)benzyl]amino)ethoxy]ethoxy}ethyl)-1,4,5,5a,6,6a,7,8-octahydrocyclopropa[5,6] cycloocta[1,2-*d*][1,2,3]triazol-6-yl]methyl 4-{[4-(3,5-bis{[bis(pyridin-2-ylmethyl)amino]methyl} phenoxy)butyl][4-(*tert*-butylcarbamoyl)tetrahydro-2*H*-pyran-4-yl]carbamoyl}piperidine-1-carboxylate (precursor of conjugate 6)

Following *General procedure B,* precursor of conjugate **6** (sticky oil, 149 mg, 66%) was obtained via SPAAC reaction between intermediate **19** (123 mg) and **10** (115 mg). ^{1}H NMR (400 MHz, cdcl$_3$) δ 8.51 (d, *J* = 4.7 Hz, 4H), 8.30 (d, *J* = 9.2 Hz, 1H), 8.18 (d, *J* = 7.9 Hz, 2H), 7.97 (m, 1H), 7.68 (m, 2H), 7.65 – 7.55 (m, 8H), 7.34 – 7.22 (m, 5H), 7.16 – 7.11 (m, 5H), 6.83 (s, 3H), 5.75 (d, *J* = 16.3 Hz, 1H), 5.35 – 5.25 (m, 3H), 4.71 (br, 1H), 4.37 (t, *J* = 5.7 Hz, 2H), 4.16 (m, 2H), 3.98 (m, 2H), 3.80 (m, 16H), 3.65 (s, 6H), 3.59 – 3.29 (m, 8H), 3.22 – 3.15 (m, 2H), 3.08 (m, 1H), 2.90 (m, 1H), 2.82 – 2.59 (m, 5H), 2.35 (m, 8H), 1.94 (m, 4H), 1.81 – 1.62 (m, 14H), 1.42 (t, *J* = 7.6 Hz, 3H), 1.29 (s, 13H), 1.04 (t, *J* = 7.4 Hz, 3H), 0.92 – 0.82 (m, 2H), 0.75 (m, *J* = 5.8 Hz, 1H). ^{13}C NMR (101 MHz, cdcl$_3$) δ 177.08, 174.91, 173.61, 171.70, 164.68, 159.58, 158.73, 157.56, 155.31, 151.90, 150.36, 149.78, 148.91, 147.36, 146.63, 145.28, 144.73, 144.31, 143.79, 140.59, 136.40, 134.10, 132.26, 132.06, 130.52, 129.01, 127.80, 127.43, 127.33, 126.79, 125.43, 122.71, 121.95, 121.75, 118.72, 114.75, 113.42, 98.21, 72.83, 70.53, 70.30, 70.00, 68.92, 66.88, 66.11, 64.67, 63.61, 59.90, 58.44, 52.59, 51.02, 50.75, 49.35, 47.66, 46.57, 44.29, 43.19, 40.89,

36.86, 34.08, 31.61, 28.76, 28.54, 27.44, 26.78, 26.59, 25.79, 25.67, 24.28, 23.59, 23.13, 22.87, 22.66, 13.98, 7.86. ESI-HRMS, calcd for $(C_{112}H_{129}ClN_{15}O_{15})^-$: 1958.9481, found 1958.9440 $(M-H)^-$.

[(5a*R*,6*R*,6a*S*)-1-(2-{2-[2-({[1-(4-chlorophenyl)cyclohexyl]carbonyl}[4-({[(4*S*)-4,11-diethyl-4-hydroxy-3,14-dioxo-3,4,12,14-tetrahydro-1*H*-pyrano[3',4':6,7]indolizino[1,2-*b*]quinolin-9-yl]oxy}carbonyl)benzyl]amino)ethoxy]ethoxy}ethyl)-1,4,5,5a,6,6a,7,8-octahydrocyclopropa[5,6] cycloocta[1,2-*d*][1,2,3]triazol-6-yl]methyl 4-{[4-(3,5-bis{[bis(pyridin-2-ylmethyl)amino]methyl} phenoxy)butyl][(2*E*)-but-2-enoyl]amino}-4-(*tert*-butylcarbamoyl)piperidine-1-carboxylate (precursor of conjugate 7)

Following *General procedure B,* precursor of conjugate **7** (sticky oil, 267 mg, 60%) was obtained via SPAAC reaction between intermediate **20** (235 mg) and **10** (231 mg). ^1H NMR (600 MHz, $CDCl_3$) δ 8.52 – 8.49 (m, 4H), 8.30 (d, *J* = 9.1 Hz, 1H), 8.18 (d, *J* = 6.7 Hz, 2H), 7.97 (s, 1H), 7.72 – 7.66 (m, 2H), 7.63 – 7.56 (m, 8H), 7.30 (m, 2H), 7.25 – 7.17 (m, 3H), 7.16 – 7.10 (m, 6H), 6.89 – 6.78 (m, 3H), 6.21 (d, *J* = 14.9 Hz, 1H), 5.76 (d, *J* = 16.2 Hz, 1H), 5.32 (d, *J* = 16.2 Hz, 1H), 5.29 (s, 2H), 4.36 (m, 3H), 3.93 (m, 4H), 3.80 (s, 10H), 3.65 (s, 6H), 3.55 – 3.38 (m, 8H), 3.18 (m, 2H), 3.13 – 3.00 (m, 1H), 2.62 (s, 1H), 2.47 – 2.21 (m, 4H), 2.04 (m, 2H), 1.99 – 1.85 (m, 2H), 1.83 – 1.51 (m, 22H), 1.42 (t, *J* = 7.7 Hz, 3H), 1.29 (s, 11H), 1.05 (t, *J* = 7.4 Hz, 3H), 0.93 – 0.80 (m, 3H). ^{13}C NMR (101 MHz, $CDCl_3$) δ 174.13, 159.85, 157.86, 155.75, 150.42, 149.15, 147.75, 145.53, 145.02, 143.00, 140.79, 136.64, 132.39, 130.75, 129.26, 127.74, 127.51, 125.73, 124.44, 122.91, 122.18, 121.81, 118.76, 115.04, 113.66, 98.25, 72.96, 70.54, 69.12, 67.21, 66.55, 64.24, 60.15, 58.73, 51.03, 49.61, 47.87, 45.39, 32.98, 31.79, 29.88, 28.74, 27.68, 27.02, 26.87, 25.91, 24.51, 23.40, 23.10, 22.88, 18.35, 14.22, 8.03. ESI-HRMS, calcd for $C_{110}H_{126}ClN_{15}O_{14}$: 1915.9297, found 1915.9276 (M).

[(5a*R*,6*R*,6a*S*)-1-(2-{2-[2-({[1-(4-chlorophenyl)cyclohexyl]carbonyl}[4-({[(4*S*)-4,11-diethyl-4-hydroxy-3,14-dioxo-3,4,12,14-tetrahydro-1*H*-pyrano[3',4':6,7]indolizino[1,2-*b*]quinolin-9-yl]oxy}carbonyl)benzyl]amino)ethoxy]ethoxy}ethyl)-1,4,5,5a,6,6a,7,8-octahydrocyclopropa[5,6] cycloocta[1,2-*d*][1,2,3]triazol-6-yl]methyl 4-{[4-(3,5-bis{[bis(pyridin-2-ylmethyl)amino]methyl} phenoxy)butyl][(4'-hydroxybiphenyl-4-yl)carbonyl]amino}-4-{[(diethoxyphosphoryl)methyl] carbamoyl}piperidine-1-carboxylate (precursor of conjugate 8)

Following *General procedure B,* precursor of conjugate **8** (sticky oil, 167 mg, 56%) was obtained via SPAAC reaction between intermediate **21** (173 mg) and **10** (139 mg). ^{1}H NMR (400 MHz, cdcl$_3$) δ 8.54 – 8.46 (m, 4H), 8.29 (m, 2H), 8.18 (d, *J* = 7.8 Hz, 2H), 7.96 (s, 1H), 7.72 – 7.63 (m, 2H), 7.63 – 7.53 (m, 8H), 7.50 (d, J = 8.2 Hz, 2H), 7.46 (d, J = 8.1 Hz, 2H), 7.35 (d, J = 8.4 Hz, 2H), 7.32 – 7.28 (m, 2H), 7.24 (d, *J* = 8.4 Hz, 2H), 7.14 (m, 6H), 6.88 (d, *J* = 8.4 Hz, 2H), 6.47 (s, 2H), 5.74 (d, *J* = 16.3 Hz, 1H), 5.30 (m, 3H), 4.71 (br, 1H), 4.37 (m, 2H), 4.16 (m, *J* = 7.3 Hz, 4H), 4.01 (br, 1H), 3.88 (br, 1H), 3.80 (m, 4H), 3.72 (s, 12H), 3.59 (m, 2H), 3.48 (d, *J* = 5.8 Hz, 8H), 3.39 (s, 4H), 3.16 (m, 2H), 3.08 (m, 1H), 2.97 (m, 1H), 2.85 – 2.75 (m, 1H), 2.66 (m, 1H), 2.54 – 2.18 (m, 10H), 1.92 (m, 2H), 1.92 (m, 2H), 2.51-2.21(m, 10H), 1.54 (dm, 2H), 1.40 (t, *J* = 7.6 Hz, 3H), 1.34 (m, 8H), 1.04 (t, *J* = 7.3 Hz, 3H), 0.89 (m, 2H), 0.78 (m, 1H). ^{13}C NMR (101 MHz, cdcl3) δ 175.83, 175.04, 173.80, 173.72, 164.79, 159.62, 158.80, 157.74, 157.69, 155.59, 151.99, 150.50, 149.87, 148.78, 147.48, 146.76, 145.41, 144.80, 144.40, 143.24, 140.49, 136.73, 134.55, 134.26, 132.37, 132.16, 131.03, 130.63, 129.11, 128.10, 128.05, 127.55, 127.40, 126.87, 126.51, 125.55, 122.88, 122.18, 121.08, 118.75, 117.18, 114.88, 113.37, 98.36, 72.91, 70.60, 70.39, 70.06 (d, *J* = 89 Hz) 68.94, 66.43, 66.25, 63.28, 62.64 (*J* = 6 Hz), 59.79, 58.52, 52.58, 51.12, 49.47, 47.78, 46.69, 40.29, 36.93, 35.61, 34.07, 32.33, 31.67, 29.72, 27.51, 26.86, 26.53, 26.20, 25.87, 25.73, 24.33, 23.68, 23.22, 22.96, 22.77, 16.53 (*J* = 5 Hz), 14.07, 7.94. ESI-HRMS, calcd for $(C_{120}H_{134}ClN_{15}O_{18}P)^{+}$: 2138.9457, found 2138.9297 $(M+H)^{+}$.

General procedure C: Formation of ZnDPA conjugates with incubation of $Zn(NO_3)_2$

To a stirred solution of aforementioned conjugate precursors (1 eq) in 2 mL DCM was added 1 mL of $Zn(NO_3)_2$ (2 eq) in MeOH at room temperature. The mixture was stirred after sonicated for 5 min, and then mixture was concentrated under reduced pressure to furnish the eventual ***conjugates 1-8***, which were assayed by HPLC to confirm the purity of >95% for animal studies.

Spectra of precursor of conjugate 4.

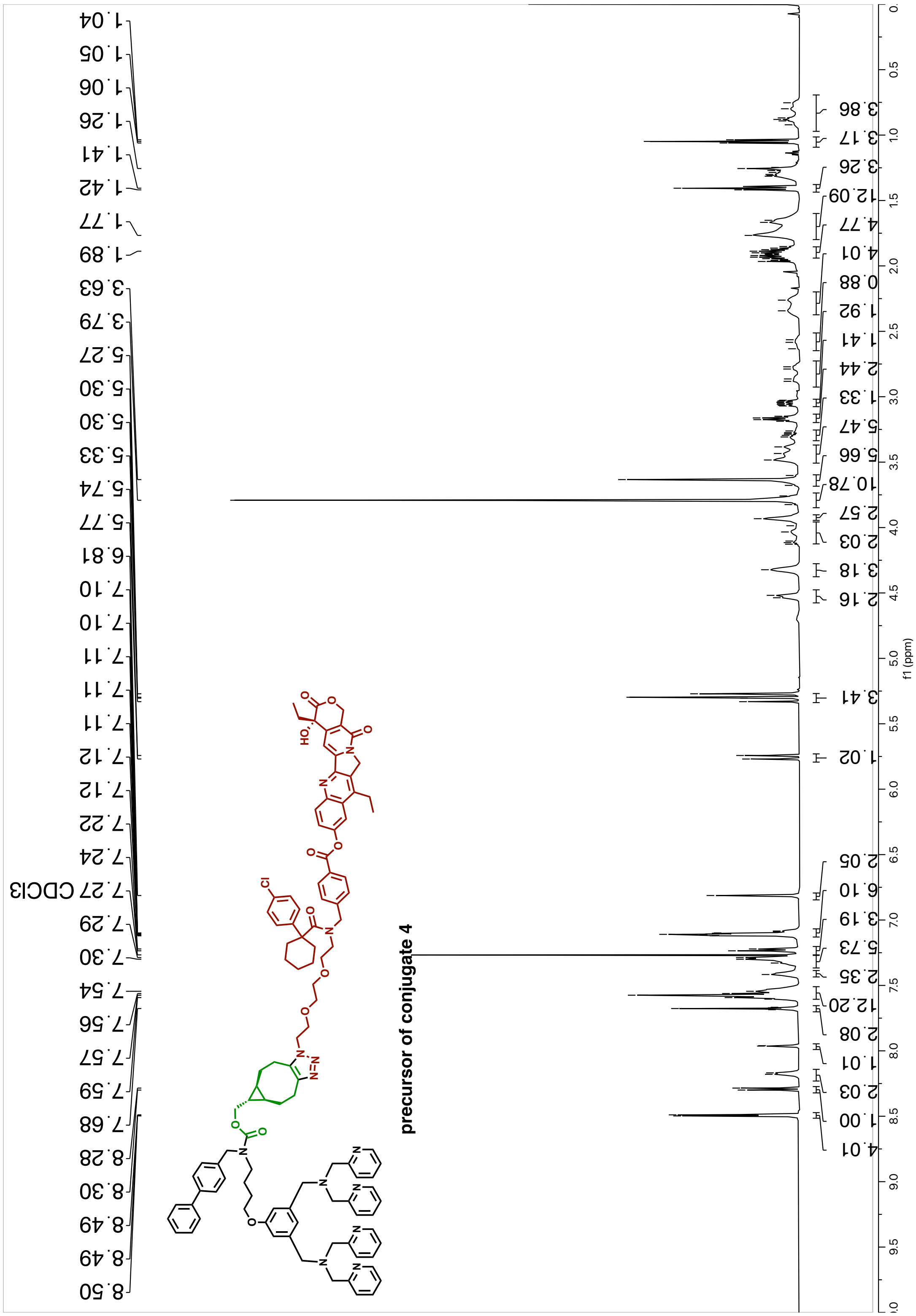

Figure S 1. The ^{1}H NMR spectra of precursor of conjugate **4**.

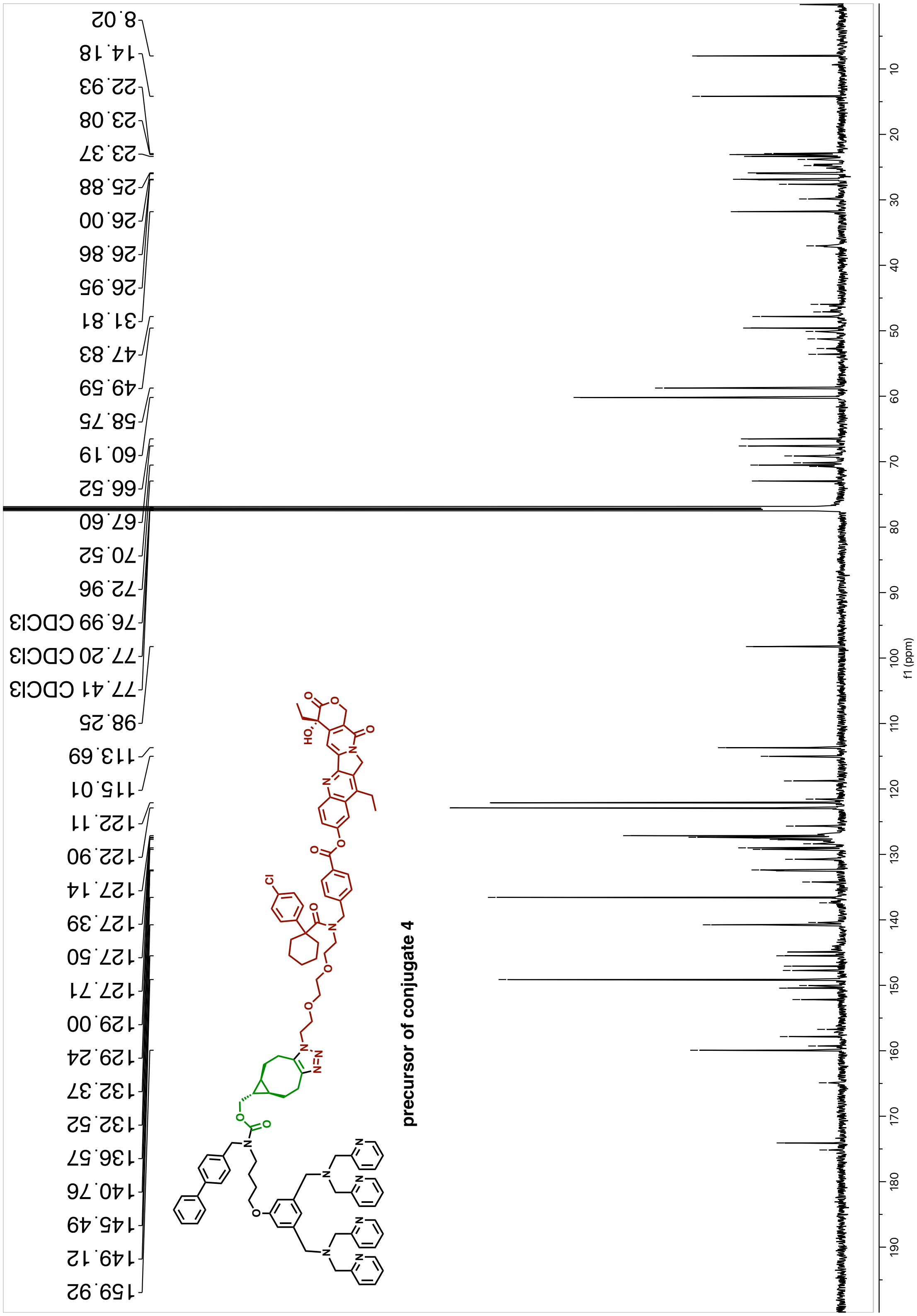

Figure S 2. The ^{13}C NMR spectra of precursor of conjugate **4**.

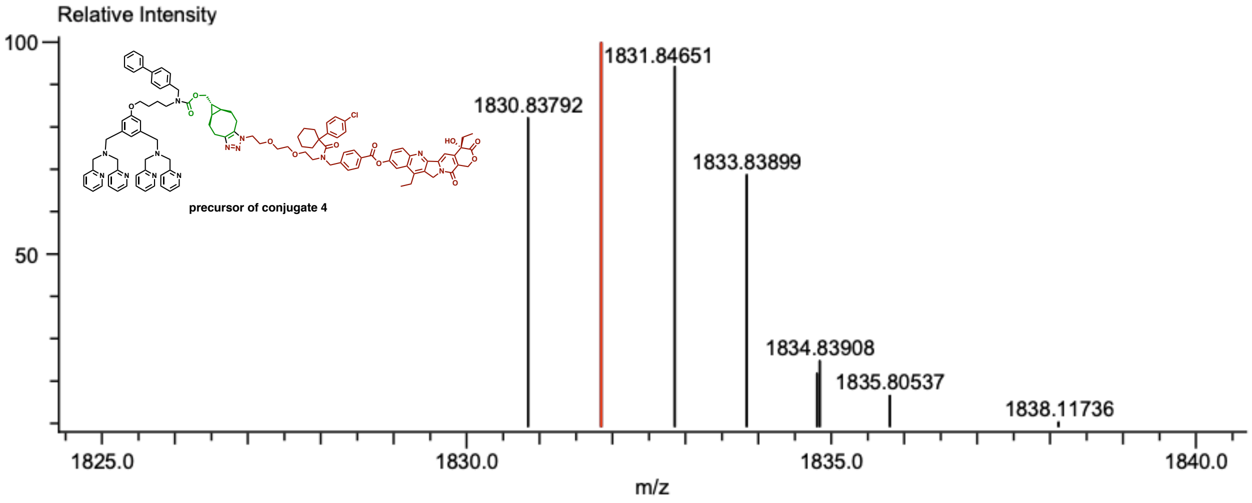

Mass	Intensity	Calc. Mass	Mass Difference [mDa]	Mass Difference [ppm]	Possible Formula
1831.84651	5846.66	1831.83984	6.67	3.64	$^{12}C_{109}{}^{1}H_{114}{}^{35}Cl_{1}{}^{14}N_{13}{}^{16}O_{12}$

Figure S 3. The high res spectra of precursor of conjugate **4**.

Spectra of compound 9.

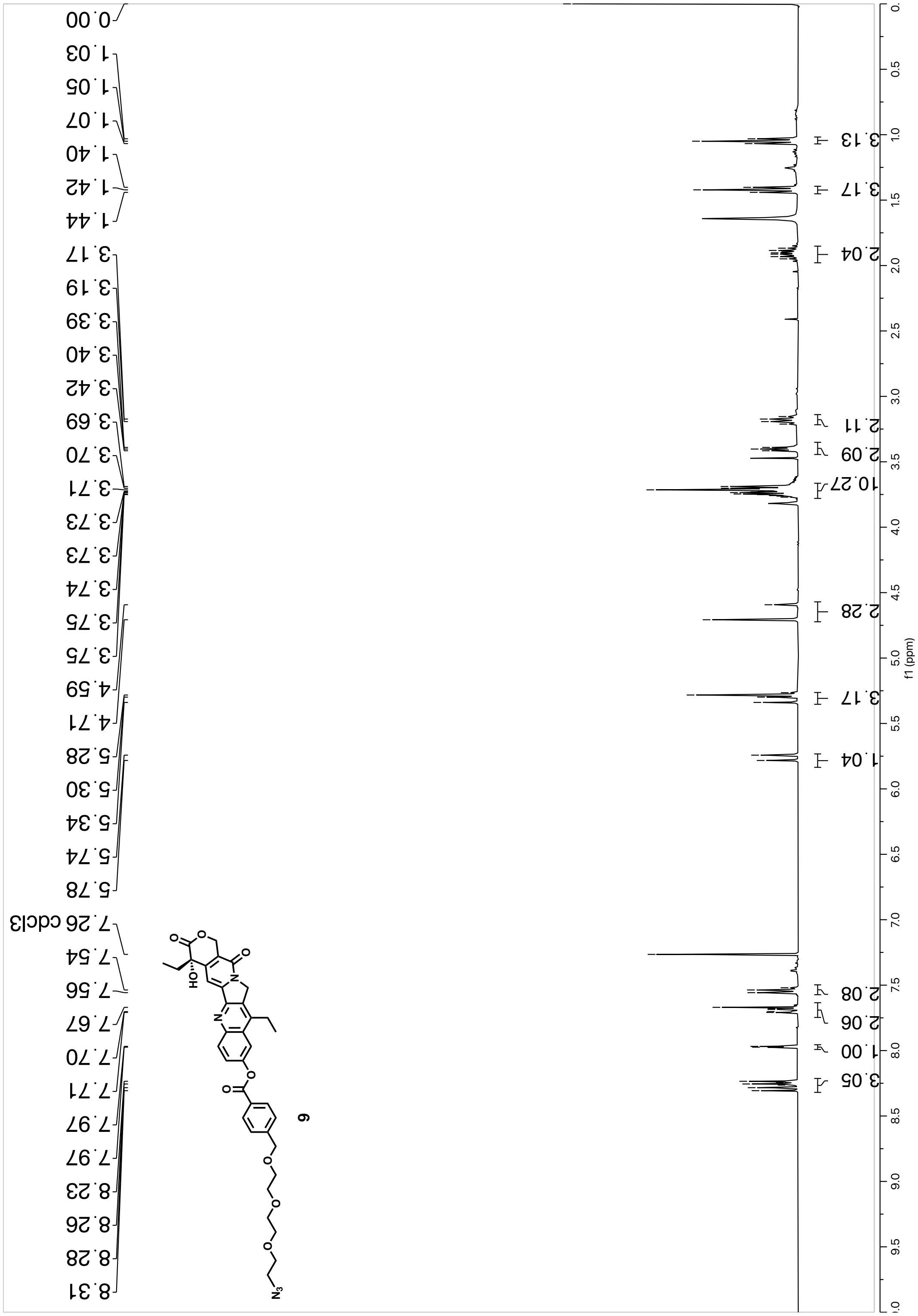

Figure S 4. The ^{1}H NMR of compound **9**.

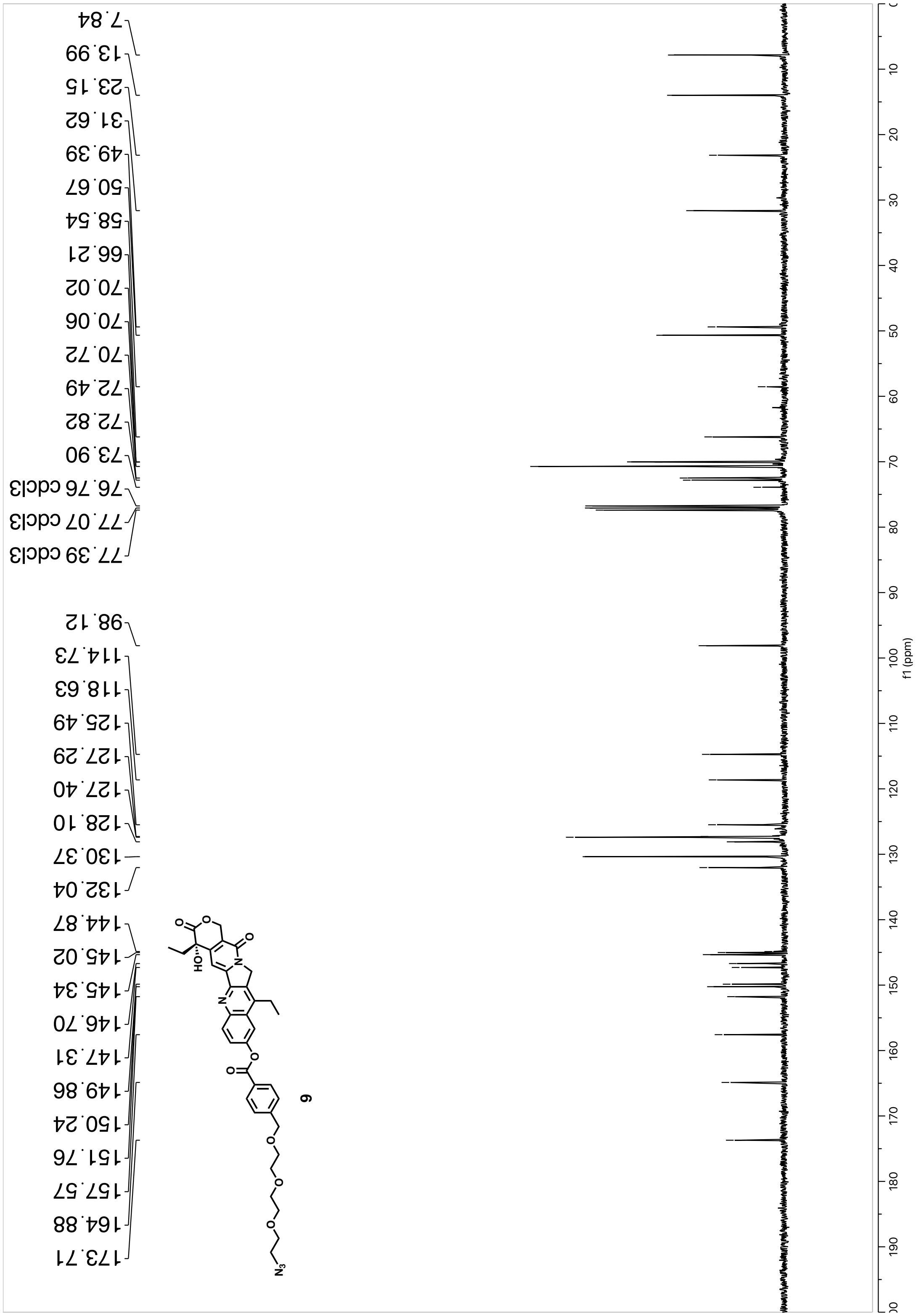

Figure S 5. The ^{13}C NMR of compound **9**.

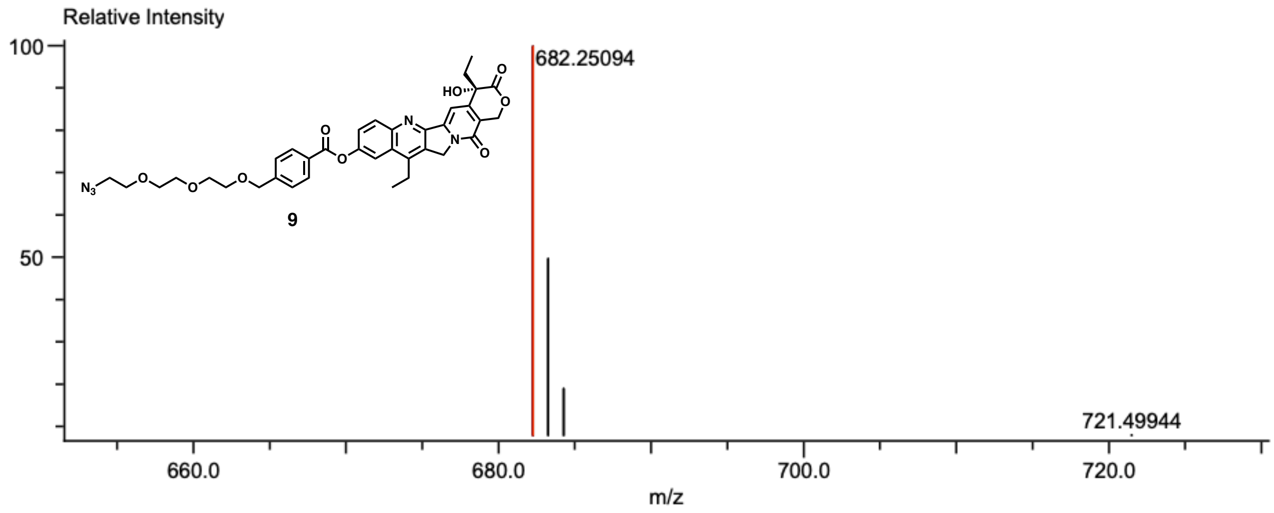

Mass	Intensity	Calc. Mass	Mass Difference [mDa]	Mass Difference [ppm]	Possible Formula
682.25094	12644.83	682.25130	-0.36	-0.53	$^{12}C_{36}{}^{1}H_{36}{}^{14}N_{5}{}^{16}O_{9}$

Figure S 6. The high res spectra of compound **9**.

Spectra of compound 10.

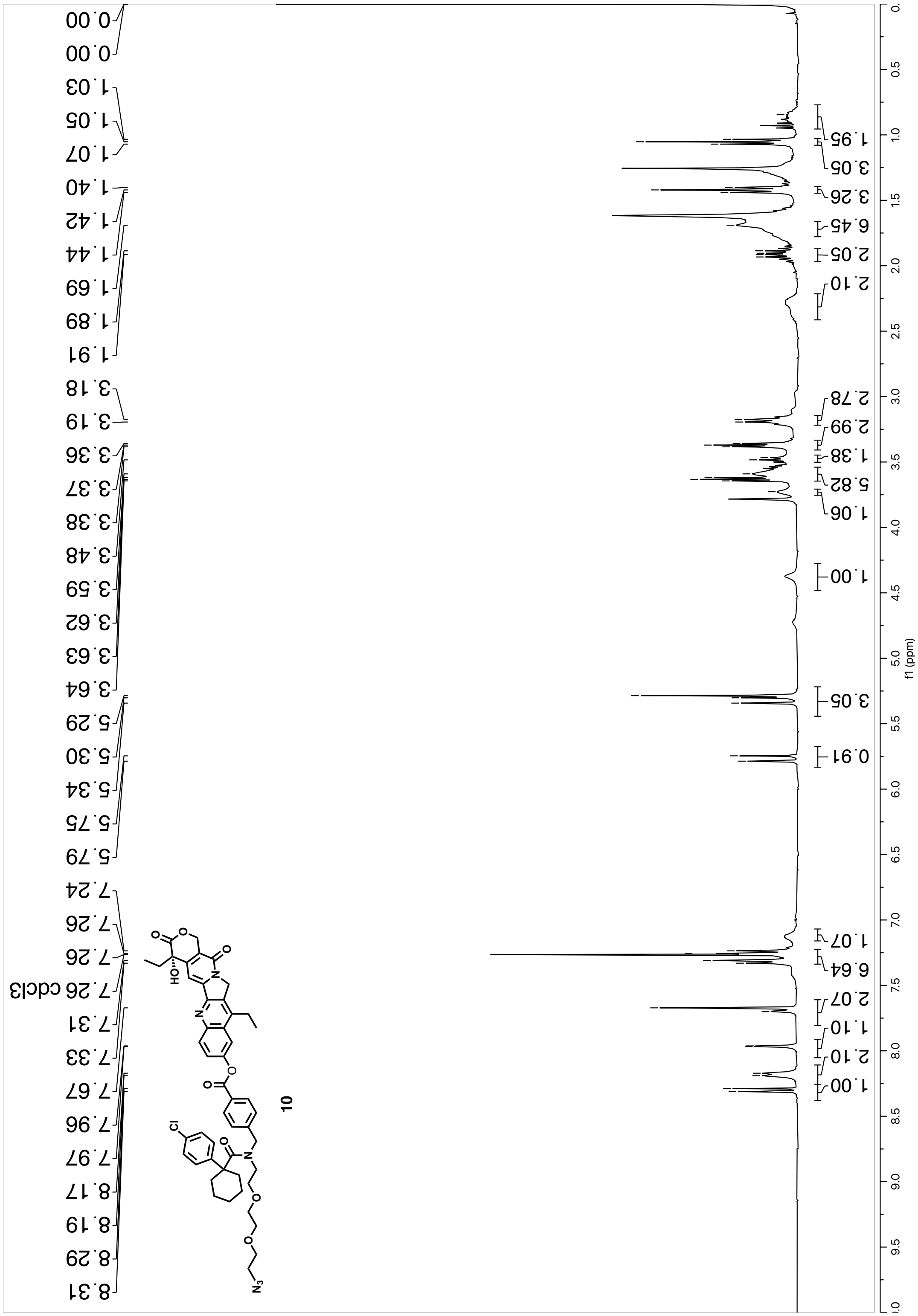

Figure S 7. The ^{1}H NMR of compound **10**.

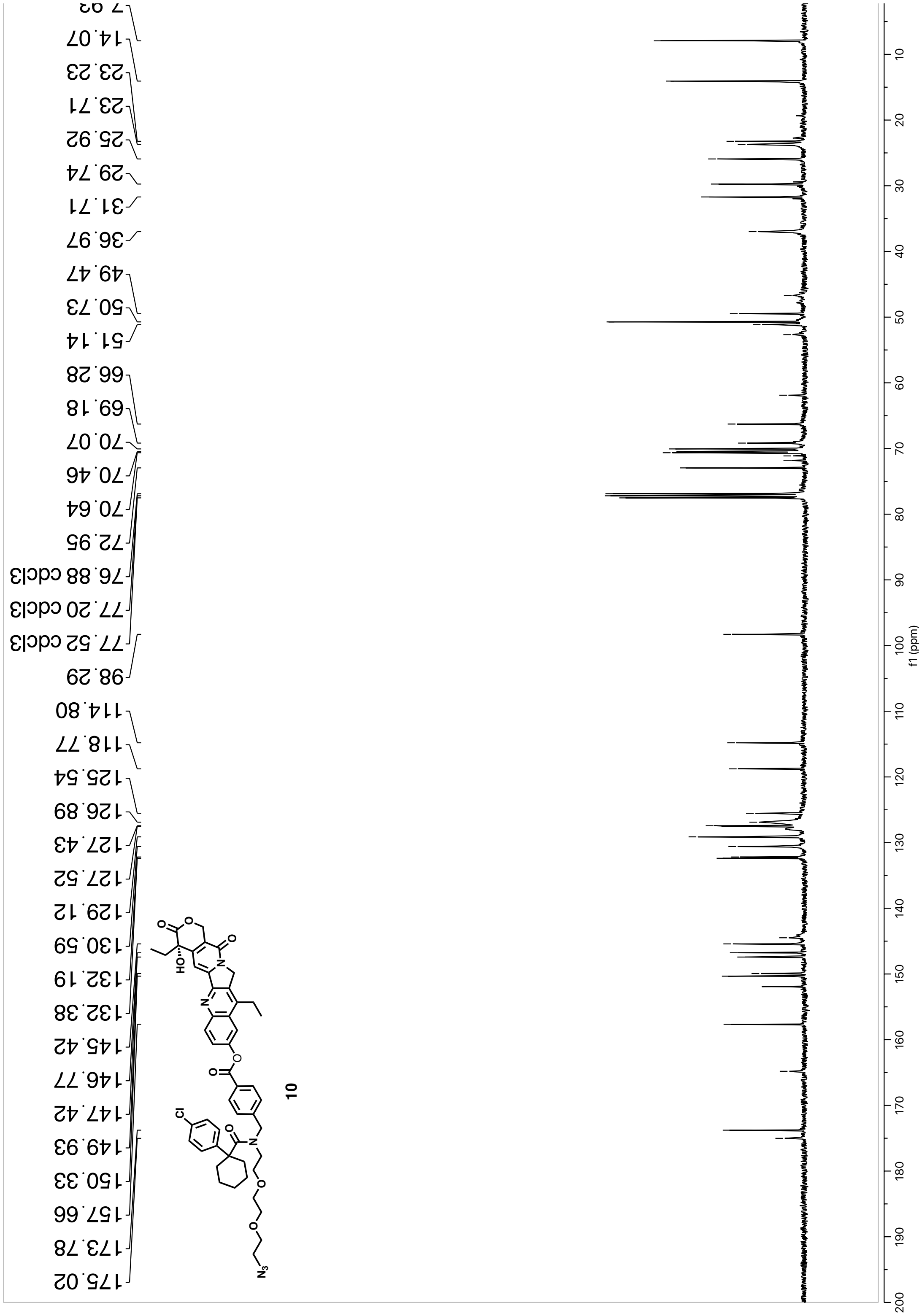

Figure S 8. The ^{13}C NMR of compound **10**.

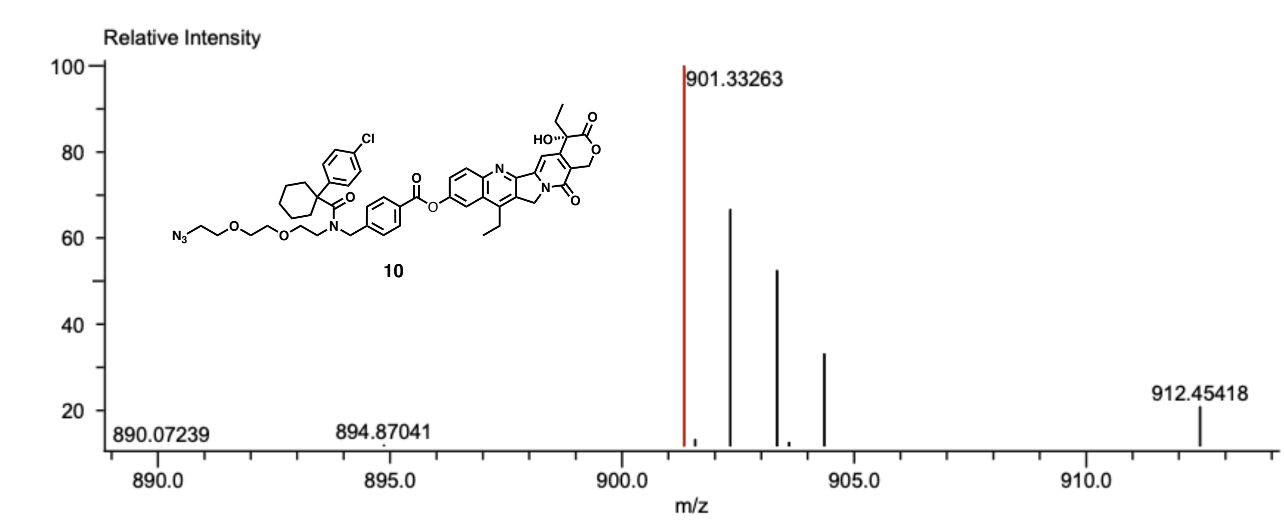

Mass	Intensity	Calc. Mass	Mass Difference [mDa]	Mass Difference [ppm]	Possible Formula
901.33263	5223.19	901.33278	-0.15	-0.16	$^{12}C_{49}{}^{1}H_{50}{}^{35}Cl_{1}{}^{14}N_{6}{}^{16}O_{9}$

Figure S 9. The high res spectra of compound **10**.

Spectra of compound 12.

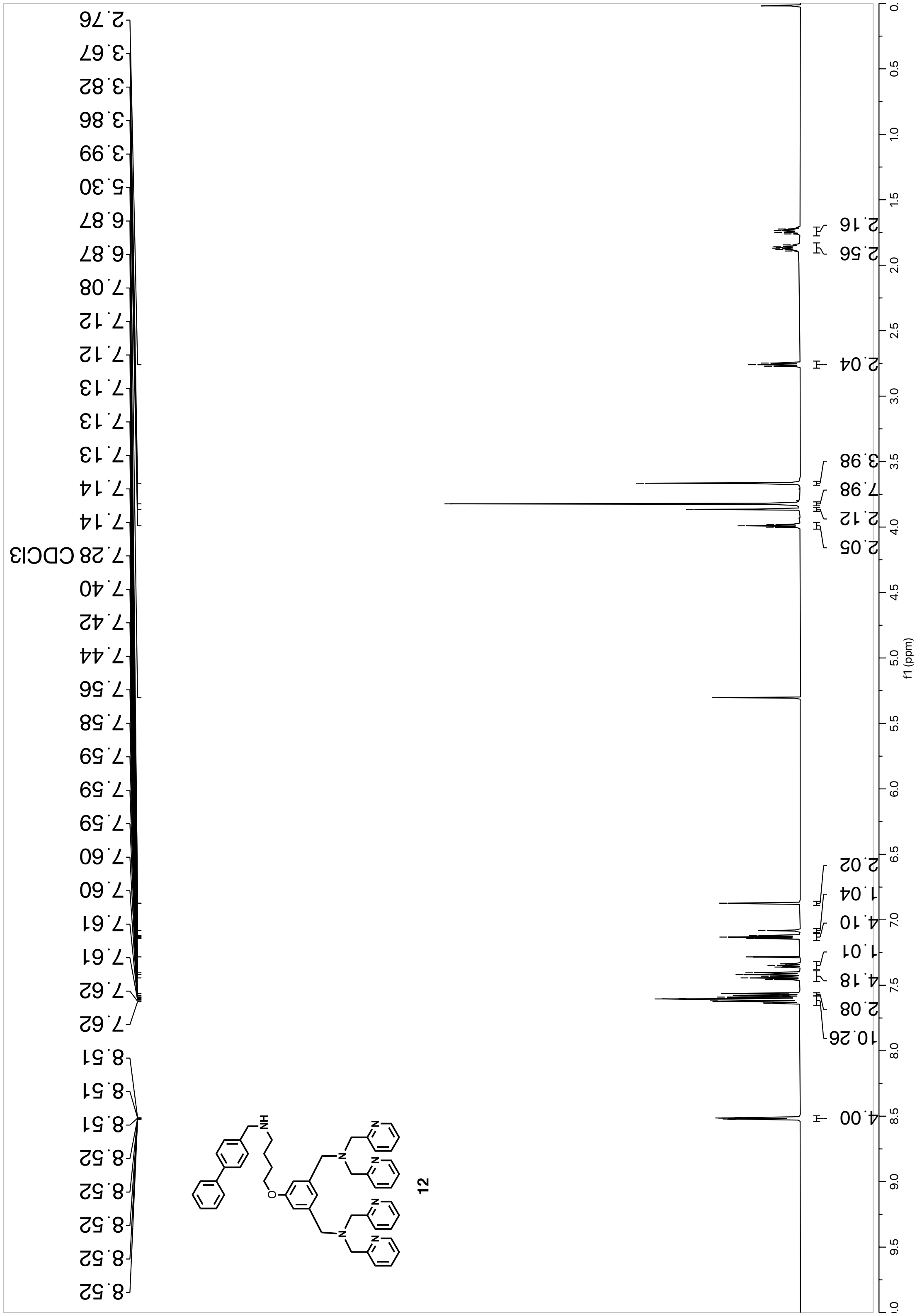

Figure S 10. The ^{1}H NMR of compound **12**.

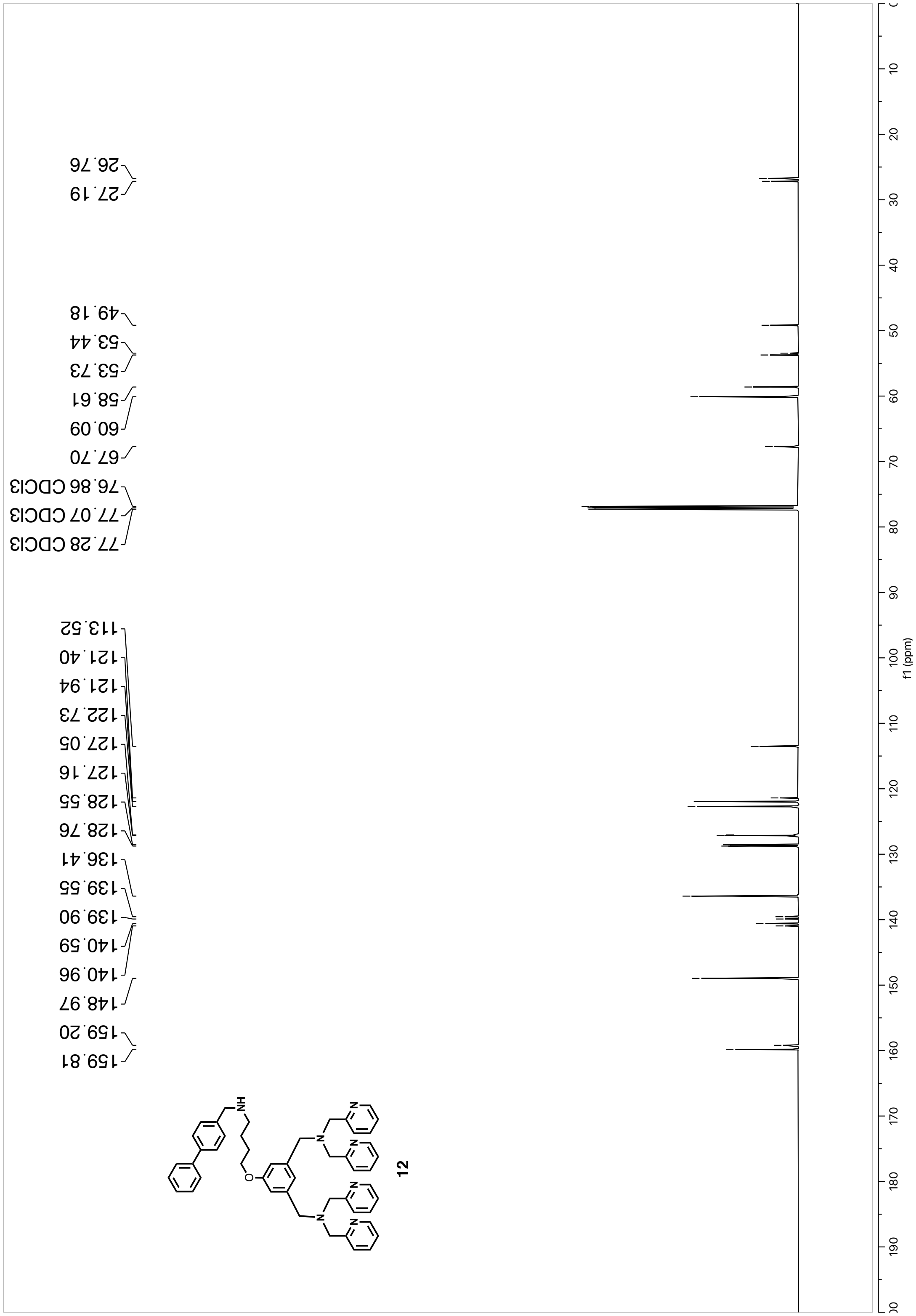

Figure S 11. The ^{13}C NMR of compound **12**.

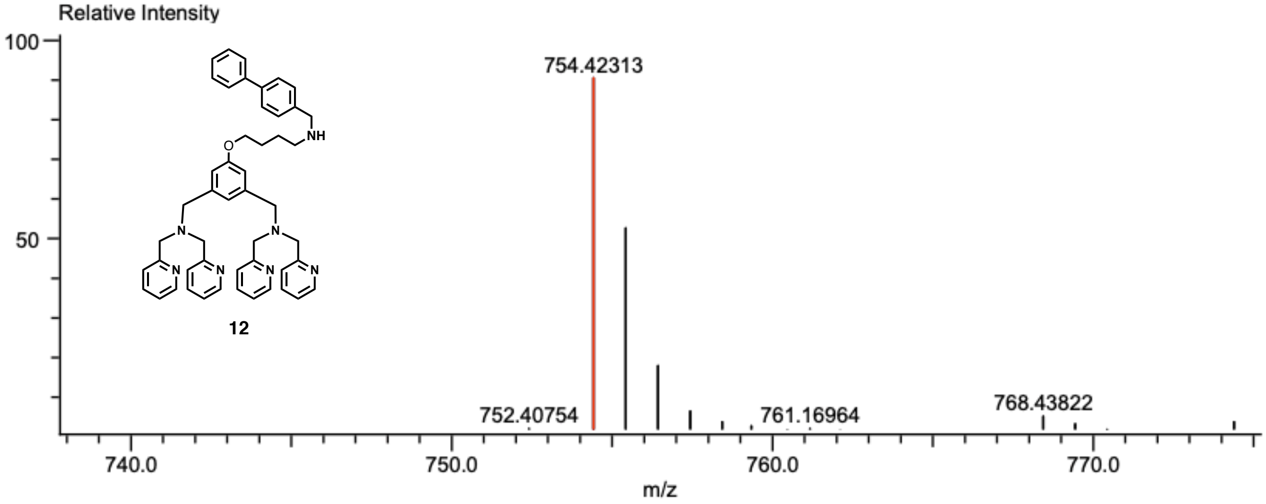

Mass	Intensity	Calc. Mass	Mass Difference [mDa]	Mass Difference [ppm]	Possible Formula
754.42313	1140021.75	754.42333	-0.20	-0.27	$^{12}C_{49}{}^{1}H_{52}{}^{14}N_{7}{}^{16}O_{1}$

Figure S 12. The high res spectra of compound **12**.

Spectra of compound 16.

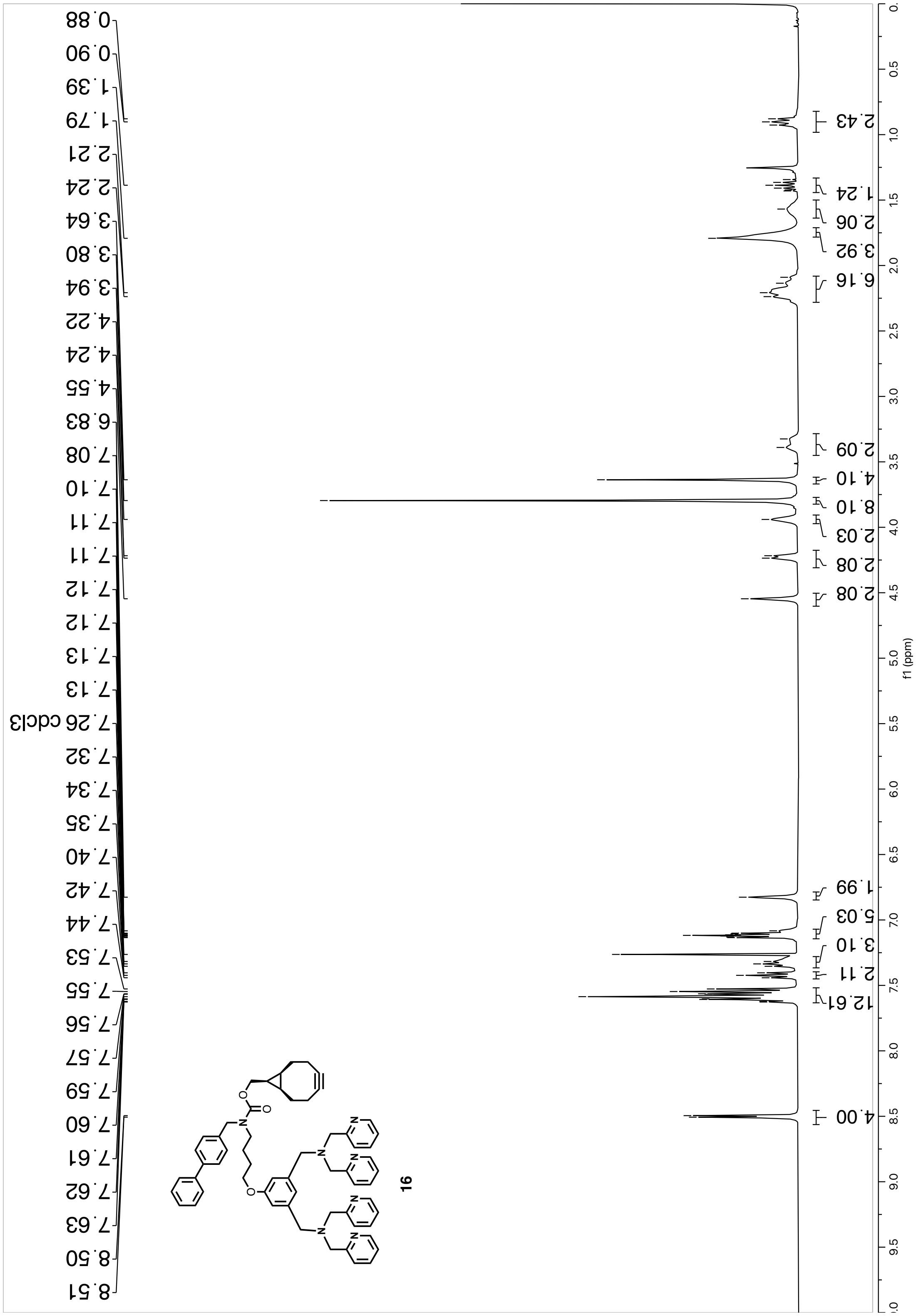

Figure S 13. The ^{1}H NMR of compound **16**.

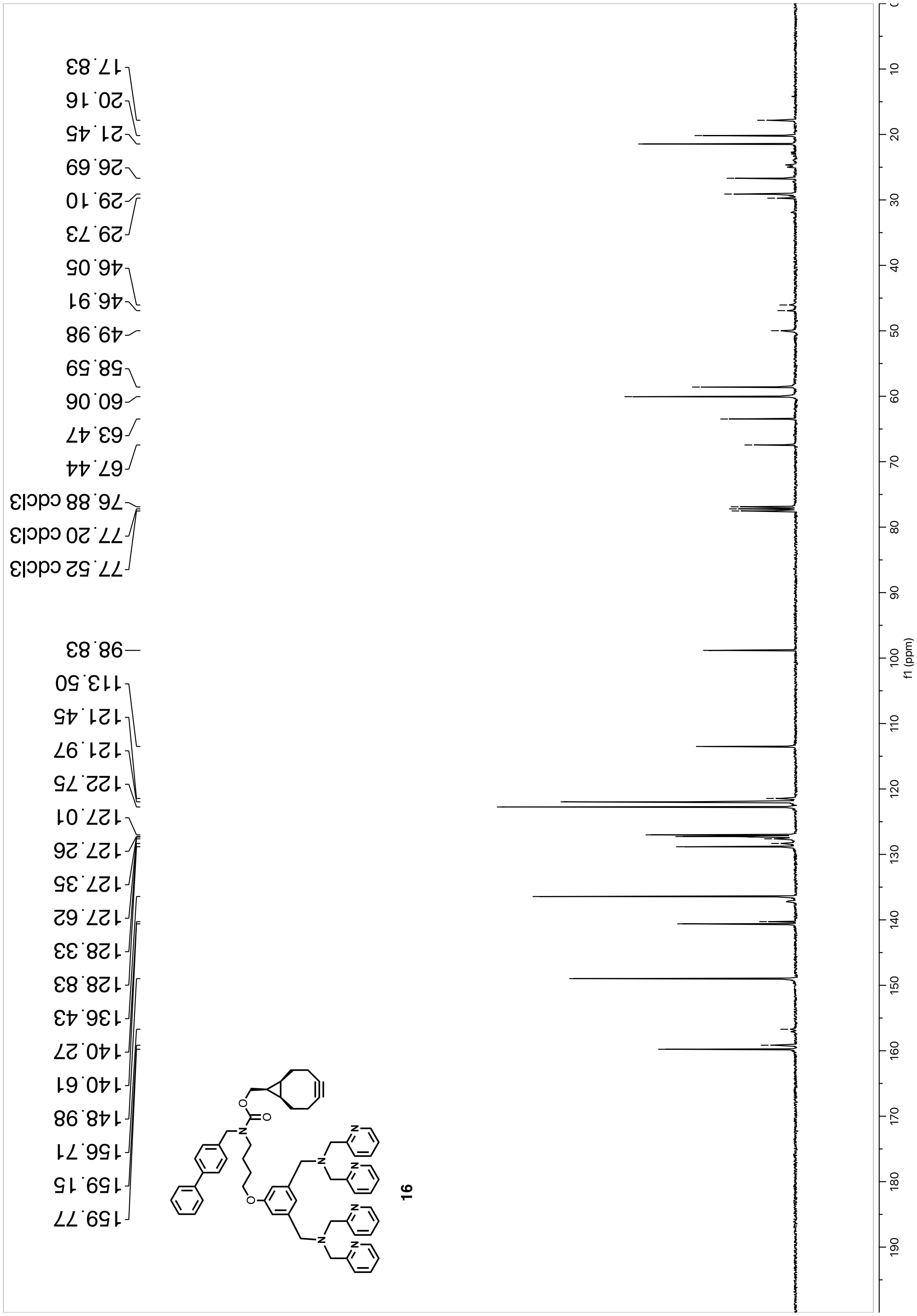

Figure S 14. The ^{13}C NMR of compound **16**.

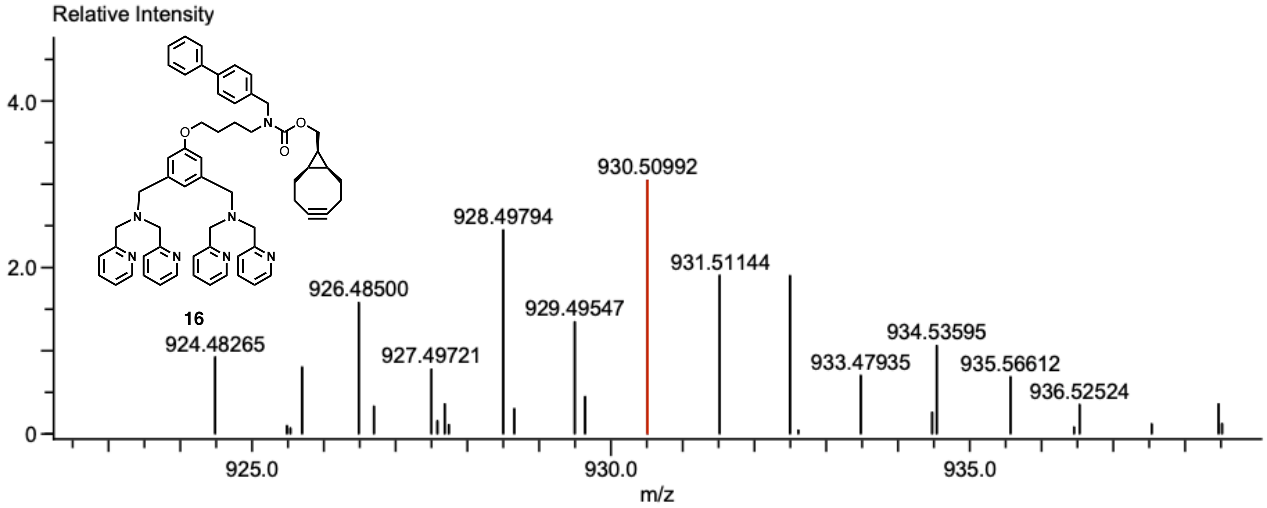

Mass	Intensity	Calc. Mass	Mass Difference [mDa]	Mass Difference [ppm]	Possible Formula
930.50992	4582.65	930.50706	2.86	3.08	$^{12}C_{60}{}^{1}H_{64}{}^{14}N_{7}{}^{16}O_{3}$

Figure S 15. The high res spectra of compound **16**.

Spectra of compound 17.

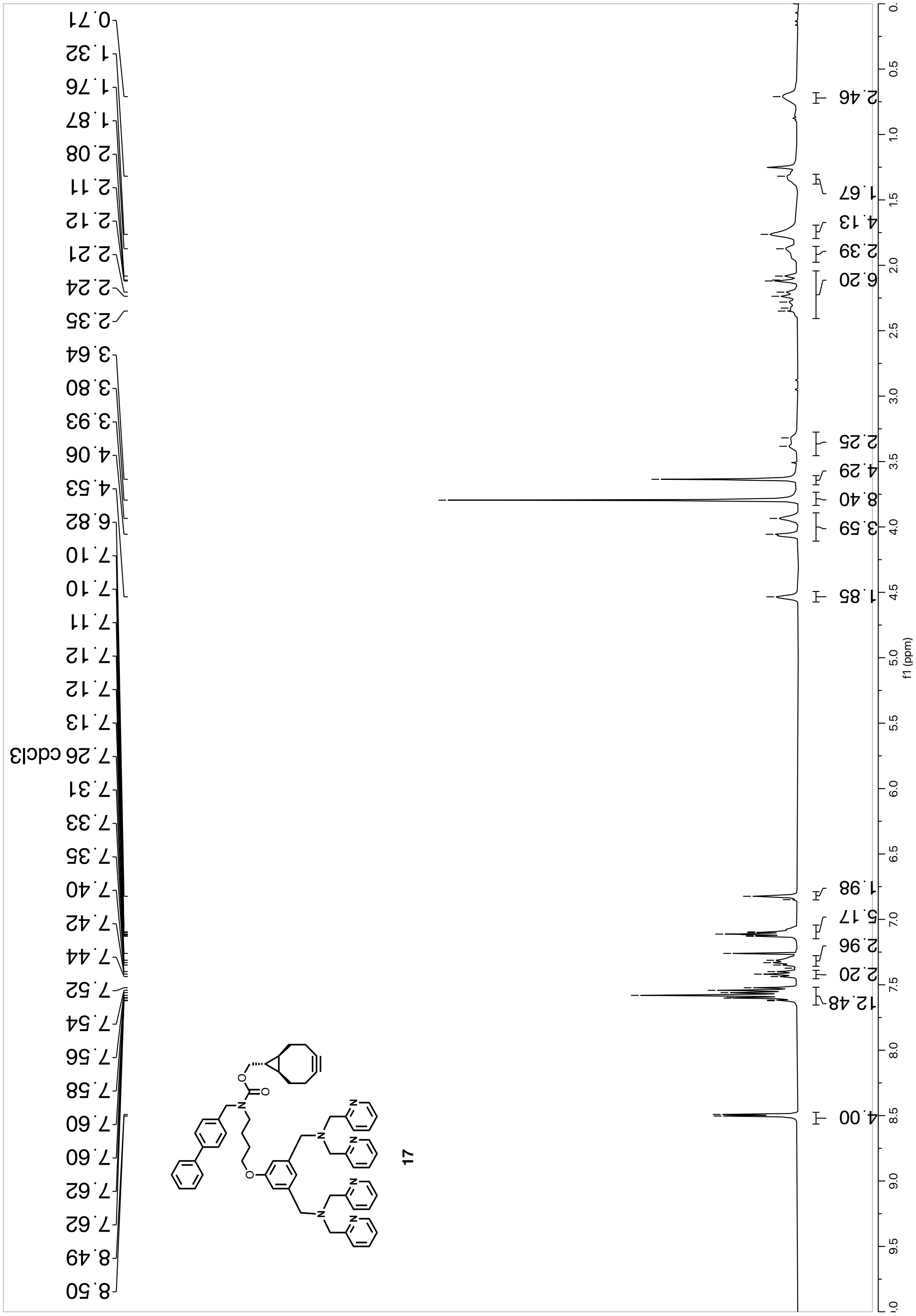

Figure S 16. The ^{1}H NMR of compound **17**.

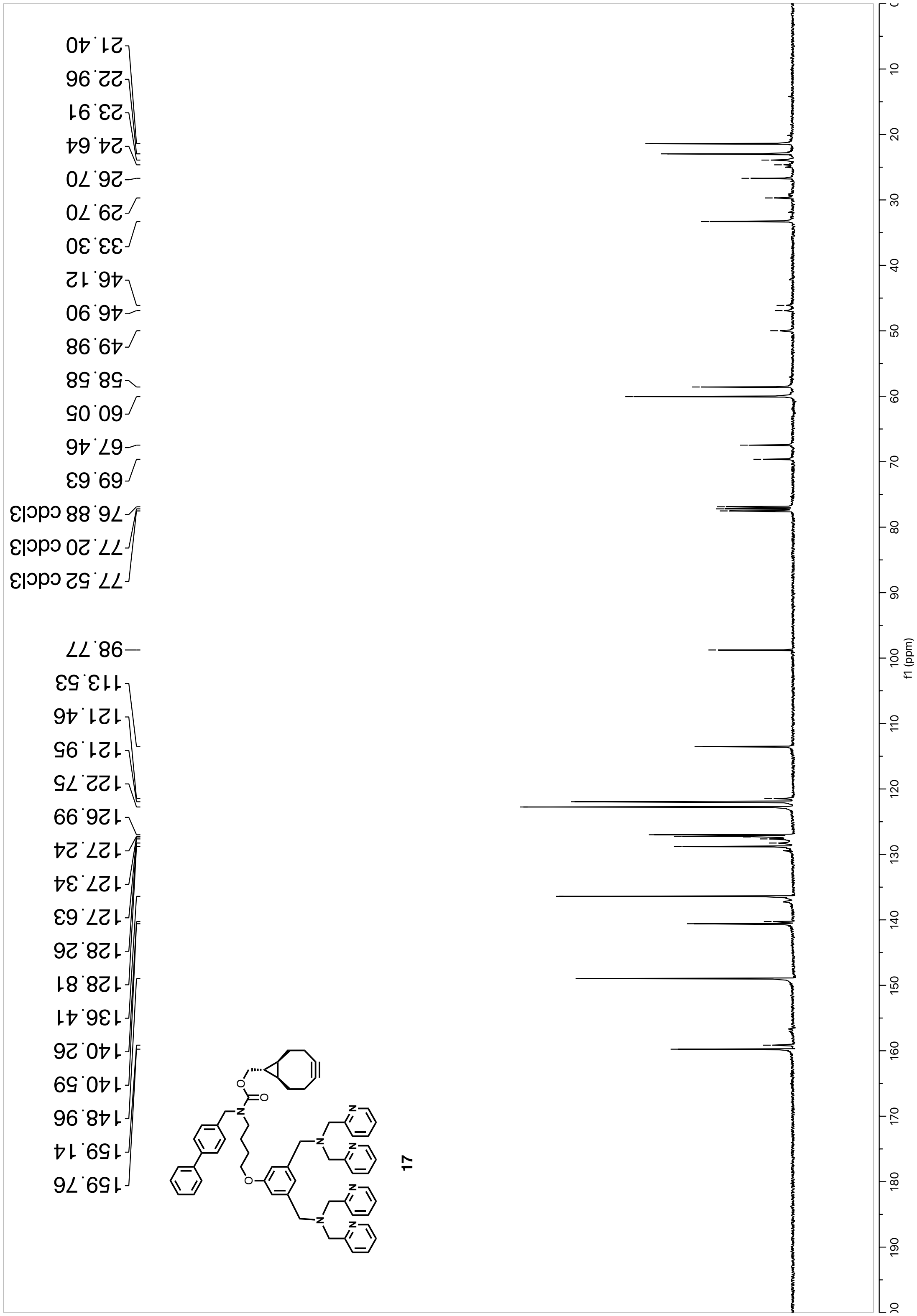

Figure S 17. The ^{13}C NMR of compound **17**.

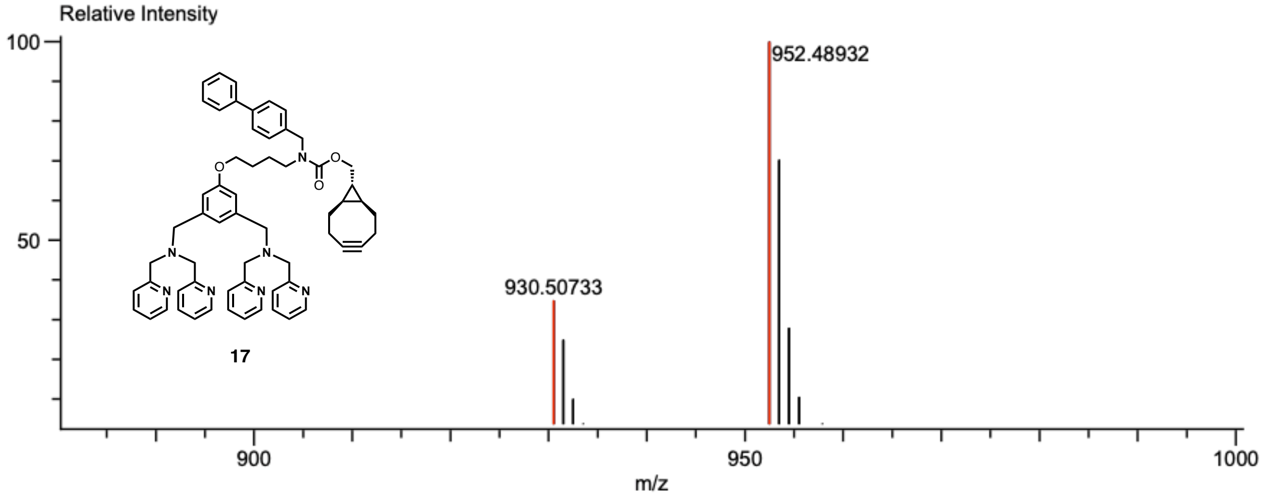

Mass	Intensity	Calc. Mass	Mass Difference [mDa]	Mass Difference [ppm]	Possible Formula
930.50733	588810.18	930.50706	0.27	0.28	$^{12}C_{60}{}^{1}H_{64}{}^{14}N_{7}{}^{16}O_{3}$
952.48932	1701546.20	952.48901	0.31	0.33	$^{12}C_{60}{}^{1}H_{63}{}^{14}N_{7}{}^{23}Na_{1}{}^{16}O_{3}$

Figure S 18. The high res spectra of compound **17**.

Spectra of compound 18.

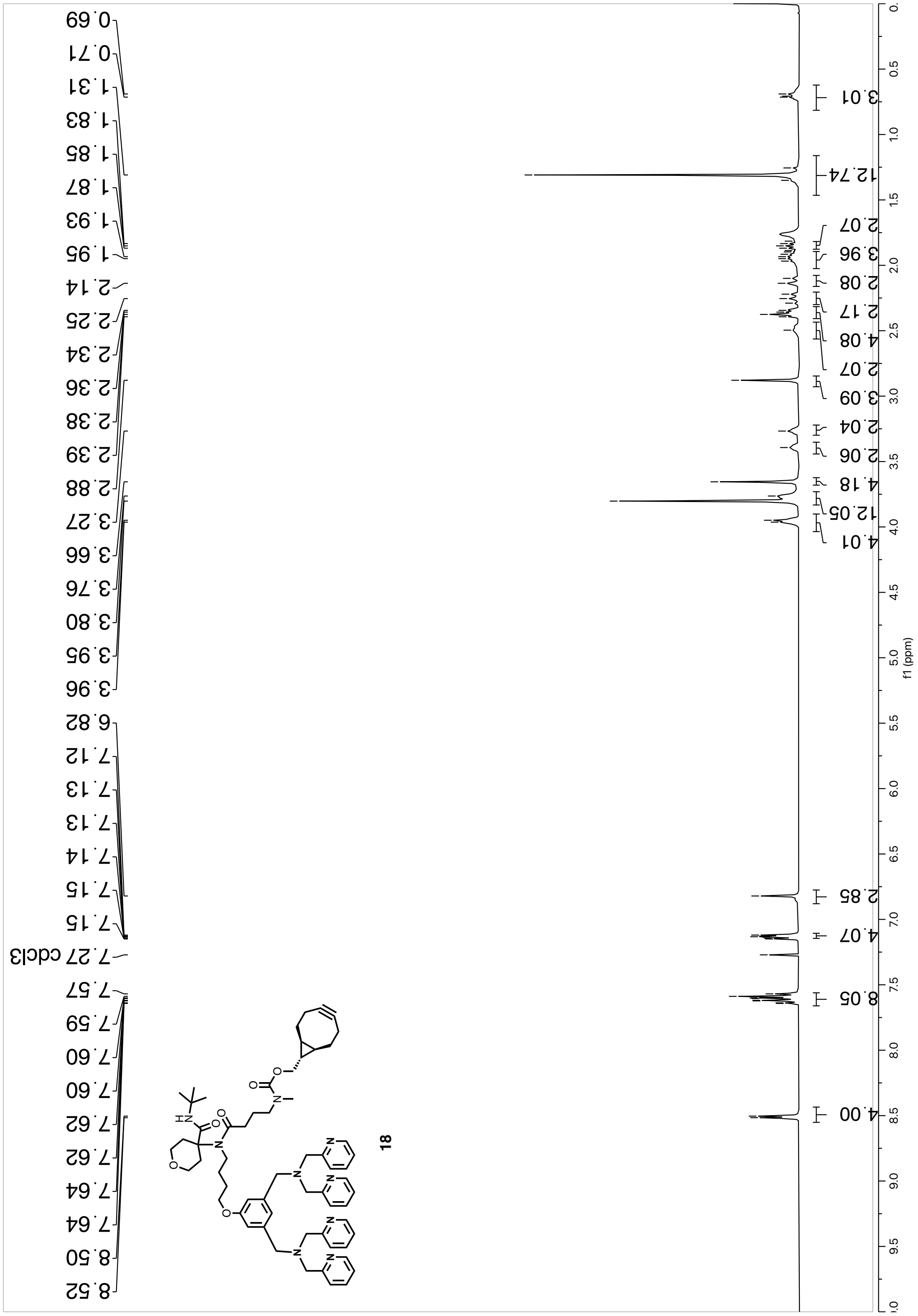

Figure S 19. The ^{1}H NMR of compound **18**.

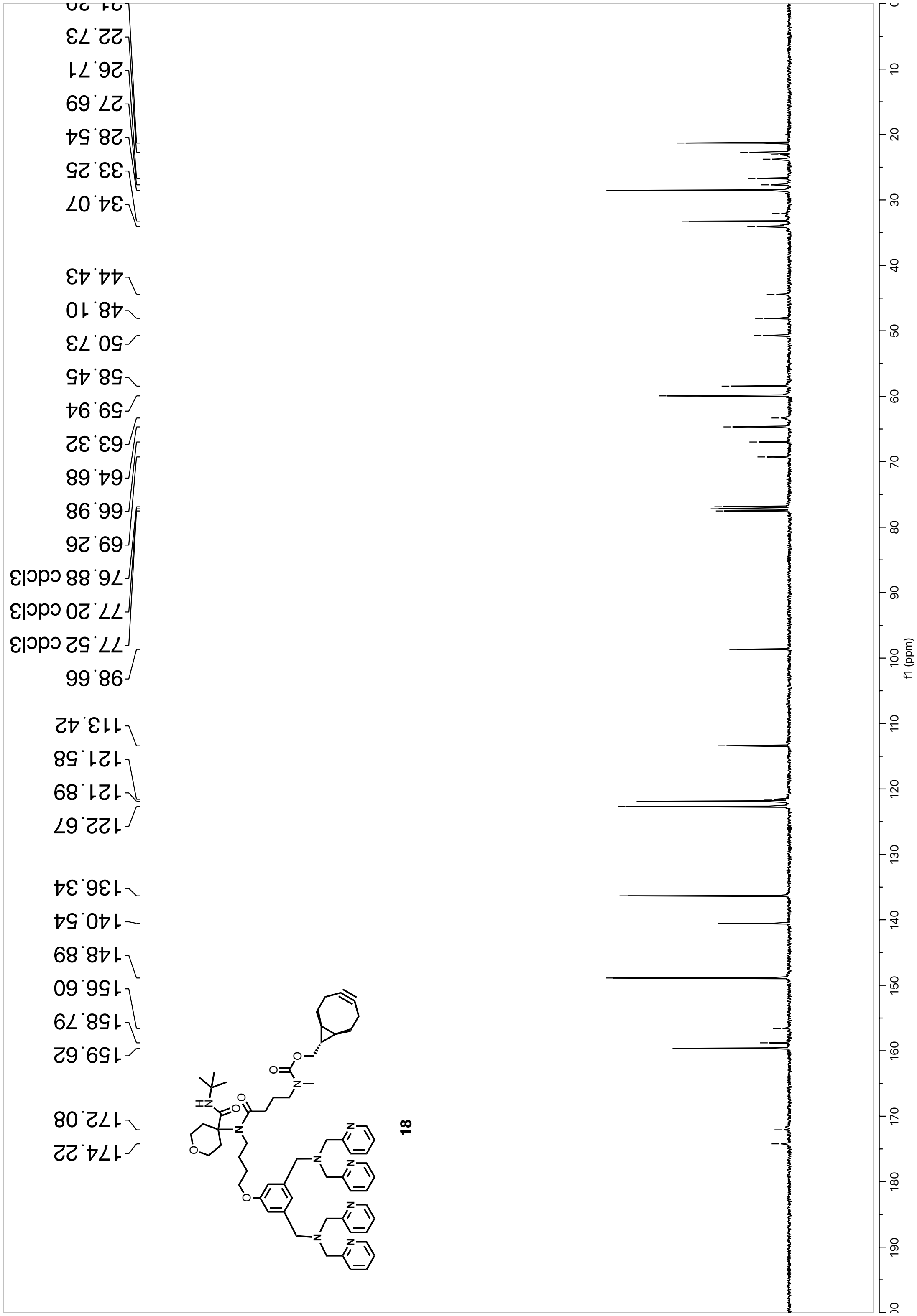

Figure S 20. The ^{13}C NMR of compound **18**.

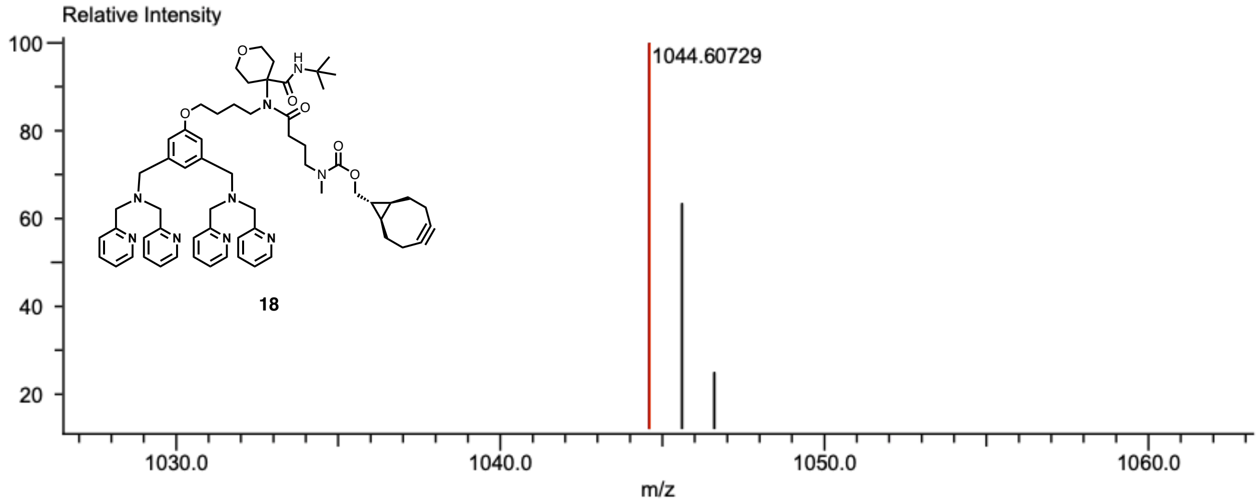

Mass	Intensity	Calc. Mass	Mass Difference [mDa]	Mass Difference [ppm]	Possible Formula
1044.60729	3398.78	1044.60750	-0.22	-0.21	$^{12}C_{62}{}^{1}H_{78}{}^{14}N_{9}{}^{16}O_{6}$

Figure S 21. The high res spectra of compound **18**.

Spectra of compound 19.

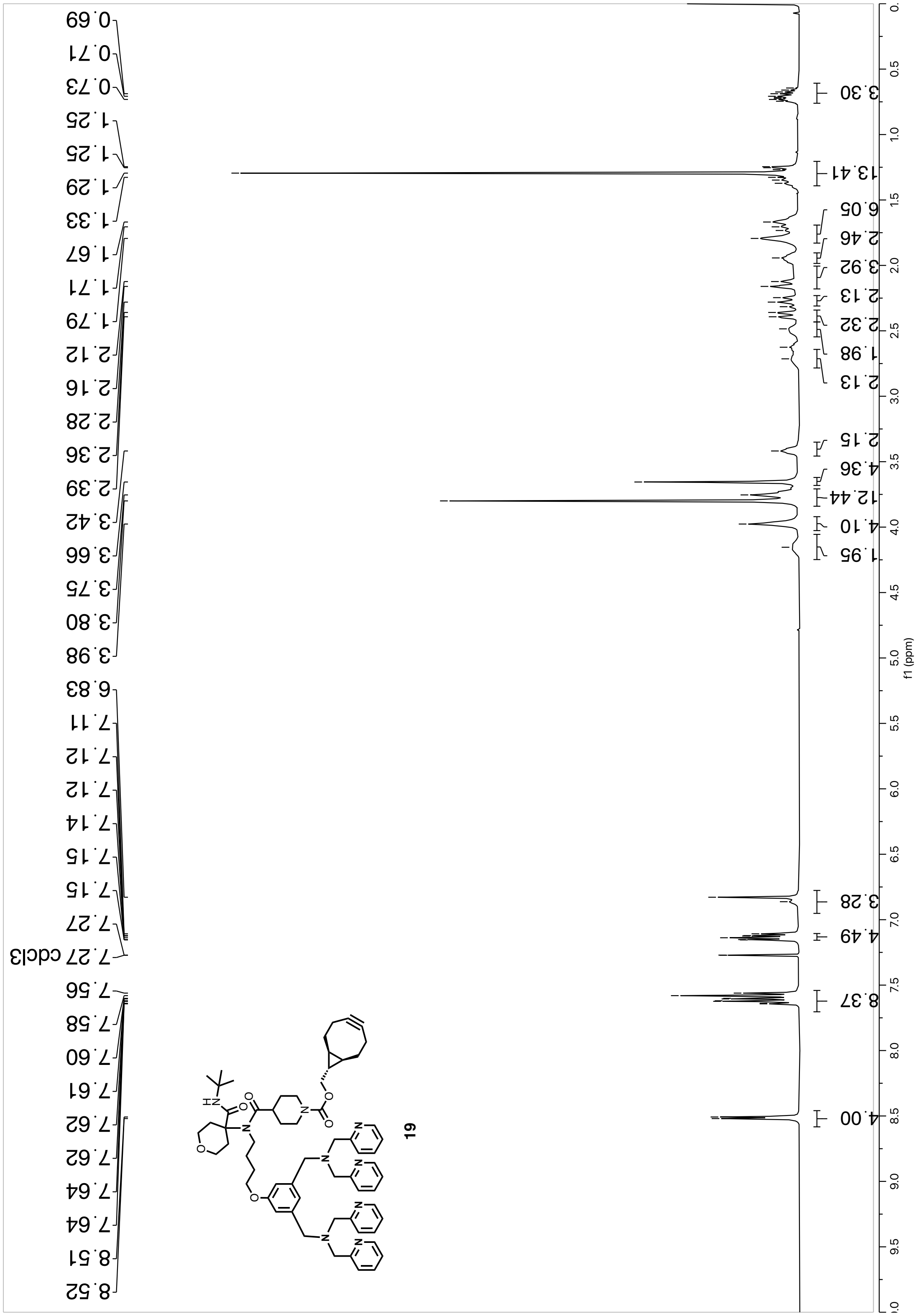

Figure S 22. The ^{1}H NMR of compound **19**.

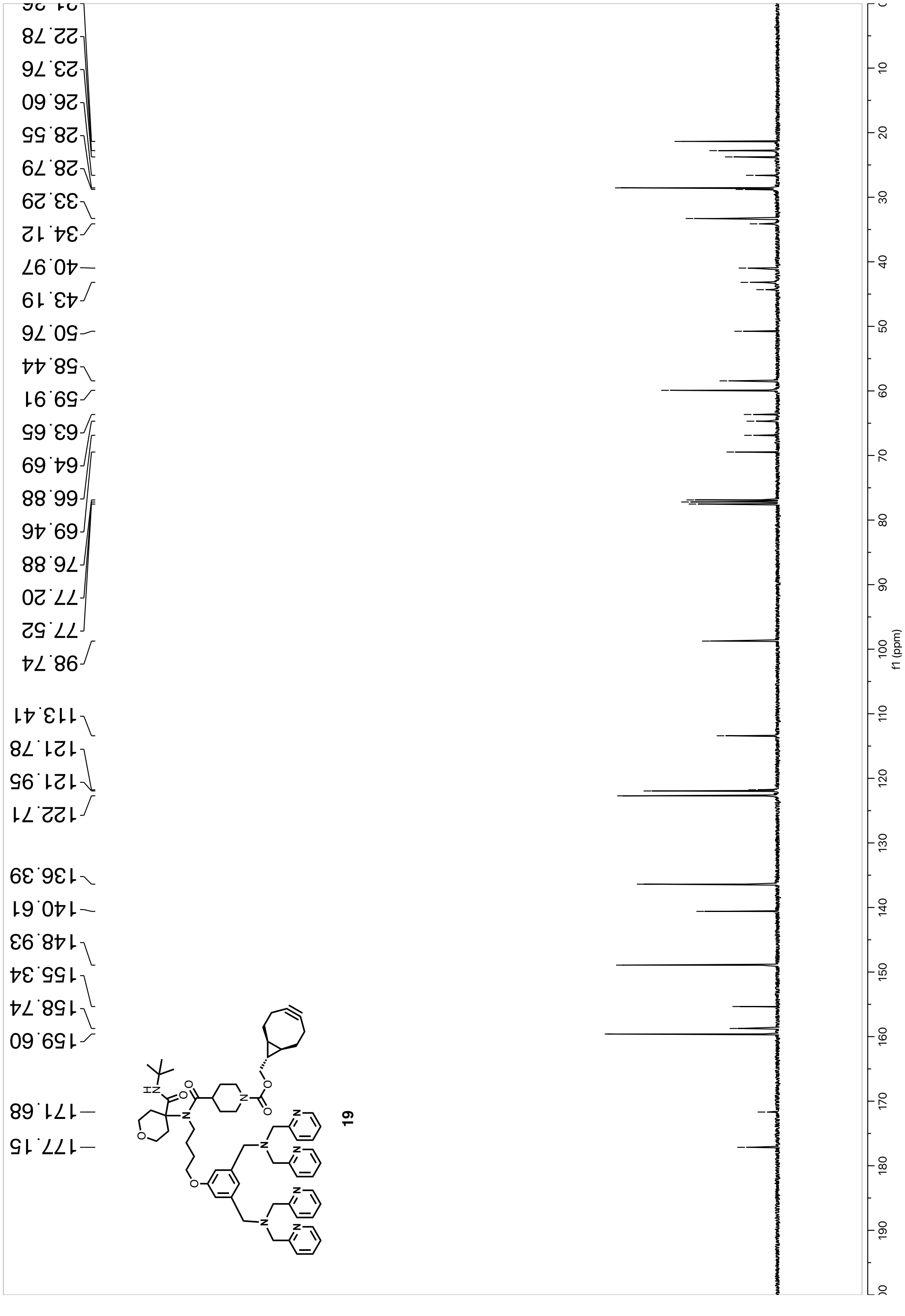

Figure S 23. The ^{13}C NMR of compound **19**.

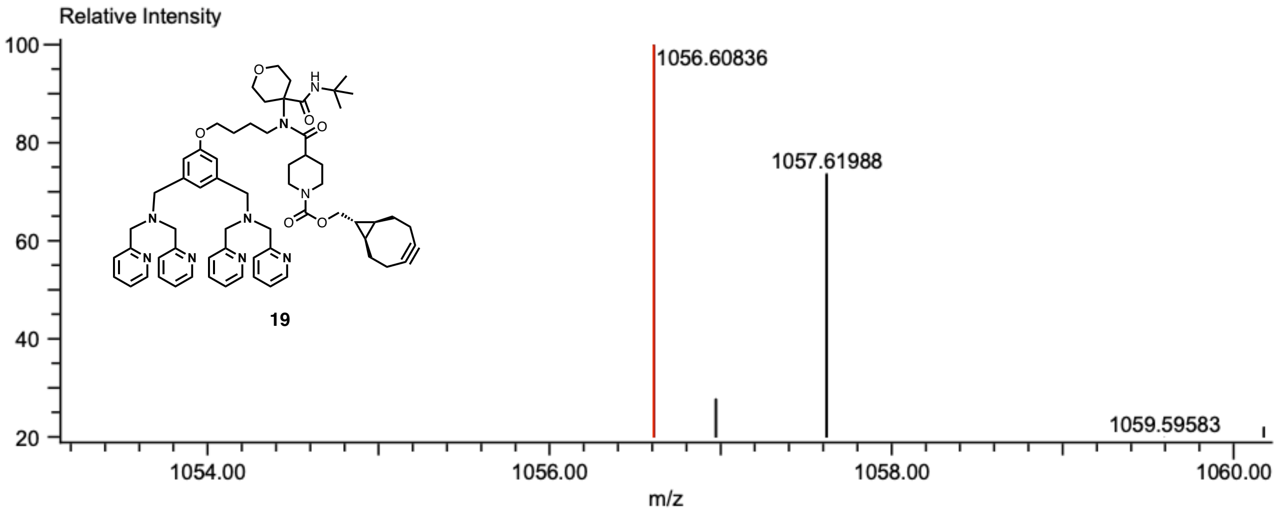

Mass	Intensity	Calc. Mass	Mass Difference [mDa]	Mass Difference [ppm]	Possible Formula
1056.60836	238.84	1056.60750	0.85	0.81	$^{12}C_{63}{}^{1}H_{78}{}^{14}N_{9}{}^{16}O_{6}$

Figure S 24. The high res spectra of compound **19**.

Spectra of compound 20.

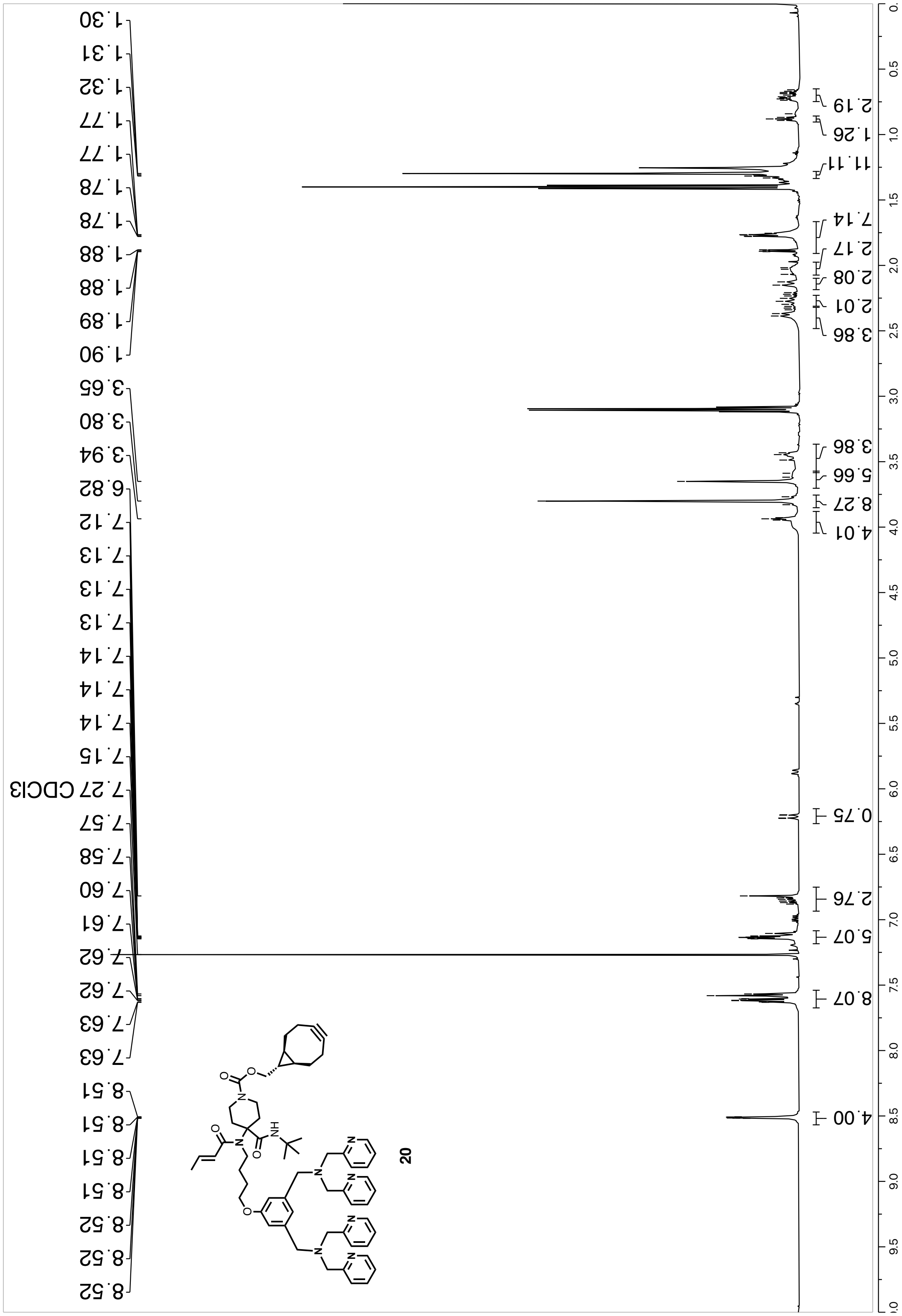

Figure S 25. The ^{1}H NMR of compound **20**.

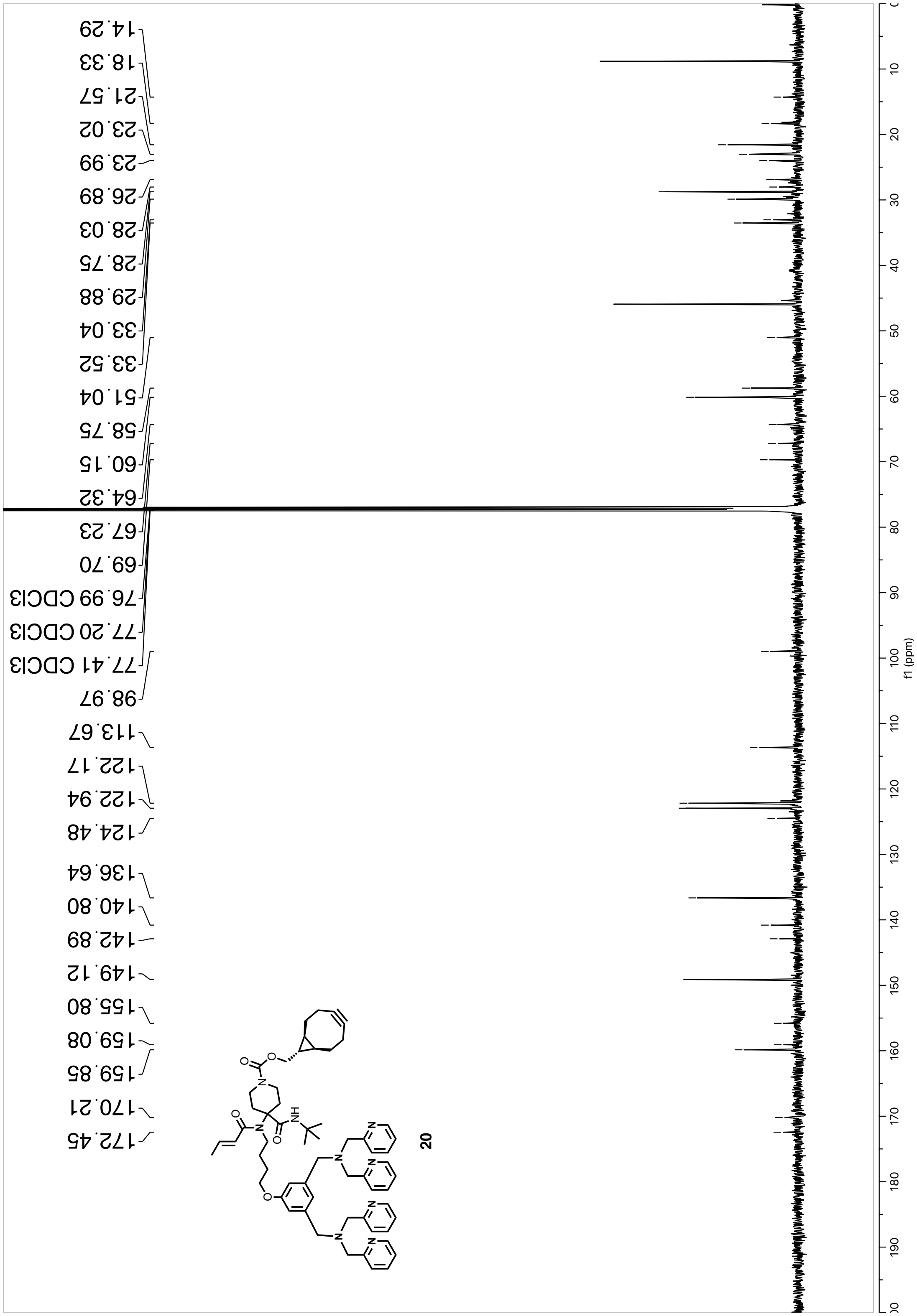

Figure S 26. The ^{13}C NMR of compound **20**.

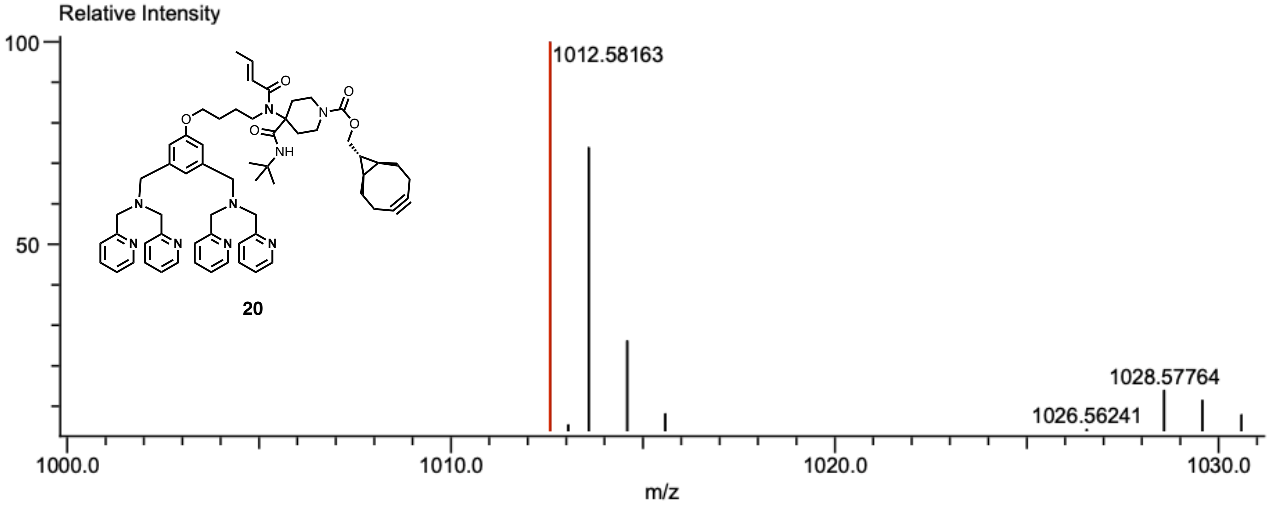

Mass	Intensity	Calc. Mass	Mass Difference [mDa]	Mass Difference [ppm]	Possible Formula
1012.58163	16109.72	1012.58129	0.34	0.34	$^{12}C_{61}{}^{1}H_{74}{}^{14}N_{9}{}^{16}O_{5}$

Figure S 27. The high res spectra of compound **20**.

Spectra of compound 21.

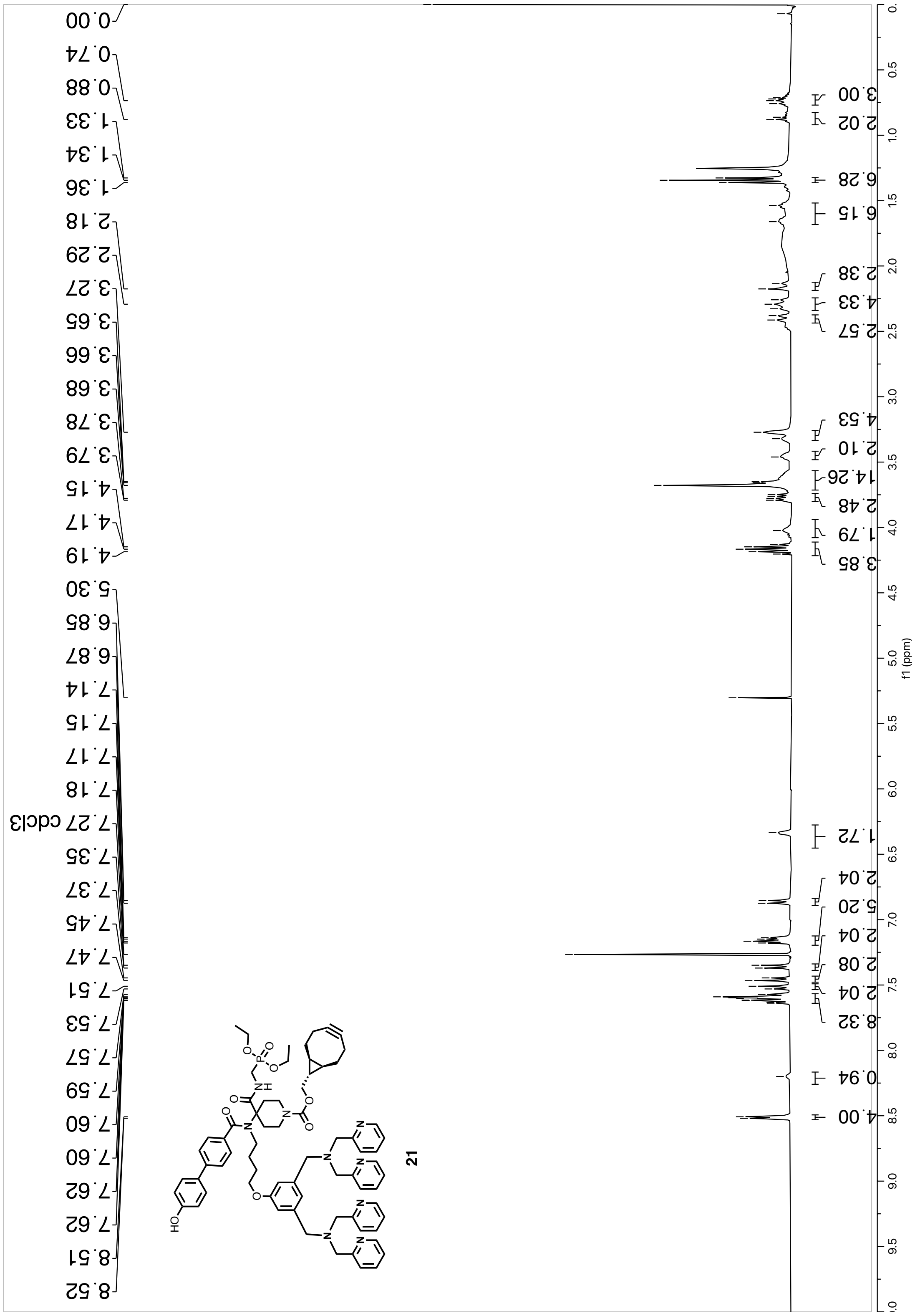

Figure S 28. The ^{1}H NMR of compound **21**.

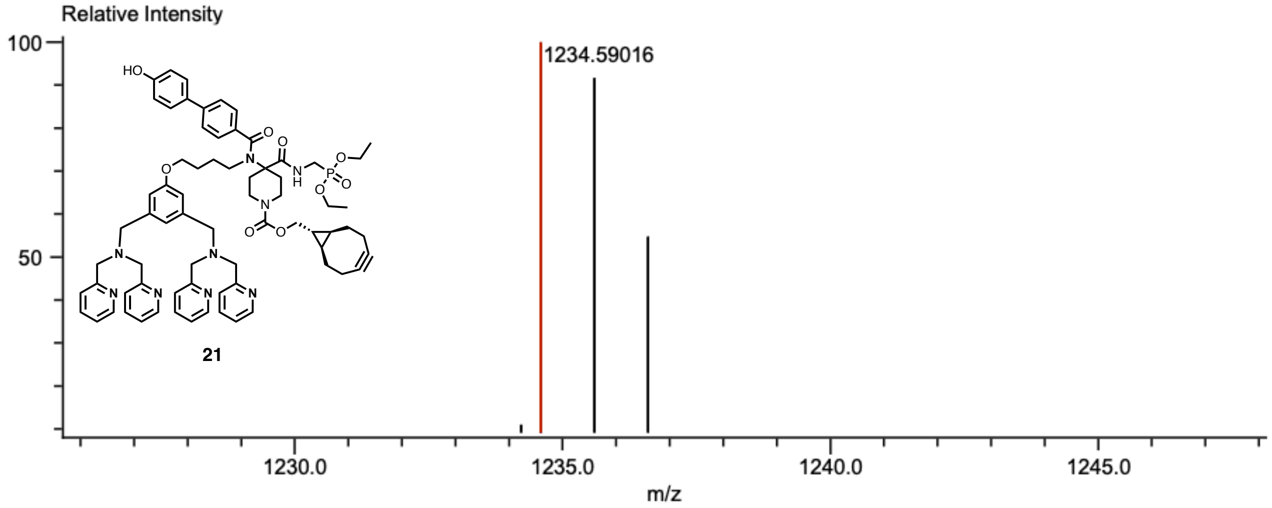

Mass	Intensity	Calc. Mass	Mass Difference [mDa]	Mass Difference [ppm]	Possible Formula
1234.59016	1136.05	1234.58948	0.68	0.55	$^{12}C_{71}{}^{1}H_{81}{}^{14}N_{9}{}^{16}O_{9}{}^{31}P_{1}$

Figure S 29. The high res spectra of compound **21**.

Spectra of precursor of conjugate 1.

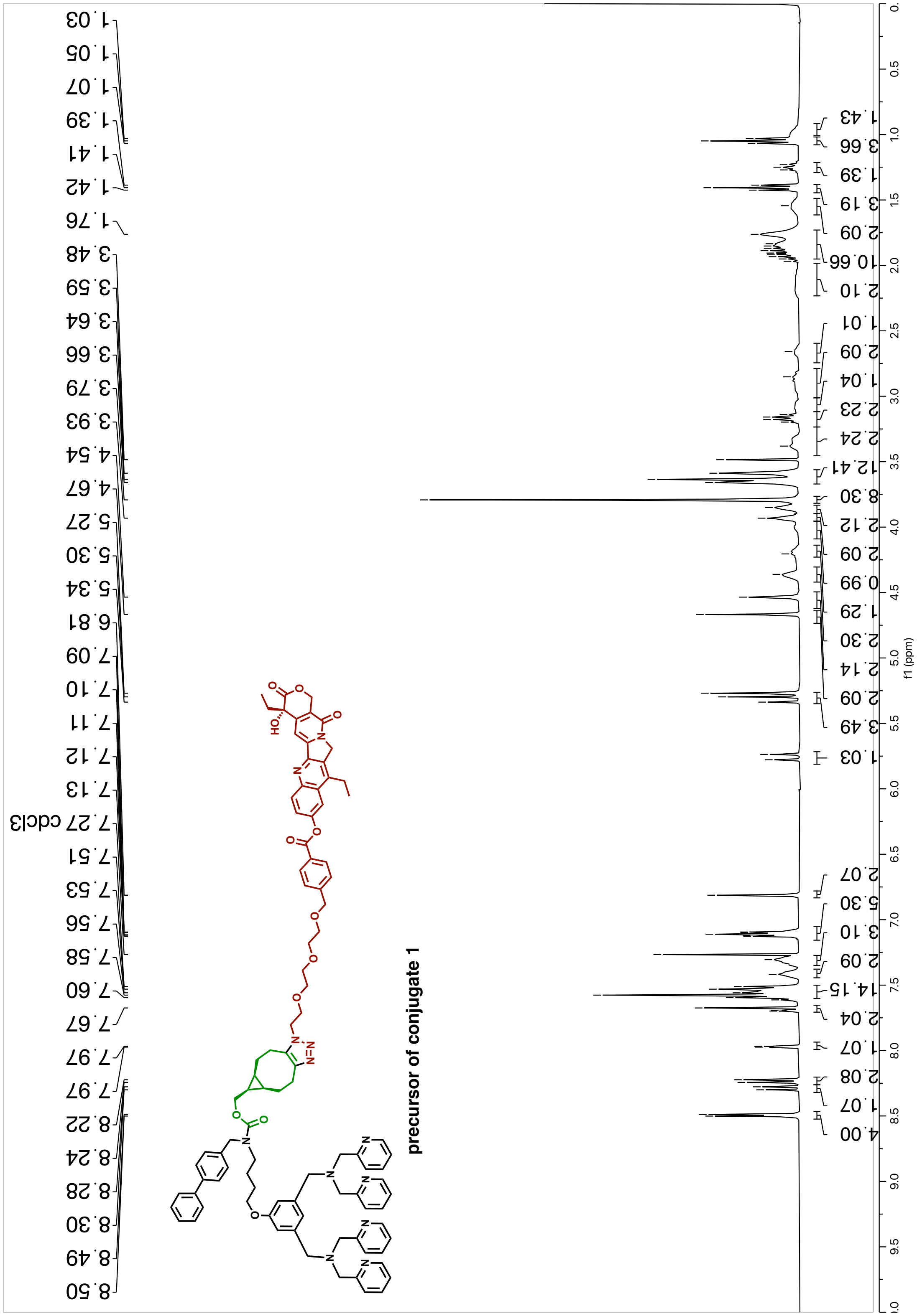

Figure S 30. The ^{1}H NMR of precursor of conjugate **1**.

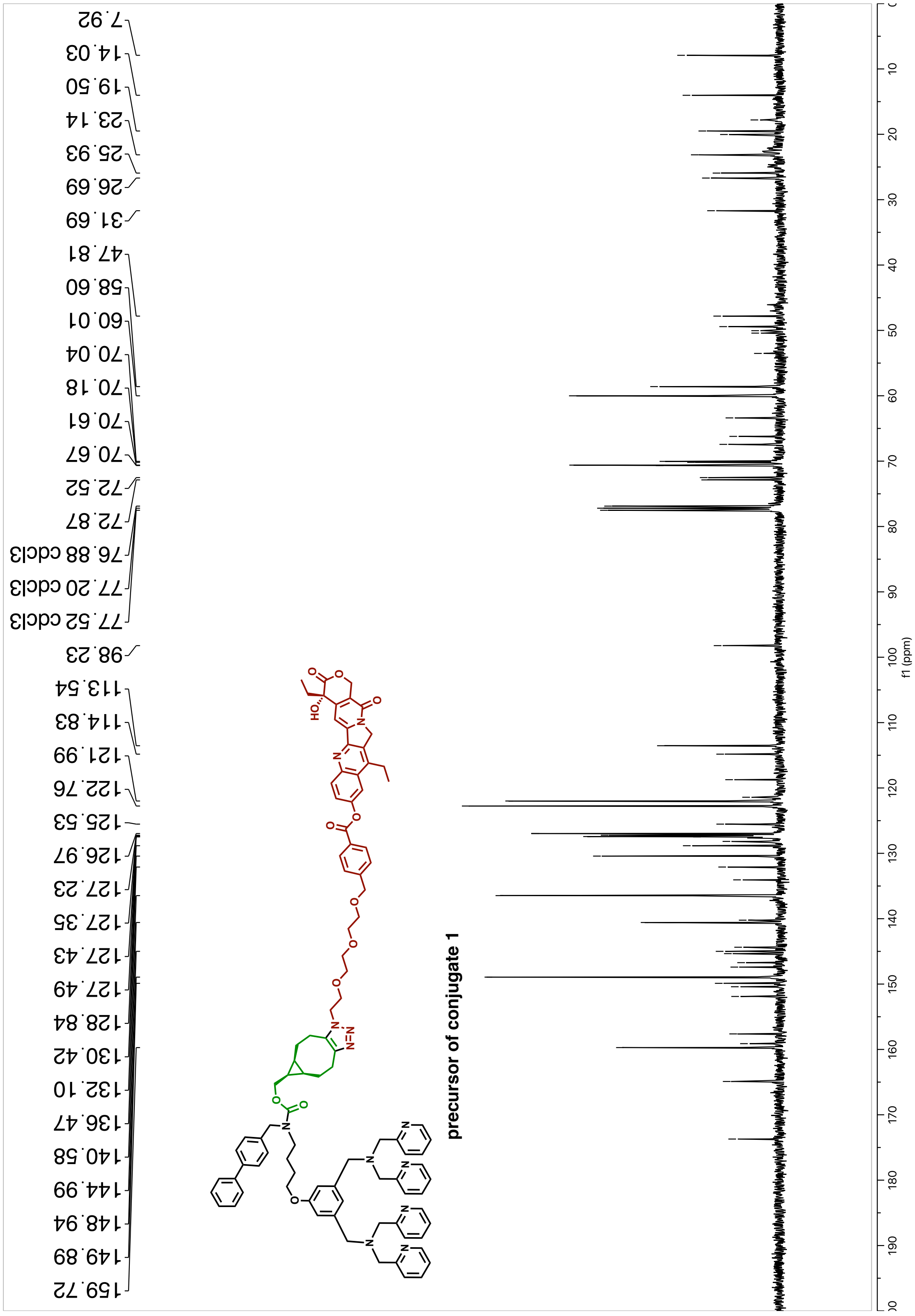

Figure S 31. The ^{13}C NMR of precursor of conjugate **1**.

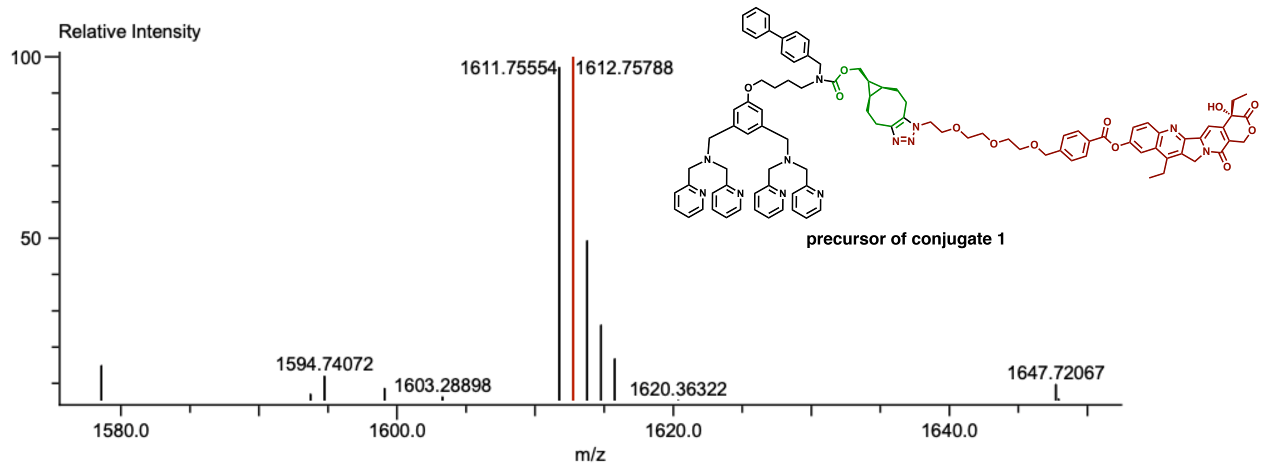

Mass	Intensity	Calc. Mass	Mass Difference [mDa]	Mass Difference [ppm]	Possible Formula
1612.75788	7201.96	1612.75836	-0.48	-0.30	$^{12}C_{96}{}^{1}H_{100}{}^{14}N_{12}{}^{16}O_{12}$

Figure S 32. The high res spectra of precursors of conjugate**1**.

Spectra of precursor of conjugate 2.

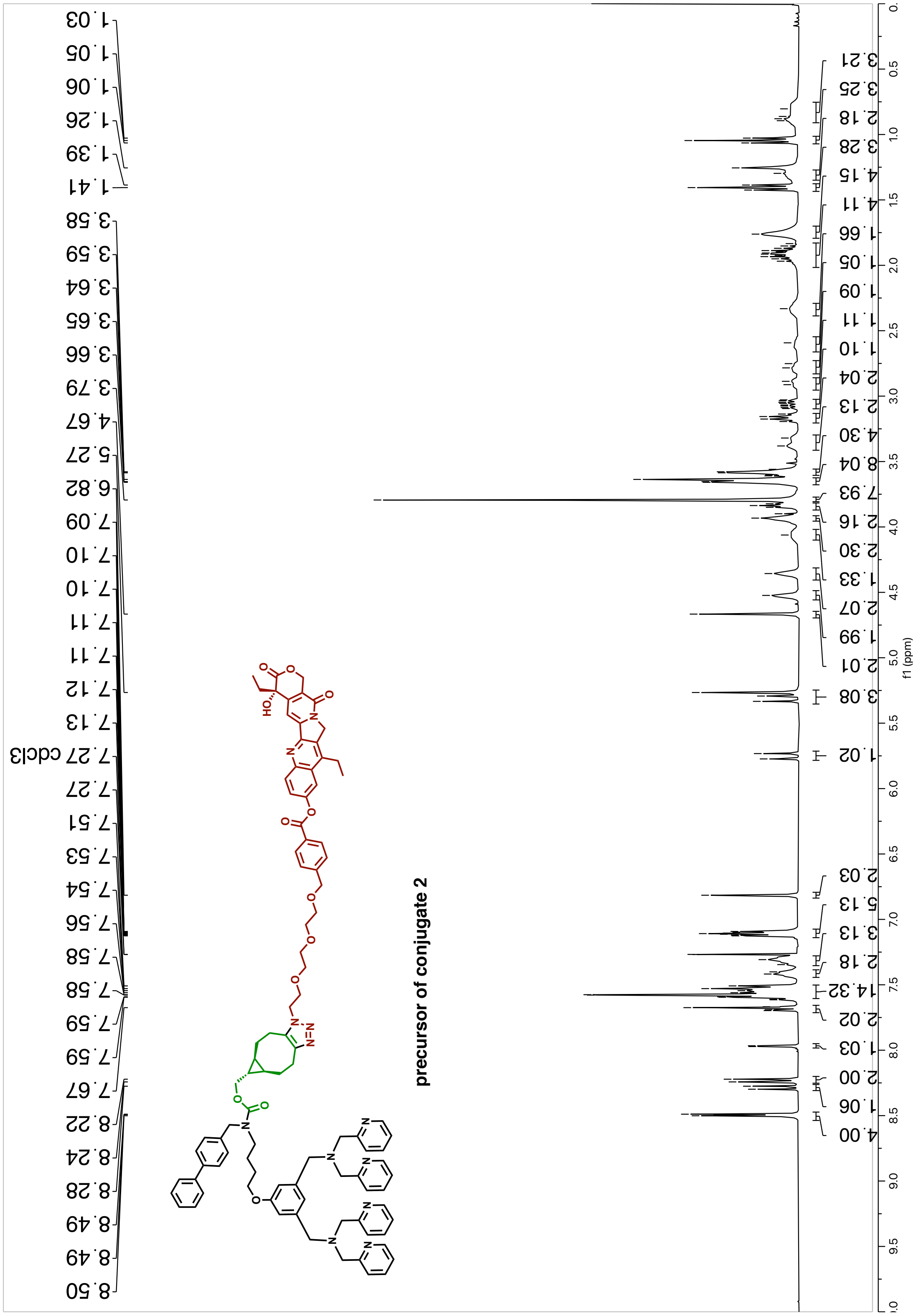

Figure S 33. The ^{1}H NMR of precursor of conjugate **2**.

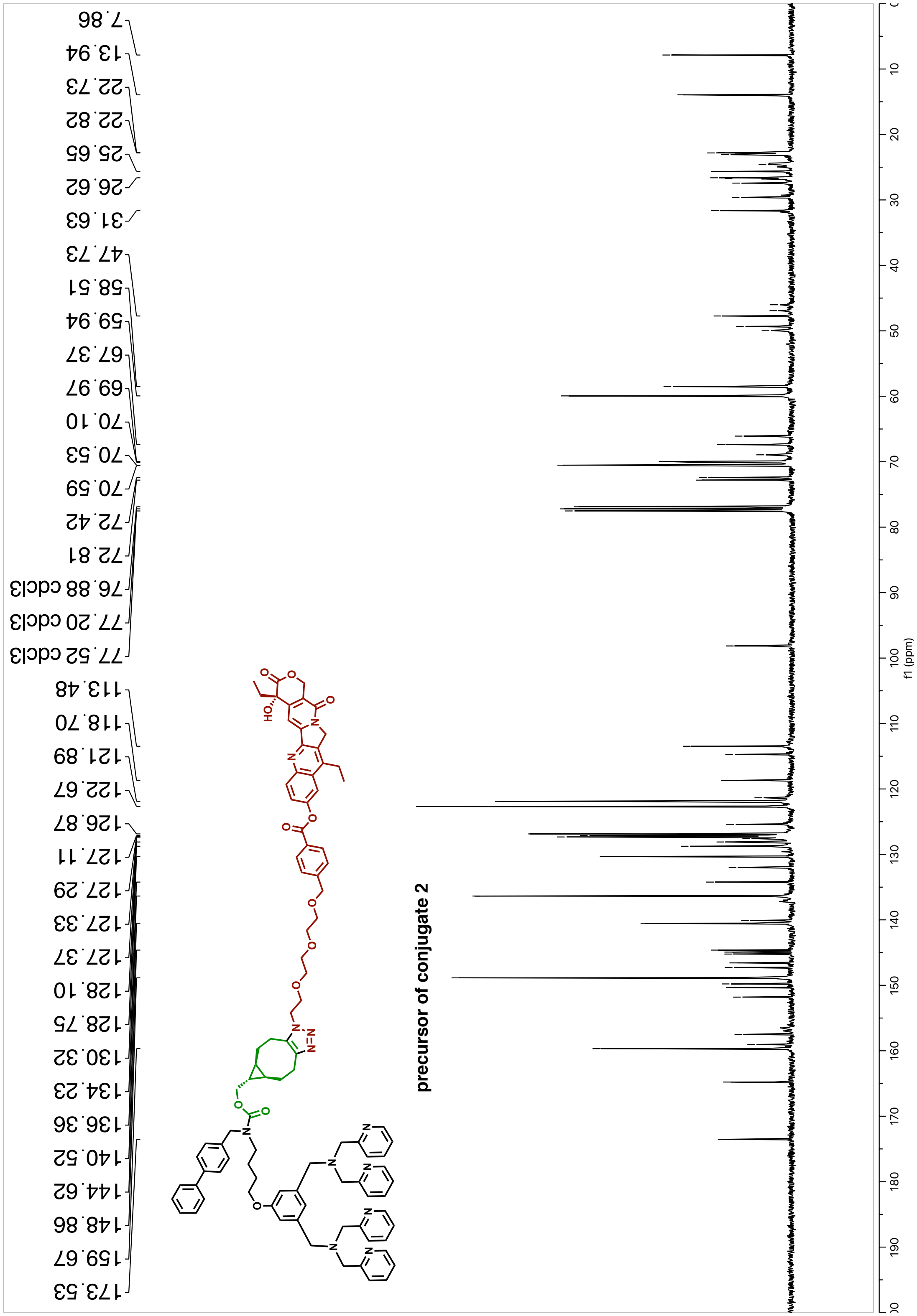

Figure S 34. The ^{13}C NMR of precursors of conjugate **2**.

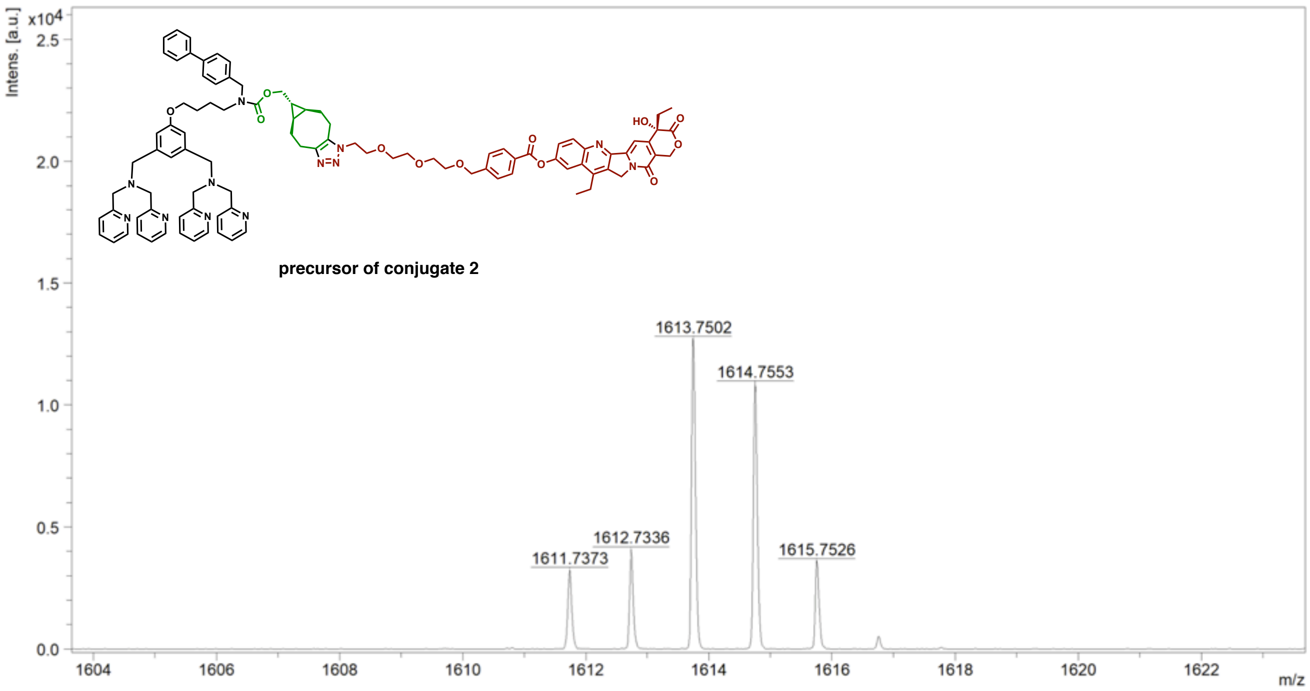

Figure S 35. The high res spectra of precursors of conjugate **2**.

Spectra of precursor conjugate 3.

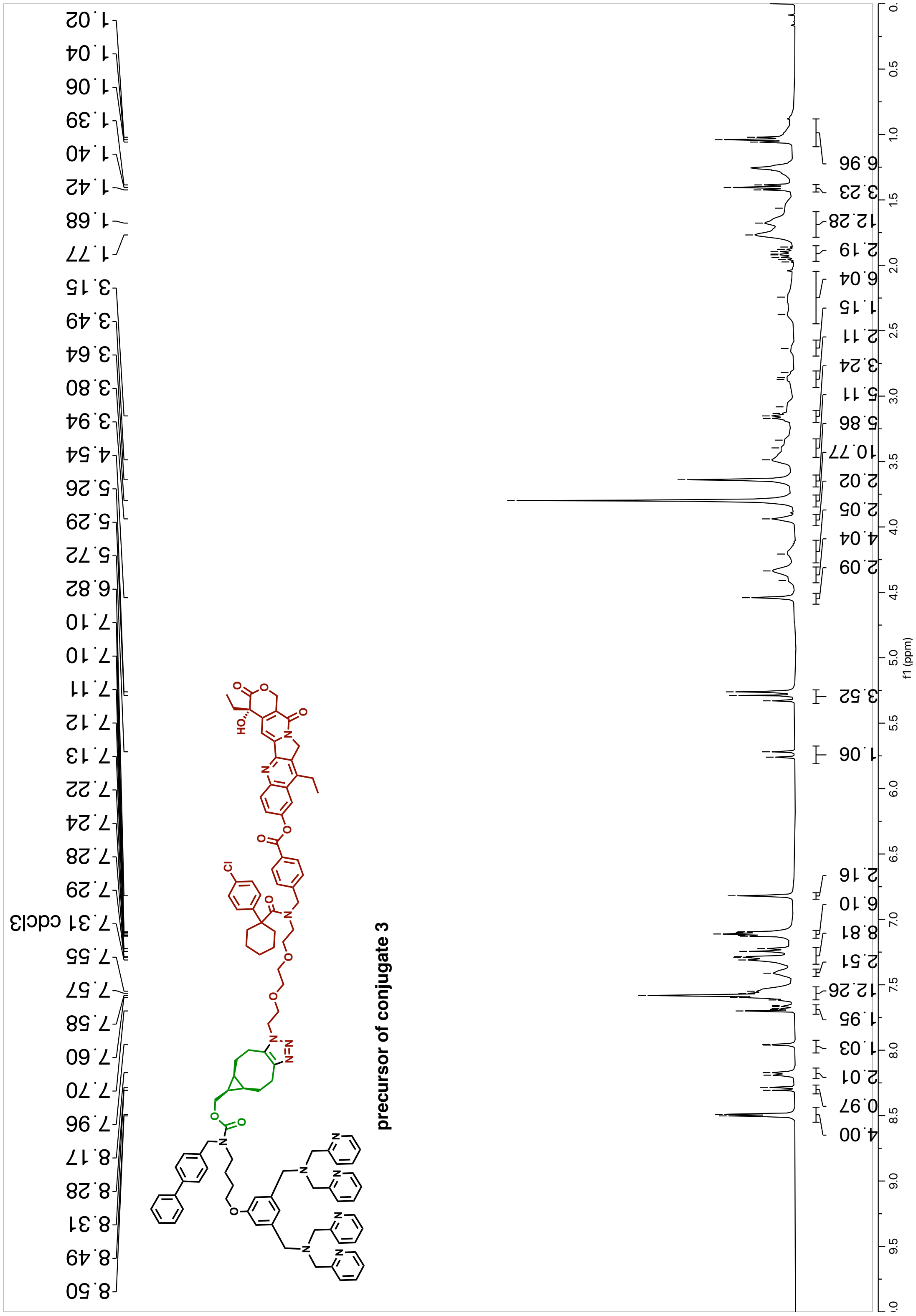

Figure S 36. The ^{1}H NMR of precursor of conjugate **3**.

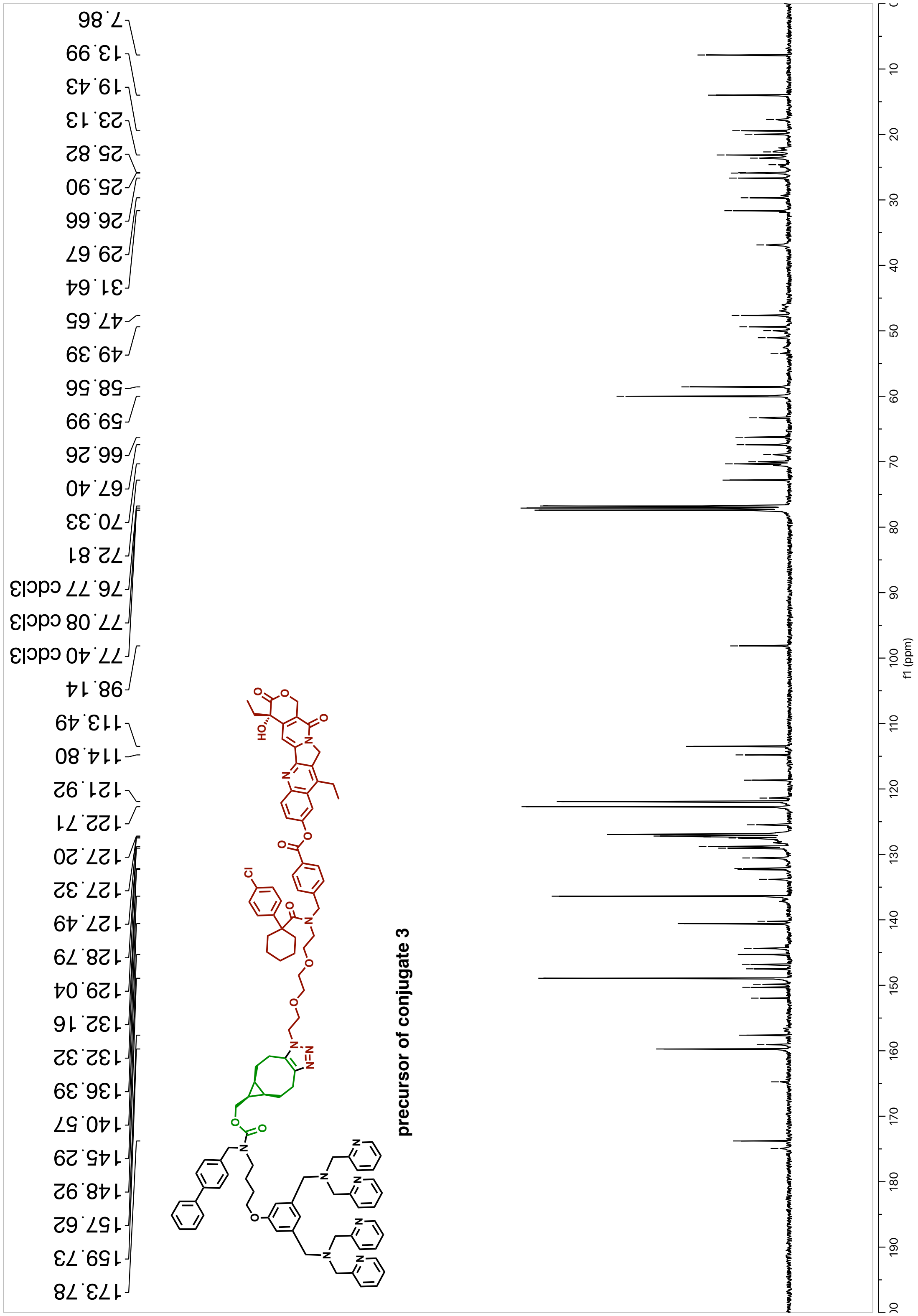

Figure S 37. The ^{13}C NMR of precursorsof conjugate **3**.

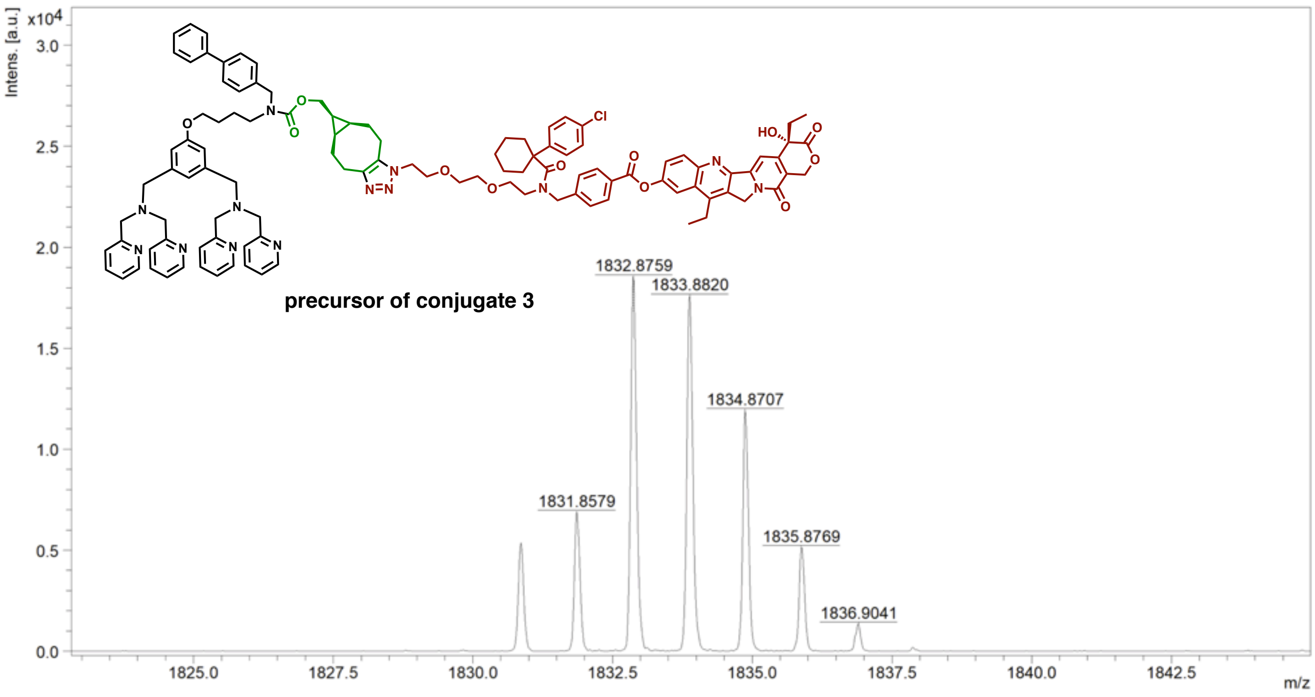

Figure S 38. The high res spectra of precursors of conjugate **3**.

Spectra of precursor of conjugate 5.

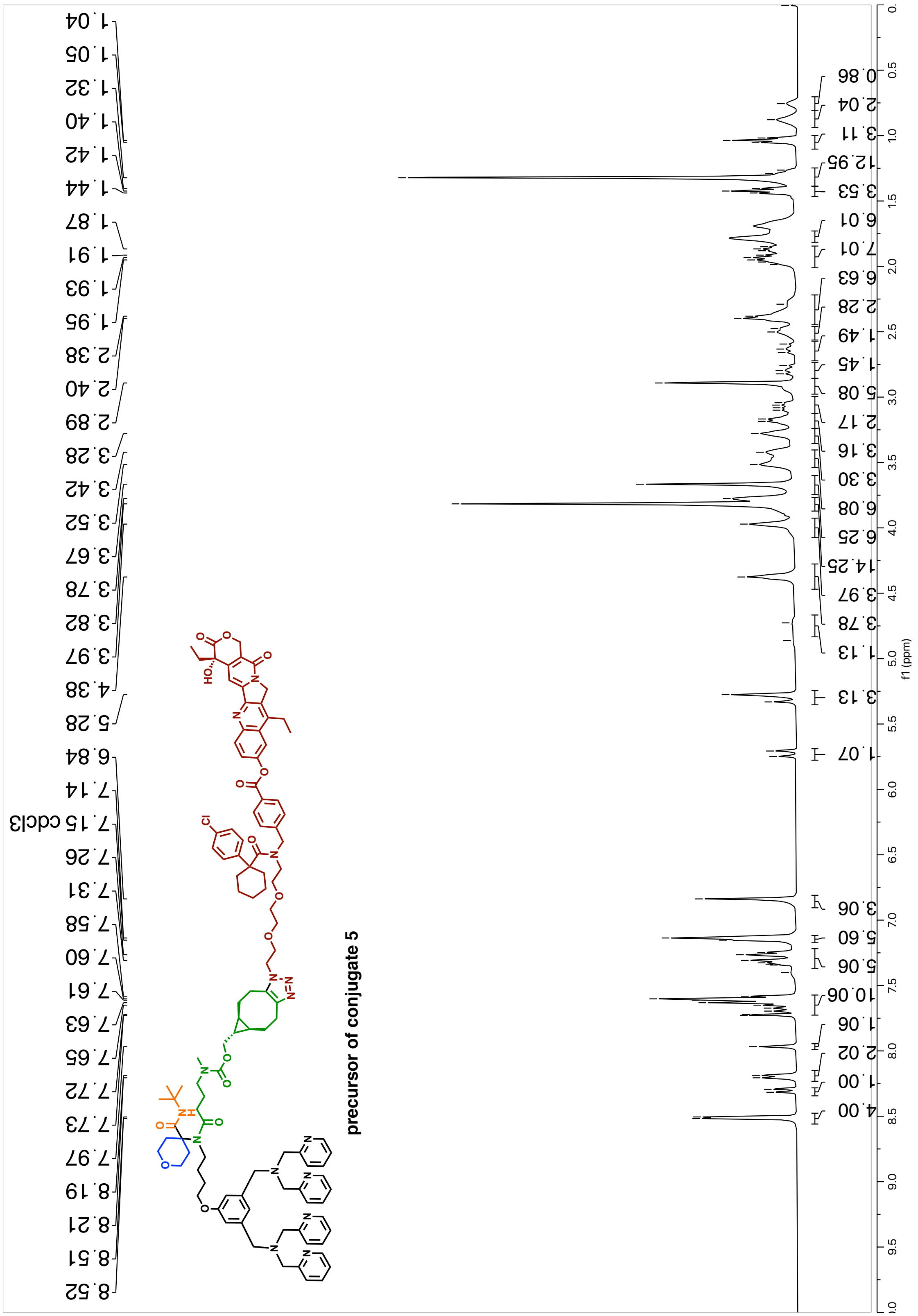

Figure S 39. The ^{1}H NMR of precursor of conjugate **5**.

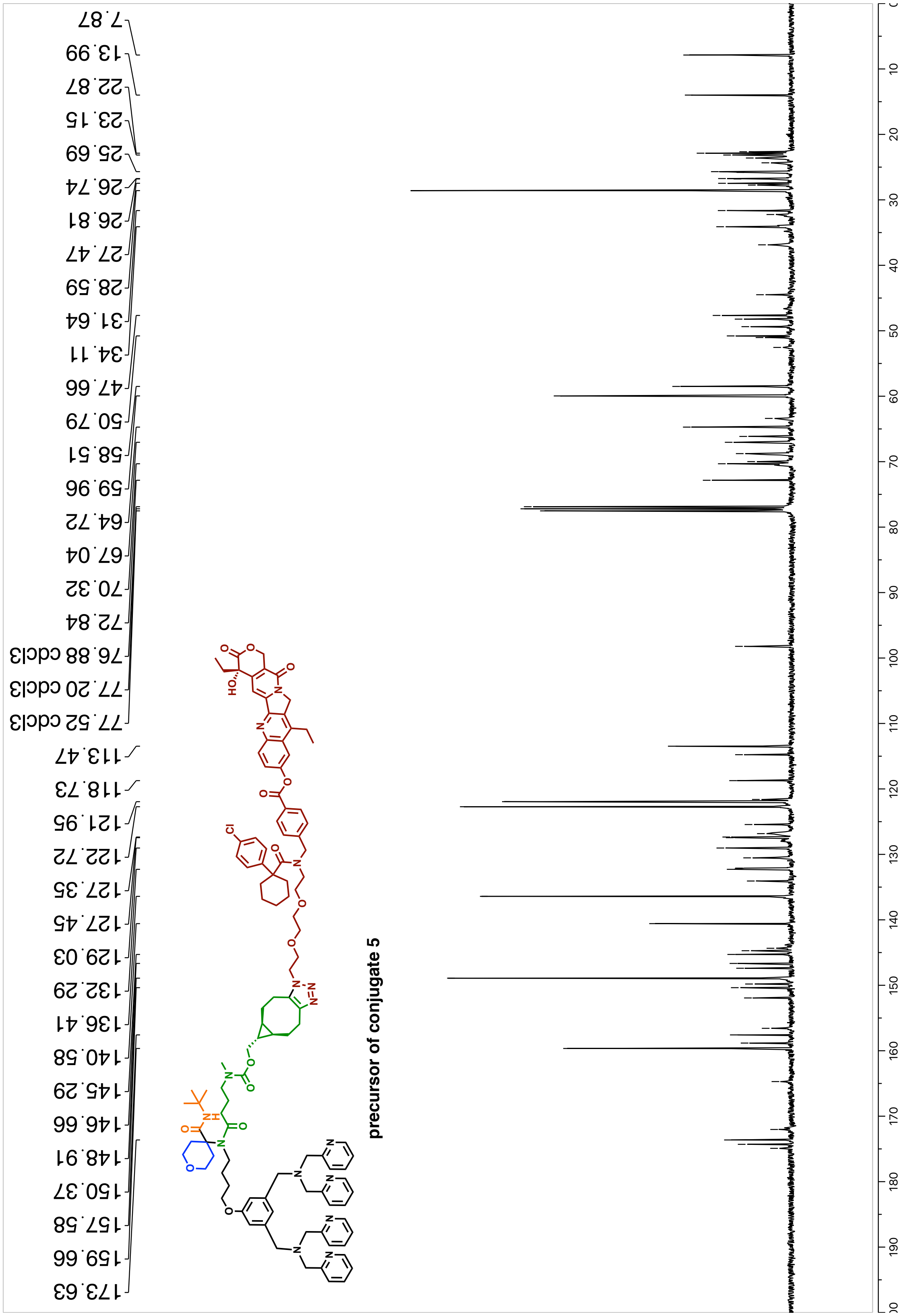

Figure S 40. The ^{13}C NMR of precursor of conjugate **5**.

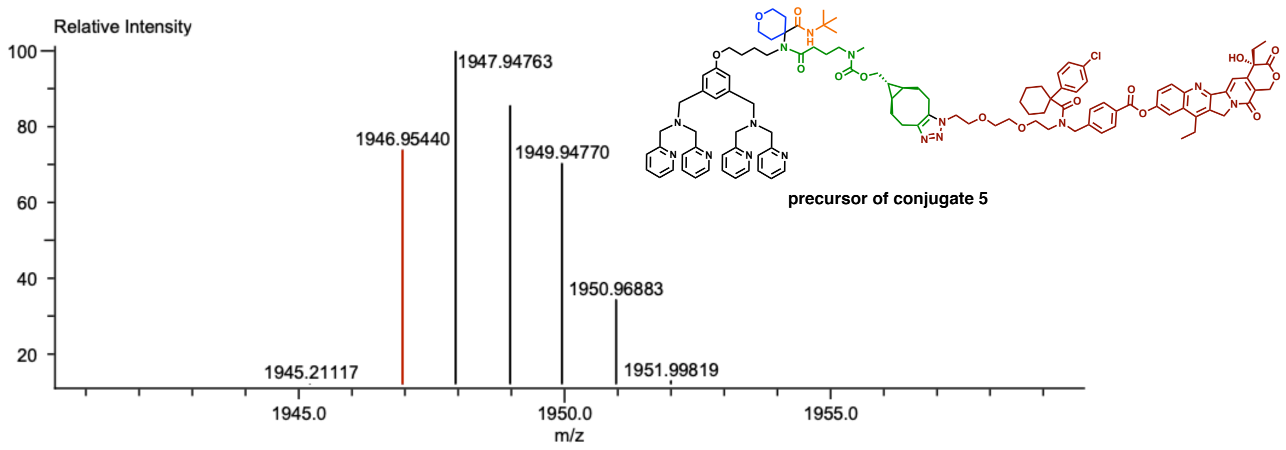

Mass	Intensity	Calc. Mass	Mass Difference [mDa]	Mass Difference [ppm]	Possible Formula
1946.95440	3220.36	1946.94811	6.29	3.23	$^{12}C_{111}{}^{1}H_{129}{}^{35}Cl_{1}{}^{14}N_{15}{}^{16}O_{15}$

Figure S 41. The high res spectra of precursor of conjugate **5**.

Spectra of precursor conjugate 6.

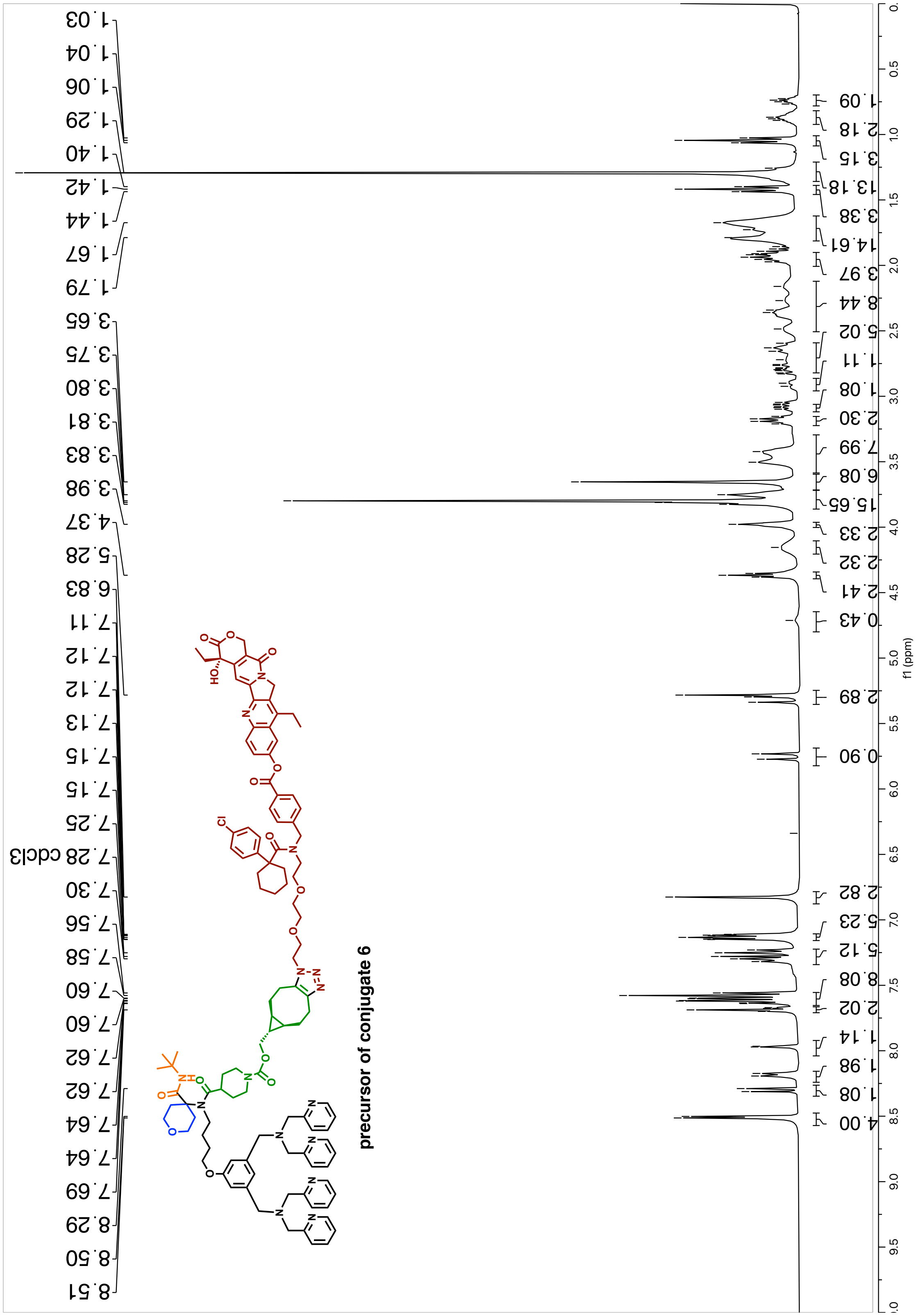

Figure S 42. The ^{1}H NMR of precursor of conjugate **6**.

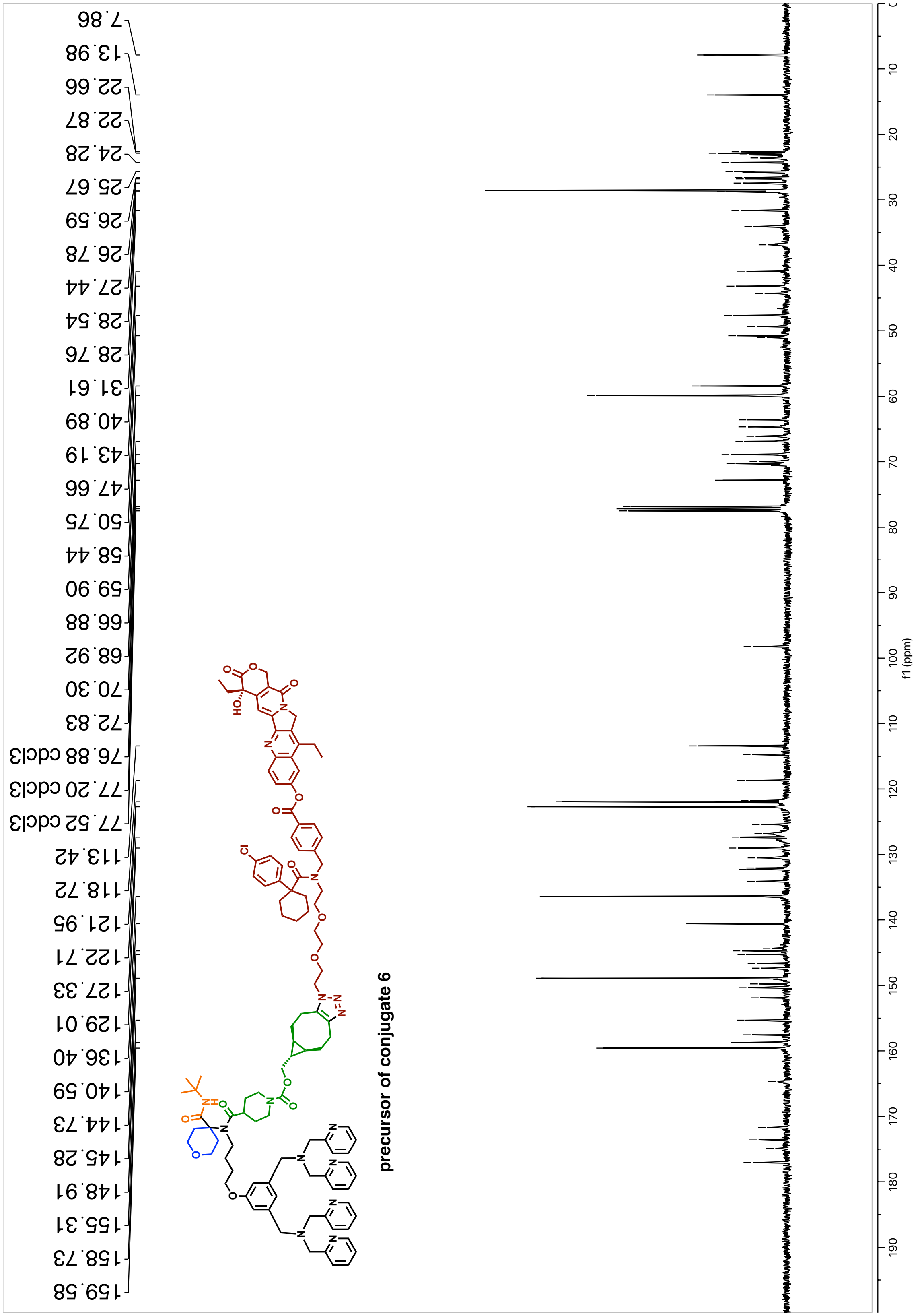

Figure S 43. The ^{13}C NMR of precursor of conjugate **6**.

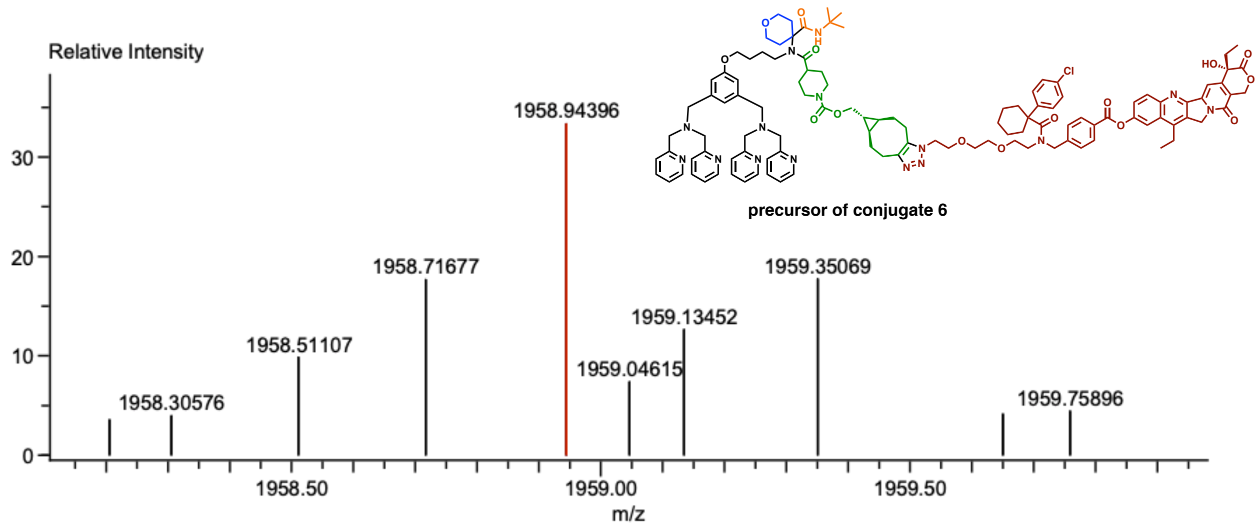

Mass	Intensity	Calc. Mass	Mass Difference [mDa]	Mass Difference [ppm]	Possible Formula
1958.94396	439.32	1958.94811	-4.14	-2.11	$^{12}C_{112}{}^{1}H_{129}{}^{35}Cl_{1}{}^{14}N_{15}{}^{16}O_{15}$

Figure S 44. The high res spectra of precursor of conjugate **6**.

Spectra of precursor of conjugate 7.

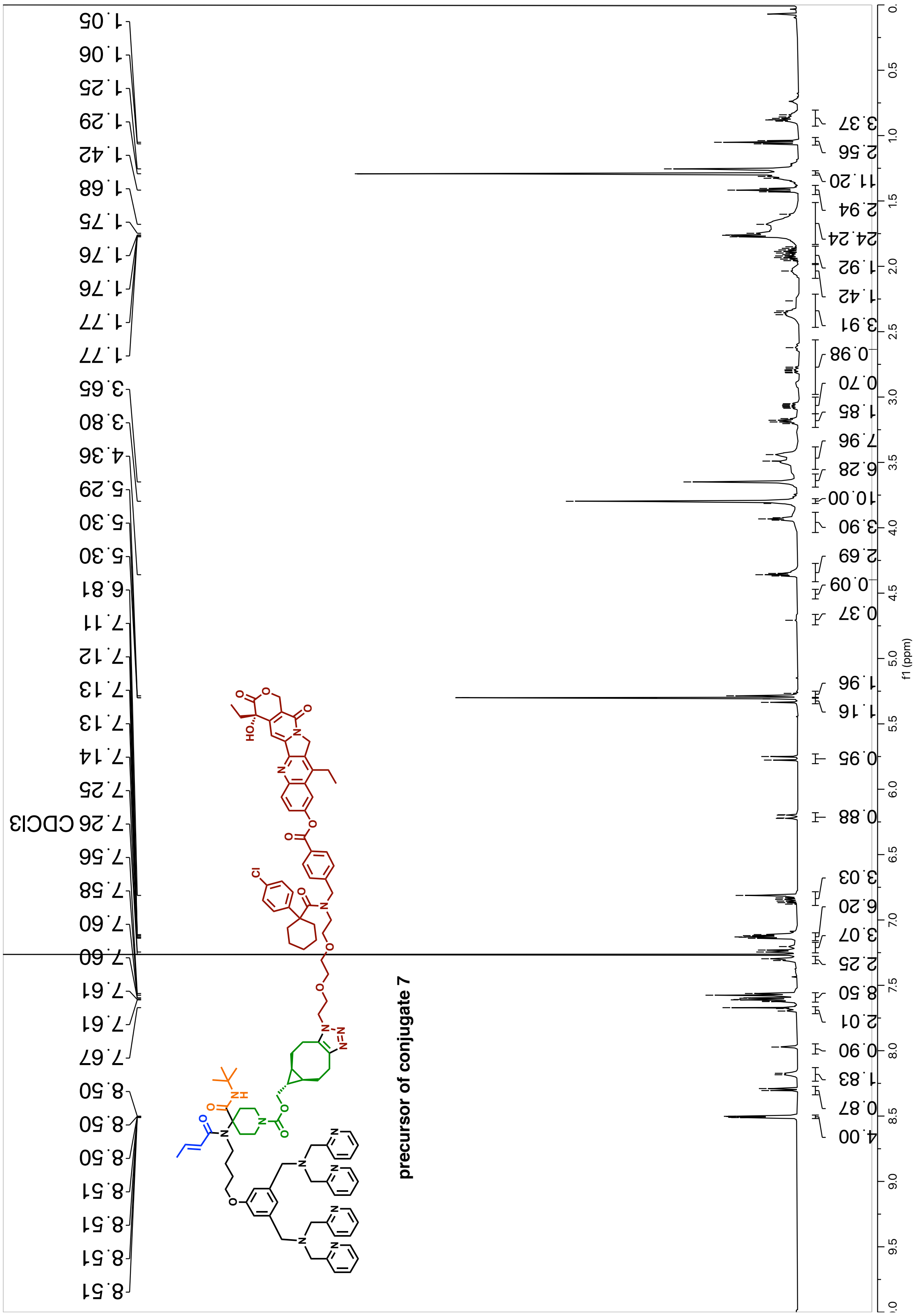

Figure S 45. The ^{1}H NMR of precursor of conjugate **7**.

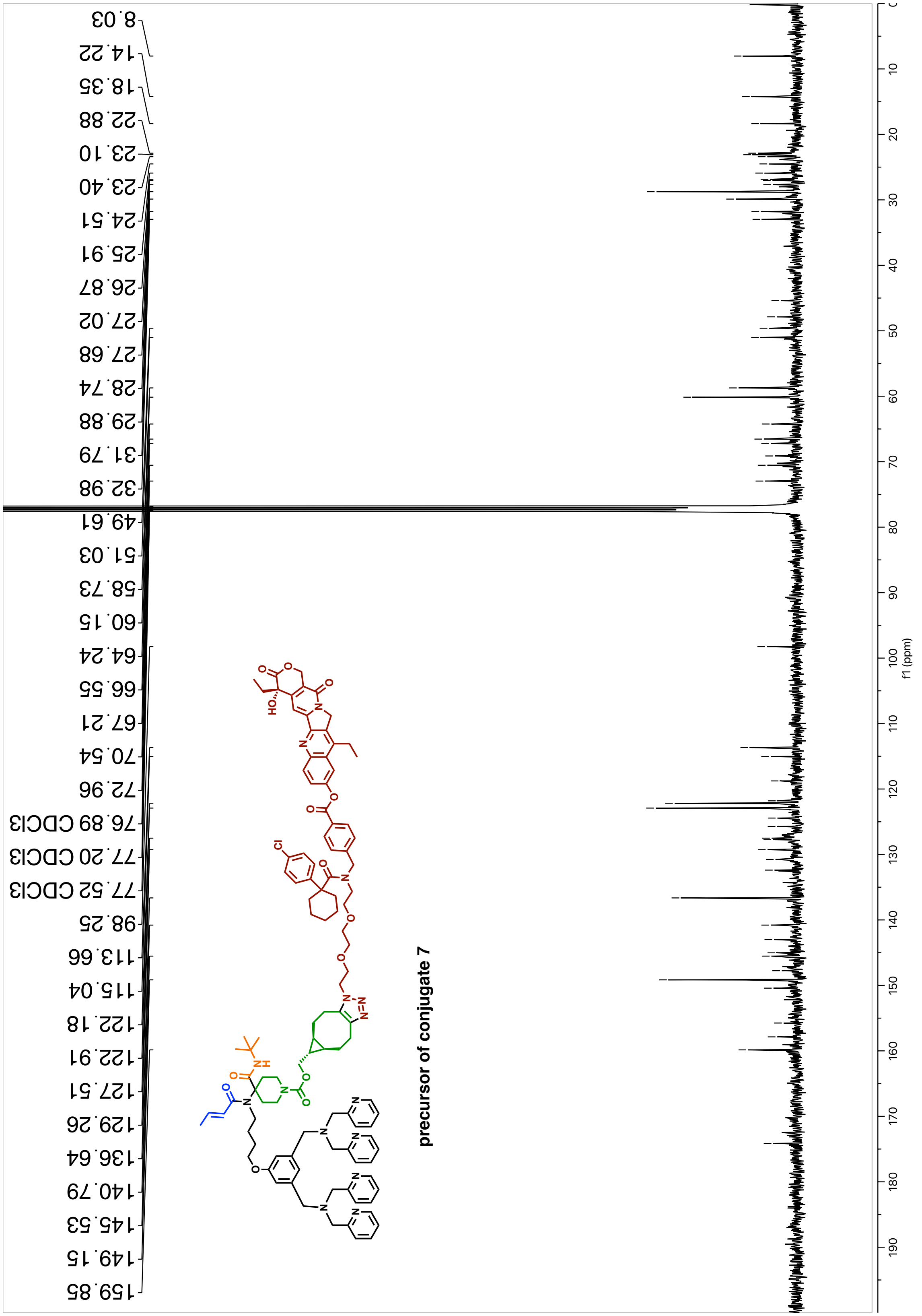

Figure S 46. The ^{13}C NMR of precursor of conjugate **7**.

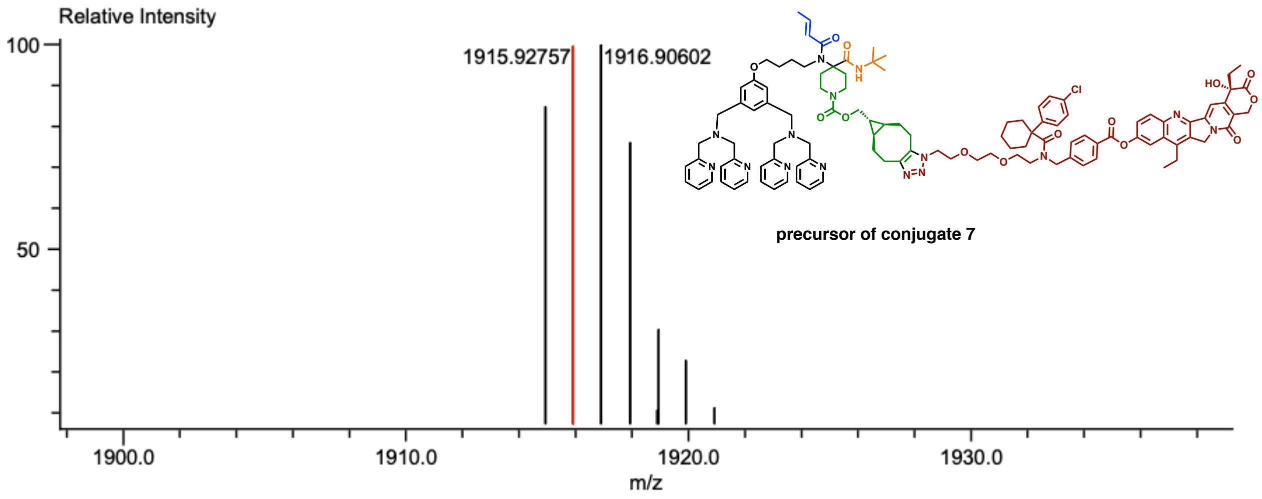

Mass	Intensity	Calc. Mass	Mass Difference [mDa]	Mass Difference [ppm]	Possible Formula
1915.92757	4842.40	1915.92972	-2.14	-1.12	$^{12}C_{110}{}^{1}H_{126}{}^{35}Cl_{1}{}^{14}N_{15}{}^{16}O_{14}$

Figure S 47. The high res spectra of precursor of conjugate **7**.

Spectra of precursor of conjugate 8.

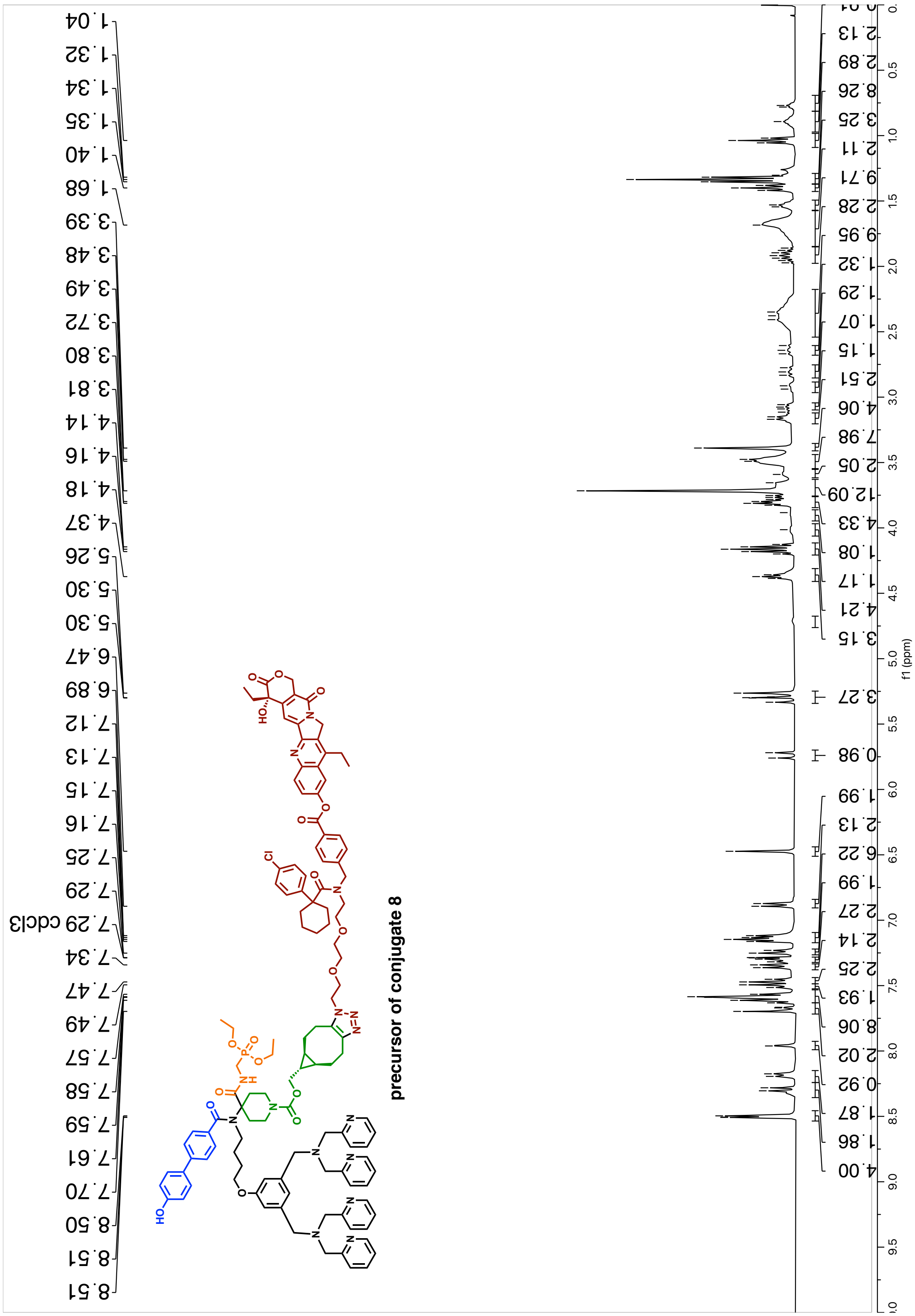

Figure S 48. The ^{1}H NMR of precursors of conjugate **8**.

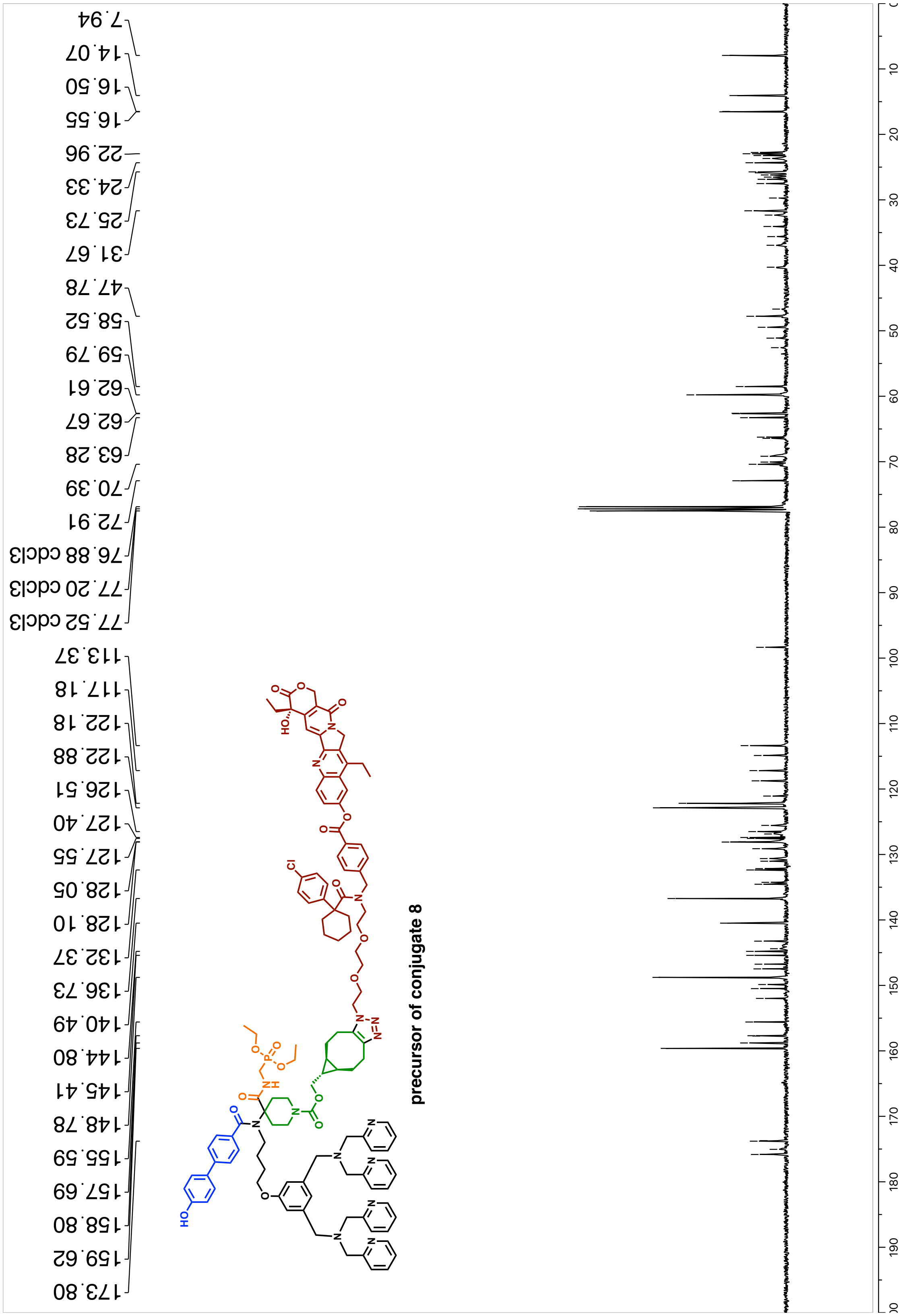

Figure S 49. The ^{13}C NMR of precursors of conjugate **8**.

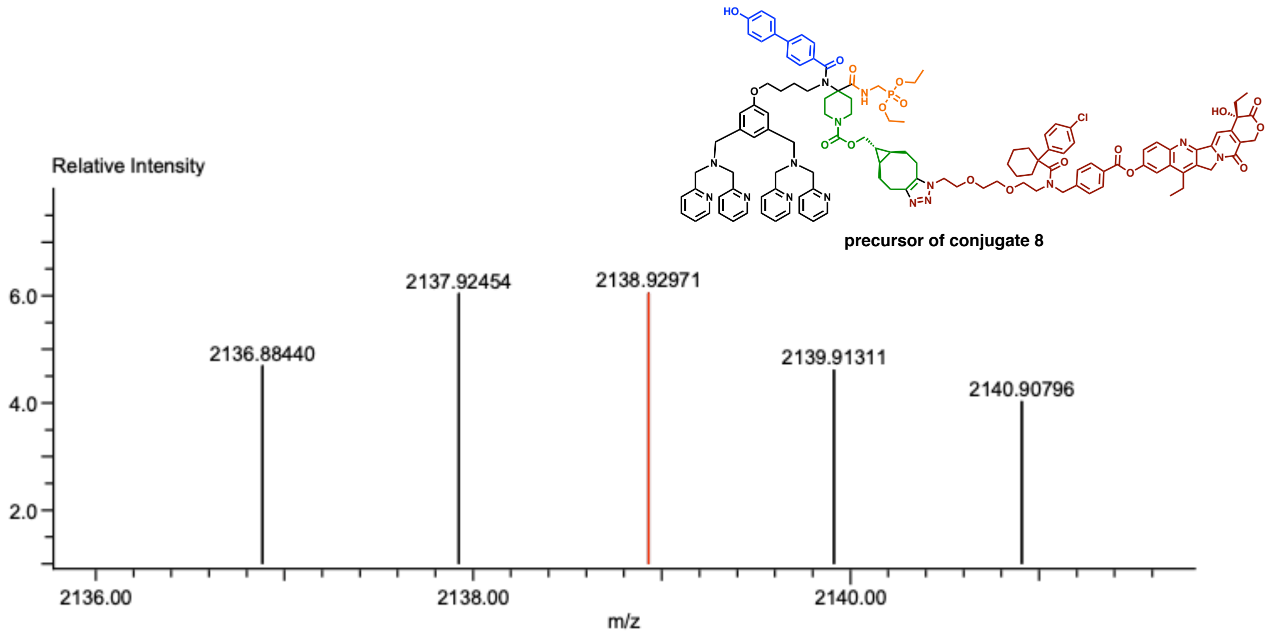

Mass	Intensity	Calc. Mass	Mass Difference [mDa]	Mass Difference [ppm]	Possible Formula
2138.92971	3173.10	2138.94574	-16.03	-7.49	$^{12}C_{120}{}^{1}H_{134}{}^{35}Cl_{1}{}^{14}N_{15}{}^{16}O_{18}{}^{31}P_{1}$

Figure S 50. The high res spectra of precursor of conjugate **8**.

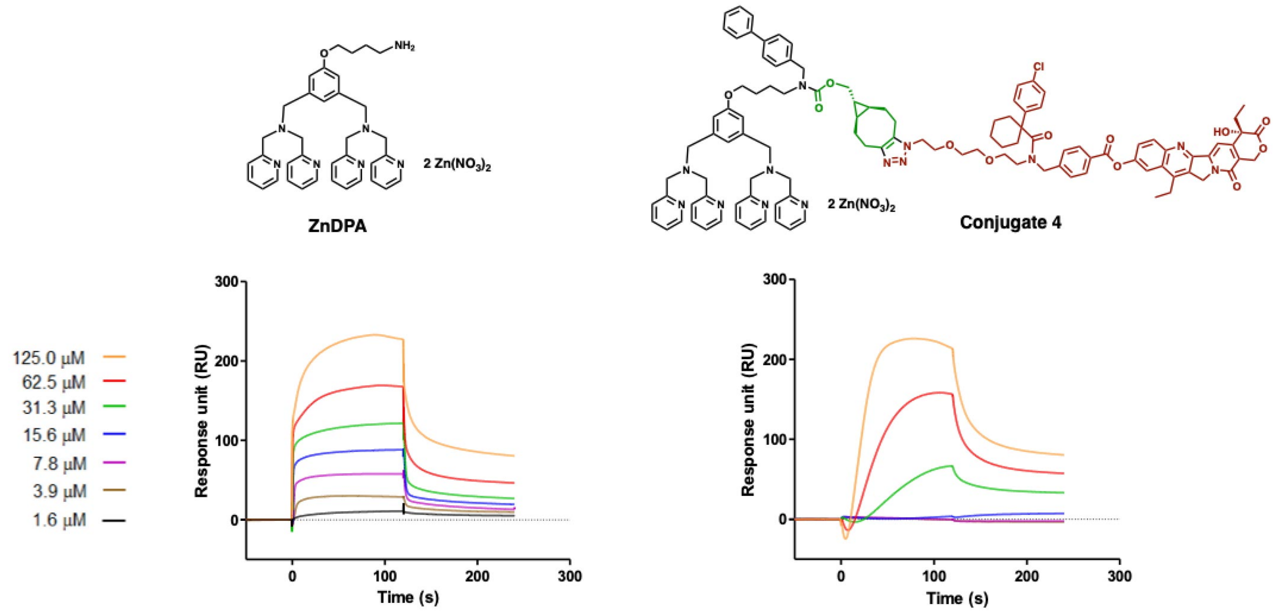

Figure S51. *In vitro* SPR PS-association studies. Sensorgrams generated using a Biacore T200. Conjugate **4** and ZnDPA were analyzed across a 2-fold concentration series descending from 125 μM. The association was monitored for 2 min, and the dissociation time was 2 min. Liposome (DOPC/DOPS (3:1,v/v)) was immobilized on a L1 chip at 2800 RU, where liposome of DOPC 100% was used as nonspecific binding control.
